# Unsupervised Clustering Reveals Sociodemographic Determinants of Differential Brain Development During Adolescence

**DOI:** 10.1002/hbm.70606

**Published:** 2026-07-12

**Authors:** Jiangyun Hou, Laurens van de Mortel, Arne Popma, Dirk J. A. Smit, Guido van Wingen

**Affiliations:** ^1^ Department of Psychiatry Amsterdam UMC Location University of Amsterdam Amsterdam the Netherlands; ^2^ Amsterdam Neuroscience Amsterdam the Netherlands; ^3^ Amsterdam Public Health Amsterdam the Netherlands

## Abstract

Adolescence is a critical period for brain development, impacting social, cognitive, and emotional functions. While hypothesis‐driven studies have linked multiple person characteristics to brain development, the driving force behind differential brain development remains unclear. We applied unsupervised clustering to multimodal neuroimaging data from early adolescents in the Adolescent Brain Cognitive Development (ABCD) study. Clustering analyses were conducted separately for resting‐state functional MRI (rs‐fMRI), structural MRI (sMRI), and diffusion MRI (dMRI), and replicated in two independent samples of 2666 individuals each. Longitudinal trajectories over a two‐year follow‐up period were examined. Associations with sociodemographic and family‐related factors were assessed. Two clusters were identified in rs‐fMRI data across both independent samples. One cluster, comprising approximately 9%–10% of individuals, showed functional brain differences at baseline, along with altered neurodevelopmental trajectories over 2 years. These functional differences were associated with lower socioeconomic status, family instability, and stronger cultural/family values. In contrast, no significant clustering emerged from structural MRI and diffusion MRI data. Our findings suggest that sociodemographic factors are closely associated with early adolescent brain function and development, underscoring the need to consider social environment in neurodevelopmental models and prevention strategies.

## Graphical abstract

Unsupervised clustering of resting‐state fMRI in a large adolescent cohort reveals distinct functional brain subgroups. These subgroups show divergent developmental trajectories and are associated with sociodemographic context, highlighting the role of social environment in shaping adolescent brain development.

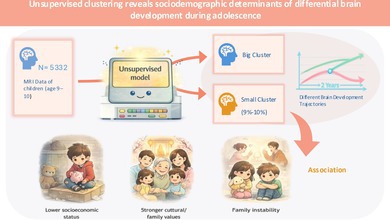

## 1 Introduction

Adolescence is an important period of brain development, a critical but vulnerable time for developing behaviors vital for adulthood. Neurodevelopment during this period has a profound impact on social and cognitive abilities, reward sensitivity, stress response, and decision‐making abilities of adolescents (Blakemore and Robbins 2012; Yahfoufi et al. 2020). This phase is also associated with heightened vulnerability to mental health problems, and many mental health disorders have their onset during adolescence. Many of these disorders are neurodevelopmental in nature (Kessler et al. 2007); therefore, understanding the neurodevelopmental trajectories during adolescence and the factors associated with these trajectories is essential for devising more effective guidelines for the promotion of healthy neurodevelopment among adolescents.

An increasing number of longitudinal studies have employed structural and functional magnetic resonance imaging (MRI) to capture ongoing developments during adolescence. These studies have revealed varied developmental patterns in both gray and white matter (Ashtari and Cyckowski 2011), with brain surface area and thickness exhibiting differential patterns of development (Vijayakumar et al. 2016) associated with cognitive and emotional growth. Several influences on these developmental patterns have been identified. For example, physical activity correlates with structural and functional properties of brain regions involved in emotional and behavioral control, and social interactions on digital platforms impact the anterior cingulate cortex and medial frontal cortex, which are linked to social processing (Belcher et al. 2021; Crone and Konijn 2018). Socioeconomic factors such as parental income, education, or occupation also play a role in the structural and functional development of cortical and subcortical regions (Brody et al. 2017; Tooley et al. 2021). And mental health conditions like conduct disorders, depression, and attention‐deficit/hyperactivity disorder (ADHD) are also associated with altered neurodevelopmental trajectories (Ellis et al. 2017; Fuelscher et al. 2023; Oostermeijer et al. 2016).

However, most findings are based on hypothesis‐driven studies that are intrinsically biased by pre‐set expectations. To avoid such biases, unsupervised learning, a method that identifies data patterns without predetermined targets, proves useful (Wang 2016), with many studies using it to cluster patients and identify distinct subtypes of a disorder (Amoretti et al. 2021; Stevens et al. 2019), or to use neuroimaging data to identify neurobiological clusters related to psychopathology and the diagnosis of neurodegenerative diseases (Liu et al. 2023; Wang et al. 2023). Despite advancements in unsupervised methods, few studies have linked neurobiological subtypes to longitudinal developmental trajectories. It therefore remains unclear whether neuroimaging profiles at the early stage of adolescence can predict diverse neurodevelopmental paths and which factors influence these patterns during adolescence. Identifying such connections provides a framework for understanding how initial neural profiles relate to subsequent cognitive, emotional, and mental health outcomes. This understanding can inform targeted interventions to support healthy neurodevelopment during this critical period.

In this study, we leveraged data from the Adolescent Brain Cognitive Development (ABCD) study, the largest ongoing longitudinal cohort of brain development in children and adolescents (Jernigan et al. 2018). Focusing on participants who did not present with clinically relevant mental health problems at baseline, we applied data‐driven, unsupervised learning approaches to multimodal neuroimaging data in order to identify neurobiological subtypes in adolescents. We then tracked their developmental trajectories over time and examined how these subtypes related to sociodemographic background, cognitive performance, and emerging mental health symptoms.

## 2 Materials and Methods

### 2.1 Participants

The ABCD Study, which enrolled around 11,876 children aged 9 to 10 at baseline, aims to investigate the neurodevelopmental trajectories of adolescence and their connections to behavior, environment, and genetics. By enrolling participants from a variety of schools and communities across the United States, the study achieves a diverse cohort that reflects a wide spectrum of socio‐economic, cultural, and geographic backgrounds. Comprehensive assessments are conducted on participants in the ABCD study, including neuropsychological testing, brain imaging, and detailed questionnaires regarding behavior, health, and lifestyle (Jernigan et al. 2018).

To mitigate the influence of preexisting mental health problems on brain measures, we selected ABCD study participants with initial t‐scores under 65 on the DSM‐oriented CBCL scales based on DSM‐5 criteria (Achenbach 2013). The CBCL is designed to detect abnormal behavior and exhibits a one‐tailed distribution. A score of 65 is used as a lenient criterion for identifying abnormal behavior and has good agreement with clinical diagnosis based on interview (Krol et al. 2006). This resulted in a sample of 8976 individuals without internalizing or externalizing problems at baseline. Then, we excluded those without usable MRI data at baseline and/or follow‐up (as large missing MRI datasets cannot be imputed), resulting in a final sample of 5354 individuals (Table 1). Data can be obtained via https://nda.nih.gov/abcd.

**TABLE 1:** Demographic data of two random samples.

	Sample1	Sample2	*p*_FDR
Sample size	2666	2667	
Age (mean ± SD)^a^	9.91 ± 0.62	9.91 ± 0.62	1.00
Gender Male (%)^b^	51.26%	51.18%	1.00
IQ (mean ± SD)^a^	102.28 ± 17.17	102.18 ± 17.36	1.00
EA (mean ± SD)^a^	16.83 ± 2.64	16.83 ± 2.62	1.00

^a^ *t*‐test.

^b^ *χ* ^2^.

### 2.2 Imaging Data

Imaging data included 1198 imaging features from three MRI modalities extracted from the ABCD dataset using the published protocol: resting state MRI (rsMRI), structural MRI (sMRI), diffusion tensor imaging (DTI). We selected 258 sMRI measures (including volume of subcortical ROIs and cortical thickness and cortical area of cortical ROIs), 84 DTI measures (including FA (FA) within DTI atlas tract and fiber tract volume within DTI atlas tracts), and 856 rsfMRI measures (including correlation within and between cortical networks, correlation between cortical networks and subcortical ROIs, temporal variance in subcortical ROIs and cortical ROIs and in Gordon parcellations) (Table S4). All preprocessing and analysis for these MRI measures were reported by Hagler et al. (2019).

### 2.3 Demographic Data

We included 2323 clinical features after excluding features with missing values greater than 10% from the longitudinal parent demographics survey of ABCD study. These features consist of 8 domains: general information, neurocognition, physical gender‐identity‐sexual‐health, gender identity/sexual health, culture/environment, substance use, novel technologies, and mental health (Table S5). Not all participants contain these features, and only a part of the samples with complete demographic data was used for correlation analysis.

### 2.4 Experimental Design and Statistical Analyses

Statistical analyses were performed in R. Separate linear models were used to assess the baseline measures for all samples in three modalities with imputed raked propensity weight (i.e., the raked propensity weight merged the American Community Survey (ACS) and ABCD data (with missing data imputed), estimated the propensity model, computed and scales/trims the propensity weights and finally raked the scaled weights to final ACS control totals by age, sex and race/ethnicity) and scanner model as covariates.

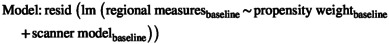




We randomly split the whole sample into two independent groups (1:1), sample 1 and sample 2, to validate the stability of the clustering results. Given the total number of features was 1198, we first used UMAP to reduce the dimensionality of baseline scores from sMRI, rsMRI, and DTI. UMAP, a graph‐based dimensionality reduction method, is widely used across various fields and is considered one of the state‐of‐the‐art techniques in this domain (Becht et al. 2019; Blanco‐Portals et al. 2022). Subsequently, we fed the dimensionally reduced data into HDBSCAN after tuning its hyperparameters (Blanco‐Portals et al. 2022). HDBSCAN is an unsupervised clustering algorithm that extends DBSCAN by constructing a hierarchical cluster tree based on density, and then extracting the most stable clusters from this hierarchy using a measure of cluster persistence (Blanco‐Portals et al. 2022). Unlike traditional clustering methods, HDBSCAN does not require the number of clusters to be specified in advance, and it is particularly well‐suited for high‐dimensional and noisy biological data where cluster shape and density may vary. This makes it an effective approach for discovering latent subgroups in complex neuroimaging datasets. This combination of UMAP and HDBSCAN has proven effective for clustering (Asyaky and Mandala 2021). To evaluate the robustness of the clustering pipeline, we additionally performed sensitivity analyses across a range of UMAP and HDBSCAN hyperparameter settings. Specifically, UMAP n_neighbors values of 50, 100, 150, and 200 and HDBSCAN min_cluster_size values of 75, 100, and 125 were tested while maintaining all other parameters identical to the original analysis. Clustering solutions obtained under each parameter combination were compared with the original manuscript solution using the Adjusted Rand Index (ARI).

We then assessed differences in baseline neural state and neural trajectories in three modalities between clusters using the linear models with false discovery rate (FDR) correction *p*(FDR) < 0.05/3 modalities (rsMRI, sMRI, DTI). To evaluate the factors related to our clusters, we selected samples with complete sociodemographical data (2323 clinical features from 8 domains as grouped by ABCD: general information, neurocognition, physical health, gender identity‐sexual health, culture‐environment, substance use, novel technologies and mental health) from the two samples separately. We performed ANOVA with FDR correction to identify significant differences in each sample for the eight different domains separately (p(FDR) < 0.05/8).

## 3 Results

**FIGURE 1:**
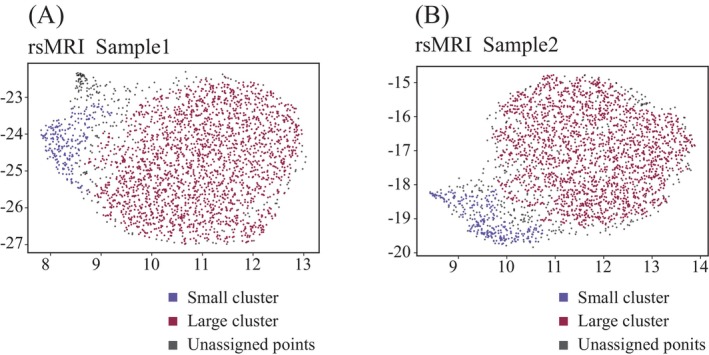
UMAP embedding of rsfMRI features in Sample 1 (A) and Sample 2 (B), with clusters identified by HDBSCAN. Each point represents an individual participant embedded in two‐dimensional UMAP space based on resting‐state fMRI (rsfMRI) features. Clustering was performed using Hierarchical Density‐Based Spatial Clustering of Applications with Noise (HDBSCAN), which identified three groups: A large cluster (red), a small cluster (blue), and unassigned individuals (gray). The unassigned individuals show low local density and insufficient stability across hierarchical scales. In Sample 1, the large cluster, small cluster, and unassigned individuals contained 2180 (81.8%), 233 (8.7%), and 253 (9.5%) participants, respectively. In Sample 2, the distribution was 2022 (75.8%), 270 (10.1%), and 375 (14.1%).

Using multimodal MRI data, we first explored clustering patterns across structural MRI (sMRI), diffusion tensor imaging (DTI), and resting‐state functional MRI (rsMRI) at baseline (Figure S1). Clear clustering was only observed in rsMRI, where dimensionality reduction followed by density‐based clustering consistently revealed two stable clusters across independent samples, alongside a set of unassigned individuals. Approximately 8%–10% of participants fell into the smaller cluster, 75%–82% into the larger cluster, and the remainder were unassigned. The unassigned individuals do not belong to any high‐density cluster, as determined by their low local density and lack of stability across scales. Specifically, in sample 1, the cluster sizes were *N* = 2180 (81.8%), *N* = 233 (8.7%), and *N* = 253 (9.5%; unassigned); in sample 2, *N* = 2022 (75.8%), *N* = 270 (10.1%), and *N* = 375 (14.1%; unassigned) (Figure 1). Unassigned individuals were diffusely distributed across low‐density regions and cluster boundaries within the UMAP embedding space rather than forming a spatially coherent subgroup, suggesting that these individuals likely represent intermediate or low‐confidence profiles rather than a distinct cluster. Sensitivity analyses demonstrated that the UMAP‐HDBSCAN clustering solution was relatively stable across a range of reasonable parameter settings in both independent samples (Figure S2). Across neighboring parameter combinations, clustering solutions showed moderate‐to‐high agreement with the original manuscript solution (ARI approximately 0.61–0.73). In addition, the smaller cluster remained consistently identifiable across parameter settings, suggesting that the major clustering structure was not driven by a single hyperparameter choice.

To characterize the neurobiological differences underlying these clusters, we compared rsMRI measures and found consistent group differences across a wide range of functional measures. We identified significant differences between the small and large cluster in 401 out of 856 rsMRI measures across both samples. Among them, 400 measures exhibited consistent effects in both samples, including temporal variance in different regions, correlations between and within networks, and correlations between networks and subcortical brain regions. Only temporal variance of the right postcentral gyrus showed opposite effects in the two samples. 48% of the significant measures showed higher values for functional activity and connectivity in the small cluster, and 52% showed lower values for functional activity and connectivity in the small cluster (Table S1).

Analysis of sMRI also revealed widespread differences between the clusters. We found that 102 out of 258 measures showed significant differences between the two clusters in both samples, all of which exhibited consistent effects. These included lower cortical thickness in the cuneus, isthmus cingulate, lingual, and lateral occipital regions; lower volumes in the cerebral white matter, cerebellum, hippocampus, amygdala, ventral diencephalon, caudate, and global brain measures; and reduced surface area in most cortical regions (Figure S3). In both samples, individuals from the small clusters showed lower values in these measures compared to individuals from the large cluster. In contrast, we did not observe any significant differences in DTI measures between the two clusters in either sample.

**FIGURE 2:**
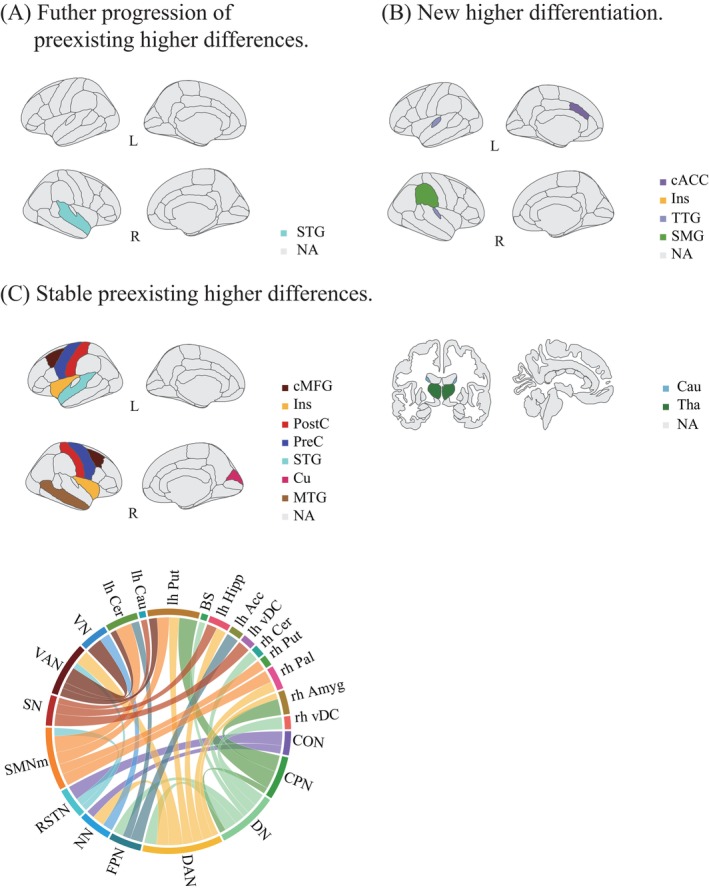
Higher functional differences in the small cluster at baseline and/or during development. (A) Cortical regions showing higher temporal variance in the small cluster at baseline and higher temporal variance changes at follow‐up (Baseline > 0, Development > 0), indicating further progression of preexisting differences; (B) Cortical regions showing no significant group differences at baseline but higher temporal variance changes during development (Baseline = 0, Development > 0), reflecting newly emerging differentiation; (C) Cortical and subcortical regions showing significantly higher temporal variance, and functional connectivity showing higher average correlations at baseline without further developmental differences (Baseline > 0, Development = 0), suggesting stable preexisting differences. Chord plots depict the involved functional networks and their changing connectivity patterns across time.

**FIGURE 3:**
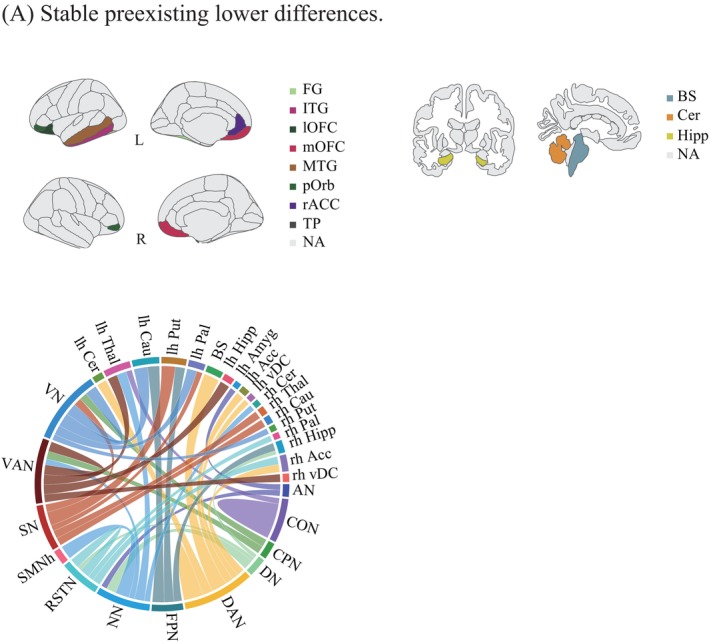
Lower functional differences in the small cluster at baseline with no developmental change. Cortical and subcortical regions showed lower temporal variance, and functional connectivity showed lower average correlations in the small cluster at baseline with no significant developmental changes (Baseline < 0, Development = 0), reflecting stable preexisting lower‐level differences. Chord plots depict the involved functional networks and their changing connectivity patterns across time.

We then examined neurodevelopmental trajectories over the 2‐year follow‐up period across the three imaging modalities. Only rsMRI measures exhibited significant changes between the two clusters over time. Based on baseline states and developmental trajectories, we categorized the features into four groups that were consistent across both samples: 1. One feature showed a pattern where the small cluster had higher *t* values at baseline and higher *t* values during development (Baseline > 0, Development > 0), indicating further progression of preexisting differences; 2. five features where the small cluster showed no significant differences at baseline and higher *t* values during development (Baseline = 0, Development > 0), indicating new differentiation; 3. seventy‐seven features where the small cluster showed higher *t* values at baseline with no significant differences in development (Baseline > 0, Development = 0), indicating stable preexisting differences (Figure 2.); 4. seventy‐five features where the small cluster showed lower *t* value at baseline with no significant differences in development (Baseline < 0, Development = 0), indicating stable preexisting differences (Figure 3.); 5. fifty‐eight features where the small cluster showed higher *t* values at baseline and lower *t* values during development (Baseline > 0, Development < 0), indicating normalization of preexisting differences; 6. ninety‐six features where the small cluster showed lower *t* value at baseline and higher *t* values during development (Baseline < 0, Development > 0), indicating normalization of preexisting differences (Figure S4). Some of the identified brain regions showed significant effects in multiple trajectory categories due to overlap across different brain atlases. For consistency and clarity, we classified such regions to the predominant pattern (e.g., higher *t*‐value), while reporting all atlas‐specific details in Table S2.

Finally, we investigated sociodemographic, cognitive, health, and psychological correlates of the clusters. We only used individuals with complete data for these features in both samples (sample1 *N* = 1659; sample 2 *N* = 1699) to investigate group differences using linear models. We found that 107 significant features overlapped between the two samples from general information, neurocognition, physical‐health, culture‐environment, substance‐use, novel‐technologies and mental‐health (*p* (FDR) < 0.05/8) (Table S3), which also showed consistent direction of effects. To identify the relation between the significant features, we generated heatmaps illustrating the correlation among features in the two independent samples. Using hierarchical clustering, features were organized into a tree‐like structure (dendrogram), allowing the identification of coherent feature groups at varying levels of granularity (Ran et al. 2023). This analysis identified three distinct categories of features that characterize the individuals from the small cluster (Figure S5). The first pattern related to socioeconomic and cultural background, including characteristics such as non‐White ethnicity (e.g., identified themselves as Hispanic/Latino/Latina), younger parental age, lower levels of education, lower family income, and lower cognitive test scores. Individuals within this group had fewer opportunities to participate in extracurricular activities, such as musical instrument training and soccer lessons. Additionally, they exhibited a strong connection to their ethnic identity, poorer eyesight, and perceptions of an unsafe neighborhood environment. In terms of family dynamics, this pattern was associated with a gentler communication style within the family and a stricter household environment regarding marijuana and alcohol use.

The second pattern is characterized by family instability, health, and lifestyle factors. Individuals in this category were more likely to identify themselves as Black/African American, come from households with lower economic stability, and report being not currently married. Their family environment was also more likely to involve exposure to smoking and parental marijuana use, while their health and lifestyle patterns included earlier physical maturation (e.g., accelerated height and body hair growth), shorter sleep duration, higher body weight and waist circumference, and increased caffeine consumption during childhood. Additionally, these individuals were more likely to have a second caregiver who was not their biological mother and to have half‐siblings, reflecting a greater degree of household instability. Psychological and behavioral attributes associated with this pattern included high parental expectations and support, goal‐driven behavior, specific phobias, and a greater perception of instability within the family environment.

The third pattern highlights strong religious and family‐oriented values, as individuals in the small cluster exhibited higher levels of religious commitment, a greater reliance on family as a referent for decision‐making, a deep sense of family obligation, and strong perceived family support. Comparisons between included and excluded participants revealed significant but small effects across key sociodemographic variables (mostly Cohen's d/Cramér's *V* < 0.1) (Table S5). Additional sensitivity analyses adjusting for race/ethnicity substantially attenuated most SES‐related associations, consistent with the strong overlap between ethnoracial and socioeconomic factors within the ABCD cohort (Tables S6 and S7). Furthermore, the majority of sociodemographic variables remained significantly associated with race/ethnicity after FDR correction in both independent subsets (Tables S8 and S9).

In summary, individuals from the small cluster showed significant neurodevelopmental differences in both brain structure and function compared with individuals from the large cluster, which was associated with differences related to socioeconomic and cultural backgrounds, family environment, health and psychological characteristics, and religious and family values in both samples.

## 4 Discussion

This study investigated whether different neurodevelopmental trajectories exist among adolescents aged 9–12 years with the use of unsupervised clustering of neuroimaging data. Our results revealed two distinct clusters of individuals based on rsfMRI data across two independent samples, while no significant clustering was observed in DTI and sMRI data. These clusters also exhibited significant differences in neurodevelopmental trajectories and were associated with multiple sociodemographic factors, including economic and cultural background, family environment, health traits, and religious and family values. Together, these results suggest that individuals from these sociodemographic backgrounds have other neurodevelopmental trajectories during adolescence.

From both samples, we observed distinct neurodevelopmental trajectories between two clusters with varying baseline functional differences and differential changes over the two‐year follow‐up. The most obvious differences emerged across large‐scale brain networks, rather than in isolated brain regions. In particular, the small cluster consistently exhibited higher functional connectivity values across multiple brain systems, which followed distinct temporal patterns. Some differences were already present at baseline, such as increased temporal variance in the right superior temporal gyrus, a region involved in social communication and reading, and previously linked to social economic states (SES) (Biazoli et al. 2020; Yovel and Belin 2013). Others emerged during development, including temporal variance increases in cortical regions related to auditory and social processing (Khalighinejad et al. 2021; Peng et al. 2021; Wada et al. 2021). A third group of differences was already present at baseline and showed little change over development. These involved higher correlations within and between several large‐scale networks, including the cingulo‐parietal, dorsal and ventral attention, default, and sensorimotor networks, as well as their connections to subcortical regions like the left putamen. These networks have previously been linked to attention allocation, cognitive and motor function, emotion regulation, but also to parental education (Assari 2021; Chang et al. 2023; Cosío‐Guirado et al. 2024; Dong et al. 2024; Ellwood‐Lowe et al. 2021). Despite their different onset times, these findings converge on a common pattern: adolescents in the small cluster show greater functional variability and connectivity across distributed networks that support cognitive, emotional, attentional, and motor functions. This suggests that socioeconomic disparities may relate to brain development not through isolated abnormalities, but via coordinated alterations in functional network organization beginning in early adolescence. Together, these results suggest that sociodemographic factors may influence adolescent development across multiple domains (cognitive, attentional, emotional, and motor systems) potentially through its impact on functional network and cortical/subcortical region variability.

In contrast to the widespread increases observed in the small cluster, another distinct pattern involved weaker correlations within the dorsal and ventral attention and visual networks, as well as their connections to subcortical regions. These reductions were already evident at baseline and showed no significant developmental change, indicating persistent underconnectivity in networks critical for attention and visual processing. In particular, lower within‐network correlations may reflect reduced intra‐network integration and processing efficiency. This underconnectivity extended to cortical and subcortical areas associated with language, memory, cognitive control, and emotion regulation (Belyk et al. 2017; Heather Hsu et al. 2020; Herlin et al. 2021; Jiang et al. 2015; Wang et al. 2024; Weiner and Zilles 2016; Zald et al. 2014), suggesting broad and stable functional differences. Additionally, lateralized developmental trajectories were observed, likely reflecting hemispheric specialization, and possibly influenced by handedness (Hellige 2001; Tzourio‐Mazoyer et al. 2020), which was not accounted for in this study.

However, another two patterns showed a trend toward normalization over development. Specifically, higher connectivity at baseline tends to decrease, while lower connectivity at baseline tends to increase, with both patterns converging toward a more balanced or stable level in auditory, sensorimotor, cingulo‐opercular, and retrosplenial temporal networks as well as subcortical regions.

To understand the factors underlying these functional patterns, we examined demographic variables associated with these clusters. ANOVA revealed that socioeconomic and cultural background, family environment, psychological and health traits, and religious and family values significantly differentiated the clusters in both samples. Adolescents in the small cluster were more likely to come from socioeconomically disadvantaged families, with lower parental education and income, younger parents, and greater family instability. This group also demonstrated lower cognitive performance, poorer sleep and health, higher body weight, and greater exposure to parental substance use and smoking. However, they also showed stronger ethnic identity, stricter household rules regarding substances, and higher levels of religious commitment and perceived family support.

These findings align with previous research showing that parental income and education are strong predictors of adolescent brain development (Assari 2021; Biazoli et al. 2020; Ellwood‐Lowe et al. 2021). Among these, family income is often the most robust SES‐related factor associated with neurodevelopmental and behavioral outcomes (Oshri et al. 2019). Lower parental education has been linked to increased parental stress and reduced parenting quality, both of which may contribute to adverse neural outcomes (Assari 2021). Our findings suggest that adolescents in the low‐SES cluster experienced more environmental adversity, which may partly explain their altered neurodevelopmental profiles.

Beyond economic disadvantage, the small cluster also showed strong cultural and family values, which likely reflect sociocultural, rather than biological influences. Prior studies have documented that ethnic minority children often face additional socio‐environmental challenges such as reduced access to quality education and healthcare (Dyer and Román‐Torres 2022; LaVeist et al. 2023; Lee and Singh 2021). The stronger ethnic identity observed in the small cluster may reflect factors such as generational status, cultural heritage, and protective racial socialization practices within minority families (Simon 2021). These values are often associated with higher family cohesion and support (Kiang and Fuligni 2009). Although few studies have directly examined the relationship between cultural identity, family values, and neurodevelopment, it is likely that these factors interact with socioeconomic adversity to influence brain functions involved in cognitive, emotional, and social processing. Importantly, these findings underscore the need for a comprehensive sociological perspective to understand adolescent neurodevelopment, recognizing that ethnicity and family cultural background are not just background variables but are components in developmental outcomes.

Although sMRI and DTI data did not yield meaningful clusters from unsupervised methods, sMRI differences were nonetheless observed between the rsfMRI‐derived clusters. This suggests that while functional differences are more sensitive to sociodemographic‐related influences in early adolescence, structural brain changes may co‐occur or follow functional changes. Indeed, previous studies have shown that socioeconomic status can influence brain structure, as detected by both DTI and sMRI (Assari and Boyce 2021; Tooley et al. 2021). However, these effects on brain structure may be slower to emerge than those on brain function, potentially explaining the absence of clear clustering in structural modalities. Importantly, the absence of robust sMRI clustering does not imply the absence of structural group differences. Unsupervised clustering identifies stable multivariate subgroup structure, whereas post hoc group comparisons can detect distributed but relatively modest mean differences that may not be sufficiently strong or separable to generate stable clusters. Furthermore, our analyses were based on preprocessed summary data from the ABCD Study rather than raw imaging data, which may have limited the sensitivity of unsupervised clustering applied to DTI and sMRI measures.

The use of unsupervised clustering in this study represents a key methodological strength. By not relying on predefined groupings such as SES, this approach allowed the natural structure of the data to guide subgroup formation. Despite not being involved in clustering, the emergence of SES as a primary explanatory factor provides strong and data‐driven evidence that sociodemographic status plays an important role in adolescent neurodevelopment. These results emphasize that sociodemographic status is not a background covariate but a deeply embedded factor influencing brain functions.

Nonetheless, several limitations should be noted. First, the study is based on data from North American children, limiting generalizability to other cultures and continents. Second, the age range (9–10 years) constrains our ability to infer patterns beyond early adolescence. Third, although comparisons between included and excluded participants revealed only small sociodemographic effect sizes, some degree of selection bias related to imaging completeness cannot be fully excluded. In addition, we did not impute missing MRI‐derived measures, as imputation of high‐dimensional neuroimaging features could artificially bias unsupervised clustering solutions. Forth, while we used propensity score matching and other controls, racial differences between clusters persisted. While racial differences in brain structure remain even after accounting for disparities in sociodemographic factors in hypothesis‐driven research, our results did not reveal robust unsupervised clustering based on sMRI data (Dumornay et al. 2023). This suggests that the clustering based on rsMRI data is more likely to reflect environmental rather than biological effects. In population‐based cohorts such as ABCD, ethnoracial background is deeply intertwined with socioeconomic conditions, neighborhood environment, educational access, cultural identity, and family structure. As a result, fully dissociating socioeconomic and ethnoracial effects statistically may not be feasible and could lead to over‐adjustment of the broader social and environmental constructs under investigation. Nevertheless, these results highlight the importance of interpreting race not as a fixed biological variable but as a proxy for environmental exposures and social context. Finally, given the observational design of the study, the present findings should not be interpreted causally. The observed associations between sociodemographic factors and neurodevelopmental patterns may reflect bidirectional or shared relationships involving environmental exposures, genetic liability, neighborhood‐level stressors, or other unmeasured variables.

In conclusion, this longitudinal study underscores the close relationship between sociodemographic factors and adolescent brain development. Our results support the view that these factors are embedded in functional brain architecture by early adolescence, influencing trajectories in cognition, emotion, and social processing. A socioecological framework is essential for understanding the developmental impact of these early‐life experiences.

## Author Contributions

**Jiangyun Hou:** methodology, software, validation, visualization, and writing – original draft. **Laurens van de Mortel:** methodology, validation, and writing – review and editing. **Dirk J. A. Smit:** methodology, and writing – review and editing. **Guido van Wingen:** methodology, supervision, writing – review and editing.

## Ethics Statement

All human participants' data were obtained from the Adolescent Brain Cognitive Development (ABCD) Study. The ABCD Study adheres to ethical standards for research involving human participants, with informed consent and assent obtained from all participants and their legal guardians. The study was approved by the Institutional Review Board at the University of California, San Diego (IRB #160091). The present analyses used only de‐identified data (release 5.0) available from the National Institute of Mental Health Data Archive (NDA) and were therefore exempt from additional review by the authors' institution. For ethics‐related queries, please contact the UCSD Institutional Review Board at irb@ucsd.edu.

## Conflicts of Interest

Guido van Wingen has received research support from Biogen, Bitbrain, Philips, and GH Research for projects unrelated to this work.

## Supporting information


**Figure S1:** UMAP embedding of MRI data across two independent samples.
**Figure S2:** Stability of the UMAP‐HDBSCAN clustering solution across different hyperparameter settings in Sample 1 and Sample 2. Heatmaps show the Adjusted Rand Index (ARI) between clustering solutions generated using different combinations of UMAP n_neighbors (50, 100, 150, 200) and HDBSCAN min_cluster_size (75, 100, 125) and the original manuscript solution (UMAP n_neighbors = 200; HDBSCAN min_cluster_size = 100). Higher ARI values indicate greater similarity to the original clustering structure. Across both independent samples, the major clustering structure remained relatively stable across neighboring parameter settings.
**Figure S3:** Preexisting structural differences in sMRI between clusters.
**Figure S4:** Normalization patterns of preexisting functional differences over time. (A) Functional connectivity showed higher average correlations at baseline and lower values during development in the small cluster (Baseline > 0, Development < 0), indicating normalization of preexisting higher differences; (B) Functional connectivity showed lower average correlations at baseline and higher average correlations changes during development (Baseline < 0, Development > 0), indicating normalization of preexisting lower differences. Chord plots depict the involved functional networks and their changing connectivity patterns across time.
**Figure S5:** Correlation structure and hierarchical clustering of significant sociodemographic and behavioral features.
**Table S1:** Significant rs fMRI features between two clusters at baseline from two independent samples.
**Table S2:** Different patterns of rs fMRI features for small cluster compared to large cluster from two independent samples.
**Table S2:** Significant clinical features and directions to label from two independent samples.
**Table S3:** The selected MRI measures.
**Table S4:** Number of demographic features included.
**Table S5:** Demographic comparation between included individuals and excluded individuals.
**Table S6:** Sensitivity analyses of subset1 examining associations between cluster assignment and sociodemographic variables after adjustment for race/ethnicity.
**Table S7:** Sensitivity analyses of subset2 examining associations between cluster assignment and sociodemographic variables after adjustment for race/ethnicity.
**Table S8:** Associations between race/ethnicity and all sociodemographic variables included in the post hoc analyses in subset 1. Continuous variables were tested using one‐way ANOVA and categorical variables using *χ* ^2^ tests. Effect sizes are reported as eta‐squared for continuous variables and Cramér's V for categorical variables. *p* values were corrected for multiple comparisons using false discovery rate (FDR) correction.
**Table S9:** Associations between race/ethnicity and all sociodemographic variables included in the post hoc analyses in subset 2. Continuous variables were tested using one‐way ANOVA and categorical variables using *χ* ^2^ tests. Effect sizes are reported as eta‐squared for continuous variables and Cramér's V for categorical variables. *p* values were corrected for multiple comparisons using false discovery rate (FDR) correction.

## Data Availability

Data used in the preparation of this article can be obtained from the Adolescent Brain Cognitive Development (ABCD) Study (https://abcdstudy.org), held in the NIMH Data Archive (NDA).
