# Supplementary material for: Unsupervised Clustering Reveals Sociodemographic Determinants of Differential Brain Development During Adolescence
Source: Hum Brain Mapp. 2026 Jul 12;47(10):e70606. doi: 10.1002/hbm.70606 (PMC13358016; doi:10.1002/hbm.70606)
Supplement: Supplementary file 1 — Figure S1: UMAP embedding of MRI data across two independent samples. Figure S2: Stability of the UMAP‐HDBSCAN clustering solution across different hyperparameter settings in Sample 1 and Sample 2. Heatmaps show the Adjusted Rand Index (ARI) between clustering solutions generated using different combinations of UMAP n_neighbors (50, 100, 150, 200) and HDBSCAN min_cluster_size (75, 100, 125) and the original manuscript solution (UMAP n_neighbors = 200; HDBSCAN min_cluster_size = 100). Higher ARI values indicate greater similarity to the original clustering structure. Across both independent samples, the major clustering structure remained relatively stable across neighboring parameter settings. Figure S3: Preexisting structural differences in sMRI between clusters. Figure S4: Normalization patterns of preexisting functional differences over time. (A) Functional connectivity showed higher average correlations at baseline and lower values during development in the small cluster (Baseline > 0, Development < 0), indicating normalization of preexisting higher differences; (B) Functional connectivity showed lower average correlations at baseline and higher average correlations changes during development (Baseline < 0, Development > 0), indicating normalization of preexisting lower differences. Chord plots depict the involved functional networks and their changing connectivity patterns across time. Figure S5: Correlation structure and hierarchical clustering of significant sociodemographic and behavioral features. Table S1: Significant rs fMRI features between two clusters at baseline from two independent samples. Table S2: Different patterns of rs fMRI features for small cluster compared to large cluster from two independent samples. Table S2: Significant clinical features and directions to label from two independent samples. Table S3: The selected MRI measures. Table S4: Number of demographic features included. Table S5: Demographic comparation between included individual [file HBM-47-e70606-s001.docx]

Supplementary Materials for

Unsupervised clustering reveals sociodemographic determinants of differential brain development during adolescence

Jiangyun Hou, Laurens van de Mortel, Dirk Smit, Arne Popma,

Guido van Wingen

Corresponding author: Jiangyun Hou and Guido van Wingen

E-mail: [j.hou@amsterdamumc.nl](mailto:j.hou@amsterdamumc.nl)

E-mail: [g.a.vanwingen@amsterdamumc.nl](mailto:g.a.vanwingen@amsterdamumc.nl)

**The file includes:**

Figs. S1 to S5

Tables. S1 to S7

**Fig. S1. UMAP embedding of MRI data across two independent samples.**

UMAP was used to visualize the low-dimensional structure of rsfMRI, sMRI, and DTI baseline features in Sample 1 and Sample 2; (A1, A2) rsfMRI data revealed visually separable cluster structures in both samples; (B1, B2) sMRI and (C1, C2) DTI data showed no clear clustering.

**Fig. S2.** Stability of the UMAP-HDBSCAN clustering solution across different hyperparameter settings in Sample 1 and Sample 2. Heatmaps show the Adjusted Rand Index (ARI) between clustering solutions generated using different combinations of UMAP n_neighbors (50, 100, 150, 200) and HDBSCAN min_cluster_size (75, 100, 125) and the original manuscript solution (UMAP n_neighbors = 200; HDBSCAN min_cluster_size = 100). Higher ARI values indicate greater similarity to the original clustering structure. Across both independent samples, the major clustering structure remained relatively stable across neighboring parameter settings.

**Fig. S3. Preexisting structural differences in sMRI between clusters.**

Cortical and subcortical regions showing significant structural differences between the large and small clusters are visualized. Findings were consistent across both samples, including reduced cortical thickness in the cuneus, isthmus cingulate, lingual, and lateral occipital regions, lower volumes in white matter, cerebellum, hippocampus, amygdala, ventral diencephalon, caudate, and reduced cortical surface area in most brain regions.

**Fig. S4. Normalization patterns of preexisting functional differences over time.** (A) Functional connectivity showed higher average correlations at baseline and lower values during development in the small cluster (Baseline > 0, Development < 0), indicating normalization of preexisting higher differences; (B) Functional connectivity showed lower average correlations at baseline and higher average correlations changes during development (Baseline < 0, Development > 0), indicating normalization of preexisting lower differences. Chord plots depict the involved functional networks and their changing connectivity patterns across time.

**Fig.S5.** **Correlation structure and hierarchical clustering of significant sociodemographic and behavioral features.**

Heatmaps display the pairwise Pearson correlations between significant features across individuals in Sample 1 (A) and Sample 2 (B). Features were grouped using hierarchical clustering, and three distinct clusters emerged consistently across samples: Socioeconomic and cultural background, Family instability, health, and lifestyle and Religious and family-oriented values. These patterns provide a structured view of how sociodemographic and lifestyle factors covary, and how they relate to clusters.

**Table S1. Significant rs fMRI features between two clusters at baseline from two independent samples.**

| region | t_values_subset1 | p_fdr_ subset1 | t_values_ subset2 | p_fdr_ subset2 |
| --- | --- | --- | --- | --- |
| rsfmri_var_cortgordon_gp212rh | -2.839368186 | 0.008423003943511 | 4.60457380492317 | 1.04995817852835E-05 |
| rsfmri_c_ngd_ad_ngd_ad | 8.81067547563401 | 1.31485183616612E-17 | 12.2412892739484 | 1.6232309219346E-32 |
| rsfmri_c_ngd_ad_ngd_cgc | -7.435935259 | 6.71033510363438E-13 | -6.82098227 | 4.26580939969249E-11 |
| rsfmri_c_ngd_ad_ngd_ca | -10.62016712 | 6.13388527667797E-25 | -9.638245954 | 8.00552660967949E-21 |
| rsfmri_c_ngd_ad_ngd_dt | -12.75501592 | 3.66161220919042E-35 | -12.32589549 | 6.14268448636552E-33 |
| rsfmri_c_ngd_ad_ngd_fo | -17.65153209 | 1.90251472295727E-64 | -22.85015535 | 9.18610028296767E-103 |
| rsfmri_c_ngd_ad_ngd_n | -3.513491252 | 0.000974220627674 | -4.46809623 | 1.94903998423803E-05 |
| rsfmri_c_ngd_ad_ngd_rspltp | 7.1410522356492 | 5.32799253154352E-12 | 9.77499128356893 | 2.3164120010175E-21 |
| rsfmri_c_ngd_ad_ngd_smh | 29.4598454180347 | 3.24797905827591E-161 | 31.2971603269768 | 3.23946532531658E-177 |
| rsfmri_c_ngd_ad_ngd_smm | 17.2649503523891 | 6.82061820122399E-62 | 19.1456438974515 | 1.17710133069424E-74 |
| rsfmri_c_ngd_ad_ngd_sa | -18.23430058 | 2.07028402935046E-68 | -18.51338986 | 3.22467098532403E-70 |
| rsfmri_c_ngd_ad_ngd_vs | 18.1548133906315 | 7.09885012609724E-68 | 19.0830909055897 | 3.18089027712073E-74 |
| rsfmri_c_ngd_cgc_ngd_ad | -7.435935259 | 6.71033510363438E-13 | -6.82098227 | 4.26580939969249E-11 |
| rsfmri_c_ngd_cgc_ngd_cgc | -9.56712052 | 1.66330367878324E-20 | -9.207622346 | 3.84427364302845E-19 |
| rsfmri_c_ngd_cgc_ngd_n | 4.0228910334032 | 0.000145592927963 | 4.82549047922714 | 3.76674012026353E-06 |
| rsfmri_c_ngd_cgc_ngd_rspltp | 7.50421718858897 | 4.15531595047249E-13 | 8.81506232069689 | 1.16877113749882E-17 |
| rsfmri_c_ngd_cgc_ngd_sa | -4.102225819 | 0.000106965137287 | -3.771500833 | 0.000350096350079 |
| rsfmri_c_ngd_cgc_ngd_vs | 3.89938329159247 | 0.000235912742039 | 4.85080640160201 | 3.35058473861478E-06 |
| rsfmri_c_ngd_ca_ngd_ad | -10.62016712 | 6.13388527667797E-25 | -9.638245954 | 8.00552660967949E-21 |
| rsfmri_c_ngd_ca_ngd_dt | 3.82924600119915 | 0.000304764823738 | 3.77872742366172 | 0.000341846435866 |
| rsfmri_c_ngd_ca_ngd_fo | 7.16160017525437 | 4.64918913708825E-12 | 5.38922791226925 | 2.19659012237194E-07 |
| rsfmri_c_ngd_ca_ngd_smh | -12.47403313 | 1.00735931218551E-33 | -9.109110125 | 9.04781784289247E-19 |
| rsfmri_c_ngd_ca_ngd_smm | -11.49071887 | 6.65651584373882E-29 | -8.661460869 | 4.22950571540982E-17 |
| rsfmri_c_ngd_ca_ngd_sa | 9.06587556851785 | 1.45563781334035E-18 | 6.28833008936878 | 1.24391785744257E-09 |
| rsfmri_c_ngd_ca_ngd_vta | -4.780194379 | 5.31493955470563E-06 | -6.666008596 | 1.18357367949167E-10 |
| rsfmri_c_ngd_ca_ngd_vs | -4.963515664 | 2.17895800909771E-06 | -4.118762879 | 8.82417628055702E-05 |
| rsfmri_c_ngd_dt_ngd_ad | -12.75501592 | 3.66161220919042E-35 | -12.32589549 | 6.14268448636552E-33 |
| rsfmri_c_ngd_dt_ngd_ca | 3.82924600119915 | 0.000304764823738 | 3.77872742366172 | 0.000341846435866 |
| rsfmri_c_ngd_dt_ngd_dla | 6.59781766184634 | 2.06095871634814E-10 | 7.71433024294348 | 7.70710389149546E-14 |
| rsfmri_c_ngd_dt_ngd_fo | 5.24554100879092 | 5.2567911882808E-07 | 4.21388907416603 | 5.94280679954199E-05 |
| rsfmri_c_ngd_dt_ngd_n | -5.542865531 | 1.08328988474855E-07 | -6.61659628 | 1.60377695001897E-10 |
| rsfmri_c_ngd_dt_ngd_rspltp | -2.943603246 | 0.006214259040589 | -4.348659397 | 3.32098667701225E-05 |
| rsfmri_c_ngd_dt_ngd_smh | -14.14532275 | 1.16154778910124E-42 | -14.81136727 | 1.96429544481277E-46 |
| rsfmri_c_ngd_dt_ngd_smm | -7.558476992 | 2.81640271420573E-13 | -8.215152631 | 1.60334325244991E-15 |
| rsfmri_c_ngd_dt_ngd_vta | -7.474361257 | 5.10306559199606E-13 | -8.533059636 | 1.21469329394602E-16 |
| rsfmri_c_ngd_dla_ngd_dt | 6.59781766184634 | 2.06095871634814E-10 | 7.71433024294348 | 7.70710389149546E-14 |
| rsfmri_c_ngd_dla_ngd_dla | -5.791816024 | 2.74292677075795E-08 | -6.331132531 | 9.7008965824899E-10 |
| rsfmri_c_ngd_dla_ngd_n | 6.33046144820029 | 1.1233904615551E-09 | 7.30901960292537 | 1.50656389601698E-12 |
| rsfmri_c_ngd_dla_ngd_vta | 7.84742346471843 | 3.17026764596153E-14 | 7.87050487196811 | 2.39612605168255E-14 |
| rsfmri_c_ngd_dla_ngd_vs | -7.101471237 | 6.95170164643606E-12 | -6.19421615 | 2.22229520033836E-09 |
| rsfmri_c_ngd_fo_ngd_ad | -17.65153209 | 1.90251472295727E-64 | -22.85015535 | 9.18610028296767E-103 |
| rsfmri_c_ngd_fo_ngd_ca | 7.16160017525437 | 4.64918913708825E-12 | 5.38922791226925 | 2.19659012237194E-07 |
| rsfmri_c_ngd_fo_ngd_dt | 5.24554100879092 | 5.2567911882808E-07 | 4.21388907416603 | 5.94280679954199E-05 |
| rsfmri_c_ngd_fo_ngd_fo | 5.07372007057702 | 1.25256381256429E-06 | 5.58234071577097 | 7.64956663257355E-08 |
| rsfmri_c_ngd_fo_ngd_smh | -22.61770566 | 2.34147288669735E-101 | -23.72521703 | 7.05089976219023E-110 |
| rsfmri_c_ngd_fo_ngd_smm | -13.3762499 | 1.97461139556729E-38 | -15.87374972 | 7.55857983529618E-53 |
| rsfmri_c_ngd_fo_ngd_sa | 6.64750795261643 | 1.50268396513163E-10 | 7.16436832396338 | 4.1330661328599E-12 |
| rsfmri_c_ngd_fo_ngd_vta | -4.62513171 | 1.09152839972059E-05 | -5.348608757 | 2.71664987573368E-07 |
| rsfmri_c_ngd_fo_ngd_vs | -5.582617565 | 8.78619014914633E-08 | -5.114841113 | 9.17976552888048E-07 |
| rsfmri_c_ngd_n_ngd_ad | -3.513491252 | 0.000974220627674 | -4.46809623 | 1.94903998423803E-05 |
| rsfmri_c_ngd_n_ngd_cgc | 4.0228910334032 | 0.000145592927963 | 4.82549047922714 | 3.76674012026353E-06 |
| rsfmri_c_ngd_n_ngd_dt | -5.542865531 | 1.08328988474855E-07 | -6.61659628 | 1.60377695001897E-10 |
| rsfmri_c_ngd_n_ngd_dla | 6.33046144820029 | 1.1233904615551E-09 | 7.30901960292537 | 1.50656389601698E-12 |
| rsfmri_c_ngd_n_ngd_smh | -7.299801293 | 1.76281371701774E-12 | -9.249370168 | 2.65679213433015E-19 |
| rsfmri_c_ngd_n_ngd_vta | -3.901245234 | 0.000235435651993 | -4.526239163 | 1.50731748682194E-05 |
| rsfmri_c_ngd_n_ngd_vs | 5.92257101327243 | 1.31084812931926E-08 | 3.08057810683403 | 0.003846443365608 |
| rsfmri_c_ngd_rspltp_ngd_ad | 7.1410522356492 | 5.32799253154352E-12 | 9.77499128356893 | 2.3164120010175E-21 |
| rsfmri_c_ngd_rspltp_ngd_cgc | 7.50421718858897 | 4.15531595047249E-13 | 8.81506232069689 | 1.16877113749882E-17 |
| rsfmri_c_ngd_rspltp_ngd_dt | -2.943603246 | 0.006214259040589 | -4.348659397 | 3.32098667701225E-05 |
| rsfmri_c_ngd_rspltp_ngd_rspltp | -5.500685349 | 1.36059059178488E-07 | -2.897303764 | 0.006604876123871 |
| rsfmri_c_ngd_rspltp_ngd_smh | 5.90462573471201 | 1.44720721348086E-08 | 6.88388534412017 | 2.85924780521883E-11 |
| rsfmri_c_ngd_rspltp_ngd_smm | 4.04280326892946 | 0.000135007610716 | 7.01971664368531 | 1.13901717577485E-11 |
| rsfmri_c_ngd_rspltp_ngd_vta | 3.14428522334377 | 0.00338649770742 | 3.1625088960319 | 0.002973556350552 |
| rsfmri_c_ngd_smh_ngd_ad | 29.4598454180347 | 3.24797905827591E-161 | 31.2971603269768 | 3.23946532531658E-177 |
| rsfmri_c_ngd_smh_ngd_ca | -12.47403313 | 1.00735931218551E-33 | -9.109110125 | 9.04781784289247E-19 |
| rsfmri_c_ngd_smh_ngd_dt | -14.14532275 | 1.16154778910124E-42 | -14.81136727 | 1.96429544481277E-46 |
| rsfmri_c_ngd_smh_ngd_fo | -22.61770566 | 2.34147288669735E-101 | -23.72521703 | 7.05089976219023E-110 |
| rsfmri_c_ngd_smh_ngd_n | -7.299801293 | 1.76281371701774E-12 | -9.249370168 | 2.65679213433015E-19 |
| rsfmri_c_ngd_smh_ngd_rspltp | 5.90462573471201 | 1.44720721348086E-08 | 6.88388534412017 | 2.85924780521883E-11 |
| rsfmri_c_ngd_smh_ngd_smh | 32.9966987340964 | 5.3221948201104E-195 | 31.8021866178374 | 7.32676325319659E-182 |
| rsfmri_c_ngd_smh_ngd_smm | 37.0972585637301 | 6.70397543070745E-236 | 36.2770100997169 | 2.00219004984975E-225 |
| rsfmri_c_ngd_smh_ngd_sa | -18.8951899 | 4.81565233623175E-73 | -17.86095263 | 9.66350810914392E-66 |
| rsfmri_c_ngd_smh_ngd_vta | 5.39052445358394 | 2.48272852336853E-07 | 5.09723818153208 | 9.9699166455301E-07 |
| rsfmri_c_ngd_smh_ngd_vs | 20.1464943139387 | 3.627419731637E-82 | 21.0991500651214 | 4.70843346566054E-89 |
| rsfmri_c_ngd_smm_ngd_ad | 17.2649503523891 | 6.82061820122399E-62 | 19.1456438974515 | 1.17710133069424E-74 |
| rsfmri_c_ngd_smm_ngd_ca | -11.49071887 | 6.65651584373882E-29 | -8.661460869 | 4.22950571540982E-17 |
| rsfmri_c_ngd_smm_ngd_dt | -7.558476992 | 2.81640271420573E-13 | -8.215152631 | 1.60334325244991E-15 |
| rsfmri_c_ngd_smm_ngd_fo | -13.3762499 | 1.97461139556729E-38 | -15.87374972 | 7.55857983529618E-53 |
| rsfmri_c_ngd_smm_ngd_rspltp | 4.04280326892946 | 0.000135007610716 | 7.01971664368531 | 1.13901717577485E-11 |
| rsfmri_c_ngd_smm_ngd_smh | 37.0972585637301 | 6.70397543070745E-236 | 36.2770100997169 | 2.00219004984975E-225 |
| rsfmri_c_ngd_smm_ngd_smm | 7.55257156055928 | 2.92721189143326E-13 | 6.95862314940543 | 1.72858259228438E-11 |
| rsfmri_c_ngd_smm_ngd_sa | -15.27306949 | 2.89157948825647E-49 | -12.85451594 | 1.24272241952234E-35 |
| rsfmri_c_ngd_smm_ngd_vta | 3.18104980664886 | 0.003029316769775 | 3.13686065310527 | 0.0032237845306 |
| rsfmri_c_ngd_smm_ngd_vs | 5.80223436293765 | 2.5906062895755E-08 | 6.82572165603517 | 4.16626779903636E-11 |
| rsfmri_c_ngd_sa_ngd_ad | -18.23430058 | 2.07028402935046E-68 | -18.51338986 | 3.22467098532403E-70 |
| rsfmri_c_ngd_sa_ngd_cgc | -4.102225819 | 0.000106965137287 | -3.771500833 | 0.000350096350079 |
| rsfmri_c_ngd_sa_ngd_ca | 9.06587556851785 | 1.45563781334035E-18 | 6.28833008936878 | 1.24391785744257E-09 |
| rsfmri_c_ngd_sa_ngd_fo | 6.64750795261643 | 1.50268396513163E-10 | 7.16436832396338 | 4.1330661328599E-12 |
| rsfmri_c_ngd_sa_ngd_smh | -18.8951899 | 4.81565233623175E-73 | -17.86095263 | 9.66350810914392E-66 |
| rsfmri_c_ngd_sa_ngd_smm | -15.27306949 | 2.89157948825647E-49 | -12.85451594 | 1.24272241952234E-35 |
| rsfmri_c_ngd_sa_ngd_vta | -11.34504415 | 3.14921891355913E-28 | -11.1044231 | 4.45190239578017E-27 |
| rsfmri_c_ngd_sa_ngd_vs | -4.021524032 | 0.000145593305977 | -3.648383237 | 0.000552640198485 |
| rsfmri_c_ngd_vta_ngd_ca | -4.780194379 | 5.31493955470563E-06 | -6.666008596 | 1.18357367949167E-10 |
| rsfmri_c_ngd_vta_ngd_dt | -7.474361257 | 5.10306559199606E-13 | -8.533059636 | 1.21469329394602E-16 |
| rsfmri_c_ngd_vta_ngd_dla | 7.84742346471843 | 3.17026764596153E-14 | 7.87050487196811 | 2.39612605168255E-14 |
| rsfmri_c_ngd_vta_ngd_fo | -4.62513171 | 1.09152839972059E-05 | -5.348608757 | 2.71664987573368E-07 |
| rsfmri_c_ngd_vta_ngd_n | -3.901245234 | 0.000235435651993 | -4.526239163 | 1.50731748682194E-05 |
| rsfmri_c_ngd_vta_ngd_rspltp | 3.14428522334377 | 0.00338649770742 | 3.1625088960319 | 0.002973556350552 |
| rsfmri_c_ngd_vta_ngd_smh | 5.39052445358394 | 2.48272852336853E-07 | 5.09723818153208 | 9.9699166455301E-07 |
| rsfmri_c_ngd_vta_ngd_smm | 3.18104980664886 | 0.003029316769775 | 3.13686065310527 | 0.0032237845306 |
| rsfmri_c_ngd_vta_ngd_sa | -11.34504415 | 3.14921891355913E-28 | -11.1044231 | 4.45190239578017E-27 |
| rsfmri_c_ngd_vta_ngd_vta | -5.231307558 | 5.65258742238588E-07 | -2.957011565 | 0.005534307668613 |
| rsfmri_c_ngd_vta_ngd_vs | 8.70895144122433 | 3.09103529489477E-17 | 8.41546486791601 | 3.14599871848051E-16 |
| rsfmri_c_ngd_vs_ngd_ad | 18.1548133906315 | 7.09885012609724E-68 | 19.0830909055897 | 3.18089027712073E-74 |
| rsfmri_c_ngd_vs_ngd_cgc | 3.89938329159247 | 0.000235912742039 | 4.85080640160201 | 3.35058473861478E-06 |
| rsfmri_c_ngd_vs_ngd_ca | -4.963515664 | 2.17895800909771E-06 | -4.118762879 | 8.82417628055702E-05 |
| rsfmri_c_ngd_vs_ngd_dla | -7.101471237 | 6.95170164643606E-12 | -6.19421615 | 2.22229520033836E-09 |
| rsfmri_c_ngd_vs_ngd_fo | -5.582617565 | 8.78619014914633E-08 | -5.114841113 | 9.17976552888048E-07 |
| rsfmri_c_ngd_vs_ngd_n | 5.92257101327243 | 1.31084812931926E-08 | 3.08057810683403 | 0.003846443365608 |
| rsfmri_c_ngd_vs_ngd_smh | 20.1464943139387 | 3.627419731637E-82 | 21.0991500651214 | 4.70843346566054E-89 |
| rsfmri_c_ngd_vs_ngd_smm | 5.80223436293765 | 2.5906062895755E-08 | 6.82572165603517 | 4.16626779903636E-11 |
| rsfmri_c_ngd_vs_ngd_sa | -4.021524032 | 0.000145593305977 | -3.648383237 | 0.000552640198485 |
| rsfmri_c_ngd_vs_ngd_vta | 8.70895144122433 | 3.09103529489477E-17 | 8.41546486791601 | 3.14599871848051E-16 |
| rsfmri_c_ngd_vs_ngd_vs | -6.022377049 | 7.36044850323915E-09 | -6.025091998 | 6.07428057212335E-09 |
| rsfmri_var_cdk_cdmdflh | 2.76788938200421 | 0.010303938893806 | 5.71249356490546 | 3.70188054562247E-08 |
| rsfmri_var_cdk_iftlh | -5.371045124 | 2.7524860945208E-07 | -4.786178858 | 4.49537399612488E-06 |
| rsfmri_var_cdk_loboflh | -3.885954666 | 0.000247287977588 | -3.113459532 | 0.003474297351945 |
| rsfmri_var_cdk_moboflh | -6.085163584 | 5.03494135728486E-09 | -4.913844194 | 2.46385454744171E-06 |
| rsfmri_var_cdk_mdtlh | -3.83190872 | 0.000303708987886 | -3.788883569 | 0.000329896347503 |
| rsfmri_var_cdk_paracentrallh | 25.7181287679193 | 2.278620858847E-127 | 27.4927341169901 | 3.85348306576308E-142 |
| rsfmri_var_cdk_parsobalislh | -2.84656998 | 0.008271409204472 | -2.806376716 | 0.008624199159893 |
| rsfmri_var_cdk_pericclh | 6.43793933301023 | 5.70958001676821E-10 | 9.93510229580777 | 5.28091830447711E-22 |
| rsfmri_var_cdk_postcentrallh | 14.2806562502533 | 2.0038568705875E-43 | 18.8282871899419 | 2.04804212072324E-72 |
| rsfmri_var_cdk_psclatelh | 3.65889143565275 | 0.000580341788963 | 6.3744301486375 | 7.41414964097745E-10 |
| rsfmri_var_cdk_precentrallh | 7.37336332864847 | 1.05817079532109E-12 | 11.0073561858646 | 1.21188632967548E-26 |
| rsfmri_var_cdk_rlaclatelh | -4.076711995 | 0.000117906658314 | -3.849296728 | 0.000260601306348 |
| rsfmri_var_cdk_tpolelh | -3.067391437 | 0.00430610911974 | -3.932221642 | 0.00018794554204 |
| rsfmri_var_cdk_cdmdfrh | 3.93484134524525 | 0.000207331380787 | 4.47019427816003 | 1.94093595040639E-05 |
| rsfmri_var_cdk_iftrh | -5.522123241 | 1.21282312183236E-07 | -5.170688819 | 6.95212096891955E-07 |
| rsfmri_var_cdk_lobofrh | -4.52838613 | 1.70384307658824E-05 | -3.090590362 | 0.003735793698848 |
| rsfmri_var_cdk_mobofrh | -5.818551697 | 2.37272123220314E-08 | -4.817364344 | 3.89855842057462E-06 |
| rsfmri_var_cdk_mdtrh | -4.21712464 | 6.60957282011203E-05 | -3.829009823 | 0.000282184349866 |
| rsfmri_var_cdk_paracentralrh | 24.7753749281038 | 3.2726571110712E-119 | 25.1124952233547 | 1.91432352574735E-121 |
| rsfmri_var_cdk_periccrh | 7.19781478988479 | 3.66317968794866E-12 | 10.4665167045746 | 2.99489414852874E-24 |
| rsfmri_var_cdk_postcentralrh | 17.3166610350587 | 3.23614894584211E-62 | 22.2415201896781 | 6.55595065260525E-98 |
| rsfmri_var_cdk_psclaterh | 3.98897650297465 | 0.000165979408704 | 6.52309577505387 | 2.91000645179193E-10 |
| rsfmri_var_cdk_precentralrh | 9.49378236096976 | 3.23721340100657E-20 | 12.184564022706 | 3.09459871941817E-32 |
| rsfmri_var_cdk_rlaclaterh | -4.653970869 | 9.59927103976367E-06 | -4.345228409 | 3.36369698439589E-05 |
| rsfmri_var_cdk_tpolerh | -3.230325671 | 0.00258547281611 | -4.717169513 | 6.24838322716938E-06 |
| rsfmri_var_cortgordon_gp10lh | 4.69759446218936 | 7.80143290741091E-06 | 11.1405393918666 | 3.11194747337883E-27 |
| rsfmri_var_cortgordon_gp11lh | -2.803815757 | 0.009325768852142 | -2.75905777 | 0.009893751683401 |
| rsfmri_var_cortgordon_gp12lh | 3.34683838864489 | 0.001737651158826 | 6.46358937718635 | 4.23790817496157E-10 |
| rsfmri_var_cortgordon_gp15lh | 6.30520133448335 | 1.31330853094719E-09 | 9.90946006288563 | 6.70664691318729E-22 |
| rsfmri_var_cortgordon_gp21lh | 2.81853449476428 | 0.008968083819658 | 6.00135732361309 | 6.94068088486089E-09 |
| rsfmri_var_cortgordon_gp30lh | 4.41222866683312 | 2.86453383073666E-05 | 9.82444475485441 | 1.48974565253254E-21 |
| rsfmri_var_cortgordon_gp31lh | 13.8228468286366 | 7.30629601027617E-41 | 20.6571395177046 | 1.00115358278929E-85 |
| rsfmri_var_cortgordon_gp32lh | 12.0372061731404 | 1.51566186763799E-31 | 15.3741748517163 | 8.85196121118496E-50 |
| rsfmri_var_cortgordon_gp35lh | 15.4559662197658 | 2.34303751218736E-50 | 20.4529890607413 | 3.24314716329939E-84 |
| rsfmri_var_cortgordon_gp36lh | 19.4078490072953 | 1.04048296572549E-76 | 23.7728343488034 | 3.08022400196612E-110 |
| rsfmri_var_cortgordon_gp37lh | 6.63376236058864 | 1.63892049672814E-10 | 9.58511034547365 | 1.3059565777686E-20 |
| rsfmri_var_cortgordon_gp46lh | 8.92976105422782 | 4.73114703950997E-18 | 8.57487027691081 | 8.69581538070181E-17 |
| rsfmri_var_cortgordon_gp57lh | 13.3531423921097 | 2.607078400186E-38 | 15.1163912937279 | 3.13932455740819E-48 |
| rsfmri_var_cortgordon_gp59lh | 2.92194776666589 | 0.006631858707814 | 8.60396646787779 | 6.84322447504441E-17 |
| rsfmri_var_cortgordon_gp65lh | 3.92664746572518 | 0.000213257611016 | 10.839865330179 | 6.71463056342159E-26 |
| rsfmri_var_cortgordon_gp66lh | 3.11283182707845 | 0.003749679656773 | 7.87047691714934 | 2.39612605168255E-14 |
| rsfmri_var_cortgordon_gp68lh | 5.76668241443657 | 3.13969961074119E-08 | 4.75437669932715 | 5.24066861241021E-06 |
| rsfmri_var_cortgordon_gp69lh | 3.02169583934684 | 0.004896019769963 | 8.48693485940722 | 1.76637805241151E-16 |
| rsfmri_var_cortgordon_gp77lh | 3.07920507519981 | 0.004149233333565 | 6.59950729024036 | 1.78133763318773E-10 |
| rsfmri_var_cortgordon_gp80lh | -5.746312776 | 3.50793502067151E-08 | -5.41432304 | 1.92589862851608E-07 |
| rsfmri_var_cortgordon_gp90lh | 3.41492358575918 | 0.001371414601891 | 7.81360050245364 | 3.68638616154675E-14 |
| rsfmri_var_cortgordon_gp99lh | 11.3534868152676 | 2.92432712898257E-28 | 16.5604449584791 | 3.64968935731758E-57 |
| rsfmri_var_cortgordon_gp117lh | -5.126594169 | 9.63647976198887E-07 | -3.704495592 | 0.000450662729707 |
| rsfmri_var_cortgordon_gp123lh | -4.405117269 | 2.95004914468402E-05 | -3.968583512 | 0.000162868124027 |
| rsfmri_var_cortgordon_gp124lh | -3.768386975 | 0.000384471735262 | -3.278013038 | 0.00203599006415 |
| rsfmri_var_cortgordon_gp125lh | -2.997915339 | 0.005281181372809 | -4.118902289 | 8.82417628055702E-05 |
| rsfmri_var_cortgordon_gp128lh | -4.367179401 | 3.4742016398453E-05 | -4.370687039 | 3.02200289543468E-05 |
| rsfmri_var_cortgordon_gp129lh | -2.738606286 | 0.01121707700603 | -4.408945671 | 2.55225540751406E-05 |
| rsfmri_var_cortgordon_gp150lh | -3.525297465 | 0.0009391969843 | -3.30426201 | 0.001859623258212 |
| rsfmri_var_cortgordon_gp171rh | 6.23920915403407 | 1.96636108354184E-09 | 6.23071483924901 | 1.78182394203325E-09 |
| rsfmri_var_cortgordon_gp173rh | 4.21714069234015 | 6.60957282011203E-05 | 5.7591331115353 | 2.84195291679773E-08 |
| rsfmri_var_cortgordon_gp175rh | 5.50026851904648 | 1.36059059178488E-07 | 8.53176367822313 | 1.22113326549207E-16 |
| rsfmri_var_cortgordon_gp180rh | 4.63019698454538 | 1.07238051107311E-05 | 8.1180925624764 | 3.48360993360871E-15 |
| rsfmri_var_cortgordon_gp182rh | 3.48960168707759 | 0.001059716093591 | 3.66216617839599 | 0.000527711797766 |
| rsfmri_var_cortgordon_gp184rh | -3.933099791 | 0.000208236886431 | -2.63147052 | 0.014017489919381 |
| rsfmri_var_cortgordon_gp190rh | 12.3439439681585 | 4.54681841839683E-33 | 20.6393399919141 | 1.33296680199034E-85 |
| rsfmri_var_cortgordon_gp191rh | 16.7647657428938 | 1.28269048324458E-58 | 20.3670663504313 | 1.40185807423801E-83 |
| rsfmri_var_cortgordon_gp193rh | 15.3584241523872 | 9.11938251335265E-50 | 16.1801965779255 | 9.37033792208049E-55 |
| rsfmri_var_cortgordon_gp194rh | 9.06684441598659 | 1.45563781334035E-18 | 7.50204330686872 | 3.7848381151585E-13 |
| rsfmri_var_cortgordon_gp195rh | 5.70324308081464 | 4.46957167578623E-08 | 7.75707779624792 | 5.64005344702682E-14 |
| rsfmri_var_cortgordon_gp201rh | 4.06117441940295 | 0.000125609143343 | 6.07733824968293 | 4.49594749863962E-09 |
| rsfmri_var_cortgordon_gp202rh | 12.3545535672871 | 4.05597628210699E-33 | 13.3491634641962 | 3.18290221900643E-38 |
| rsfmri_var_cortgordon_gp204rh | 6.99979658822542 | 1.39421133453734E-11 | 11.0879782189045 | 5.21162293417331E-27 |
| rsfmri_var_cortgordon_gp206rh | 3.72085804111834 | 0.00045792657337 | 4.612660438 | 1.01603240335486E-05 |
| rsfmri_var_cortgordon_gp207rh | 3.18373656109631 | 0.003015924793346 | 3.53003406559102 | 0.000843512051333 |
| rsfmri_var_cortgordon_gp214rh | 17.2811553260285 | 5.49938816055458E-62 | 17.5775700605632 | 7.9598241095264E-64 |
| rsfmri_var_cortgordon_gp215rh | 2.84288361974639 | 0.00834912289221 | 7.32862327769452 | 1.3252244220697E-12 |
| rsfmri_var_cortgordon_gp216rh | 3.44181608481813 | 0.001248880964927 | 9.40457258685454 | 6.78100712209964E-20 |
| rsfmri_var_cortgordon_gp218rh | 4.27188539318379 | 5.25064537564265E-05 | 7.66262884577748 | 1.13694946825742E-13 |
| rsfmri_var_cortgordon_gp224rh | 7.0375803734098 | 1.07503659864914E-11 | 14.9416961565715 | 3.41382741747023E-47 |
| rsfmri_var_cortgordon_gp227rh | 2.78154395739095 | 0.009902780754062 | 10.0483197057072 | 1.79408762525605E-22 |
| rsfmri_var_cortgordon_gp239rh | 4.30034289521415 | 4.66711086692342E-05 | 9.27716910989073 | 2.11971743187407E-19 |
| rsfmri_var_cortgordon_gp242rh | -2.996167659 | 0.005287504925266 | -3.208237294 | 0.002569039747759 |
| rsfmri_var_cortgordon_gp243rh | -4.08876939 | 0.000112499918524 | -2.737625963 | 0.01047634056003 |
| rsfmri_var_cortgordon_gp250rh | -4.117839392 | 0.000100920191384 | -2.636466915 | 0.013852095923482 |
| rsfmri_var_cortgordon_gp255rh | 3.33281194691874 | 0.001822743375832 | 9.81312577184622 | 1.64732056408693E-21 |
| rsfmri_var_cortgordon_gp264rh | 12.2267486935364 | 1.74384669507825E-32 | 20.0184987330735 | 5.45162209578065E-81 |
| rsfmri_var_cortgordon_gp267rh | 2.6934466880014 | 0.012742718936212 | 9.70030339999801 | 4.59382229890805E-21 |
| rsfmri_var_cortgordon_gp274rh | -4.282568793 | 5.03742404870739E-05 | -2.919534705 | 0.006191217240229 |
| rsfmri_var_cortgordon_gp279rh | -3.43642734 | 0.00127068833528 | -2.672504014 | 0.012538941605751 |
| rsfmri_var_cortgordon_gp287rh | -4.517675567 | 1.78580612584367E-05 | -4.808360953 | 4.05244616585763E-06 |
| rsfmri_var_cortgordon_gp288rh | -2.80910336 | 0.009214182130273 | -5.050372914 | 1.25626550302206E-06 |
| rsfmri_var_cortgordon_gp290rh | -2.639002383 | 0.014758242035418 | -3.570353636 | 0.000729463402244 |
| rsfmri_var_cortgordon_gp292rh | -3.050987271 | 0.004495356944589 | -4.056437894 | 0.000114001953587 |
| rsfmri_var_cortgordon_gp297rh | -2.635017044 | 0.014901220899826 | -2.751591225 | 0.010101249137489 |
| rsfmri_var_cortgordon_gp301rh | -2.621564542 | 0.015436656790206 | -2.665457996 | 0.012779046717233 |
| rsfmri_var_cortgordon_gp302rh | -3.128613416 | 0.003563197813246 | -3.646130532 | 0.000556136286276 |
| rsfmri_var_cortgordon_gp311rh | 3.64638742790752 | 0.000607542264995 | 9.27470655800638 | 2.15371734391849E-19 |
| rsfmri_var_cortgordon_gp322rh | -3.442846226 | 0.001247275337129 | -2.970600046 | 0.005318231754327 |
| rsfmri_var_scs_cbwmlh | -4.897001282 | 3.00045540345025E-06 | -3.948742774 | 0.000176418834785 |
| rsfmri_var_scs_cbcortexlh | -6.448560873 | 5.37942518191137E-10 | -5.755593376 | 2.89120755337902E-08 |
| rsfmri_var_scs_crbcortexlh | -2.729155535 | 0.011493833356904 | -3.03233901 | 0.004476182921739 |
| rsfmri_var_scs_tplh | 12.5923098037913 | 2.53406134699505E-34 | 12.5390864558055 | 5.25016835892404E-34 |
| rsfmri_var_scs_caudatelh | 5.3251112089729 | 3.5103781124097E-07 | 6.90030693532444 | 2.57656039317058E-11 |
| rsfmri_var_scs_brainstem | -2.787668745 | 0.009738914752249 | -3.050607204 | 0.004222749011574 |
| rsfmri_var_scs_hpuslh | -3.398848123 | 0.001450586211285 | -3.063895663 | 0.004049070837812 |
| rsfmri_var_scs_csf | 8.41678913876424 | 3.50363469553613E-16 | 5.6296689812831 | 5.8818785468398E-08 |
| rsfmri_var_scs_cbwmrh | -5.074622768 | 1.25256381256429E-06 | -3.107614452 | 0.003535724260745 |
| rsfmri_var_scs_cbcortexrh | -5.222478264 | 5.88297254943902E-07 | -4.272596791 | 4.63345865876736E-05 |
| rsfmri_var_scs_crbwmrh | -3.204346882 | 0.002815753688261 | -2.628894716 | 0.014096615666784 |
| rsfmri_var_scs_crbcortexrh | -3.175248159 | 0.003075533657936 | -3.561479692 | 0.000752654316794 |
| rsfmri_var_scs_tprh | 12.2011797433471 | 2.32236911247522E-32 | 14.7511953982951 | 4.38429715313013E-46 |
| rsfmri_var_scs_caudaterh | 5.15163198525855 | 8.47688648255117E-07 | 7.24950696800951 | 2.28566162729019E-12 |
| rsfmri_cor_ngd_au_scs_crcxlh | -18.46944704 | 4.97602854562131E-70 | -21.16894657 | 1.44589275149695E-89 |
| rsfmri_cor_ngd_au_scs_thplh | -3.391498084 | 0.001486177237065 | -3.57573127 | 0.000716401946355 |
| rsfmri_cor_ngd_au_scs_cdelh | 7.67635849800128 | 1.16840592377761E-13 | 8.79276121925799 | 1.39998043396627E-17 |
| rsfmri_cor_ngd_au_scs_ptlh | 13.6603396000751 | 5.78517553503533E-40 | 16.0726213481778 | 4.41184623551414E-54 |
| rsfmri_cor_ngd_au_scs_bs | 13.5480946936761 | 2.37779553925769E-39 | 13.5912332253497 | 1.56174198771178E-39 |
| rsfmri_cor_ngd_au_scs_aglh | -3.165950742 | 0.003167562446496 | -3.201175805 | 0.00262668576065 |
| rsfmri_cor_ngd_au_scs_aalh | -24.42313046 | 3.18551777527115E-116 | -24.60667443 | 3.66563689189692E-117 |
| rsfmri_cor_ngd_au_scs_vtdclh | -19.37425251 | 1.78328860261667E-76 | -20.68693785 | 6.10105149511471E-86 |
| rsfmri_cor_ngd_au_scs_crcxrh | 9.88692607900618 | 8.53234618276167E-22 | 10.9979335826589 | 1.31639230888235E-26 |
| rsfmri_cor_ngd_au_scs_cderh | -11.10199197 | 4.18273659612256E-27 | -13.08872072 | 7.51938765844797E-37 |
| rsfmri_cor_ngd_au_scs_ptrh | -30.00436431 | 3.20021074970269E-166 | -28.96989924 | 1.6719639522236E-155 |
| rsfmri_cor_ngd_au_scs_plrh | -13.46238945 | 6.85170476859574E-39 | -13.33914579 | 3.56636214762185E-38 |
| rsfmri_cor_ngd_au_scs_hprh | 13.1719700159703 | 2.44969216380743E-37 | 12.4544737838529 | 1.407441743309E-33 |
| rsfmri_cor_ngd_au_scs_agrh | 11.58548421 | 2.38725849724736E-29 | 9.39013876151084 | 7.63887217919105E-20 |
| rsfmri_cor_ngd_au_scs_vtdcrh | 15.8829859817203 | 5.5225415431983E-53 | 16.8738895222269 | 3.52528898718696E-59 |
| rsfmri_cor_ngd_cerc_scs_thplh | -3.541666576 | 0.000887639370411 | -6.322467376 | 1.02111609438011E-09 |
| rsfmri_cor_ngd_cerc_scs_cdelh | -29.46266137 | 3.24797905827591E-161 | -28.10947823 | 1.12796619003948E-147 |
| rsfmri_cor_ngd_cerc_scs_ptlh | -27.7808029 | 9.65316355775746E-146 | -27.04751253 | 3.42753615779881E-138 |
| rsfmri_cor_ngd_cerc_scs_pllh | 8.47079788205823 | 2.25056007779274E-16 | 9.63964272042871 | 8.00552660967949E-21 |
| rsfmri_cor_ngd_cerc_scs_bs | -13.09884423 | 5.90318698999406E-37 | -14.66872129 | 1.28682648151934E-45 |
| rsfmri_cor_ngd_cerc_scs_hplh | -9.512833459 | 2.73386907623109E-20 | -9.710217304 | 4.21321141201548E-21 |
| rsfmri_cor_ngd_cerc_scs_aglh | -28.9889053 | 7.72320585145101E-157 | -31.32767019 | 2.13398062768925E-177 |
| rsfmri_cor_ngd_cerc_scs_aalh | -9.99282059 | 3.09803195298528E-22 | -13.97074113 | 1.24703884913881E-41 |
| rsfmri_cor_ngd_cerc_scs_vtdclh | 15.6431728811738 | 1.6705325480037E-51 | 12.9284291485408 | 5.18982856078943E-36 |
| rsfmri_cor_ngd_cerc_scs_crcxrh | 6.47891724722752 | 4.43695987846297E-10 | 4.91956340004594 | 2.40095600781613E-06 |
| rsfmri_cor_ngd_cerc_scs_cderh | 13.11232037 | 5.05578543981807E-37 | 9.8338039491622 | 1.37256030038442E-21 |
| rsfmri_cor_ngd_cerc_scs_hprh | -35.04227009 | 2.60725777606862E-215 | -34.71399185 | 5.08604608964313E-210 |
| rsfmri_cor_ngd_cerc_scs_agrh | -23.91478204 | 5.76441165888523E-112 | -23.58968093 | 8.95206406841524E-109 |
| rsfmri_cor_ngd_cerc_scs_aarh | 6.86027231794059 | 3.64848792671146E-11 | 4.10987445322675 | 9.14411512666923E-05 |
| rsfmri_cor_ngd_cerc_scs_vtdcrh | -15.30036923 | 2.02923938266333E-49 | -17.01327561 | 4.43039482814054E-60 |
| rsfmri_cor_ngd_copa_scs_crcxlh | -5.562789096 | 9.79300427745839E-08 | -4.653457971 | 8.42978627470844E-06 |
| rsfmri_cor_ngd_copa_scs_thplh | -23.09253791 | 3.61541690129034E-105 | -26.81068987 | 4.06658223133665E-136 |
| rsfmri_cor_ngd_copa_scs_cdelh | -9.19119507 | 5.03198856706833E-19 | -11.18420266 | 1.97048546054404E-27 |
| rsfmri_cor_ngd_copa_scs_ptlh | 9.04532004318698 | 1.72284372827118E-18 | 7.49821365296111 | 3.87502937647434E-13 |
| rsfmri_cor_ngd_copa_scs_hplh | 7.1073042063247 | 6.73809948831195E-12 | 3.35621436651529 | 0.001555041133891 |
| rsfmri_cor_ngd_copa_scs_vtdclh | -22.18171269 | 7.15615246477809E-98 | -21.85068669 | 7.59054908874389E-95 |
| rsfmri_cor_ngd_copa_scs_crcxrh | -18.24532045 | 1.81316139389258E-68 | -18.4047459 | 1.82276604011314E-69 |
| rsfmri_cor_ngd_copa_scs_cderh | -11.19142677 | 1.6367343628424E-27 | -13.20172832 | 1.91513382245632E-37 |
| rsfmri_cor_ngd_copa_scs_plrh | -20.63208732 | 8.36251437532855E-86 | -21.72244408 | 7.4771265190201E-94 |
| rsfmri_cor_ngd_copa_scs_hprh | -7.862896686 | 2.86125416973118E-14 | -10.20967283 | 3.81322046427162E-23 |
| rsfmri_cor_ngd_copa_scs_agrh | 8.54321100194489 | 1.24343927023498E-16 | 6.60849230582536 | 1.68534319516055E-10 |
| rsfmri_cor_ngd_copa_scs_aarh | 6.13172124205393 | 3.82845552587343E-09 | 4.80331195146799 | 4.14278993999397E-06 |
| rsfmri_cor_ngd_df_scs_crcxlh | 7.93300408792514 | 1.67403487039442E-14 | 7.27505423246757 | 1.90928886386166E-12 |
| rsfmri_cor_ngd_df_scs_ptlh | -20.35239115 | 1.07995663137284E-83 | -20.63134605 | 1.49660539603853E-85 |
| rsfmri_cor_ngd_df_scs_pllh | -17.81461798 | 1.52166720195349E-65 | -17.94096483 | 2.83489304615464E-66 |
| rsfmri_cor_ngd_df_scs_bs | 3.58368579970299 | 0.000764835485397 | 5.32899201195339 | 3.01310339086267E-07 |
| rsfmri_cor_ngd_df_scs_hplh | -8.38856177 | 4.39784897763031E-16 | -10.93733157 | 2.4675935599881E-26 |
| rsfmri_cor_ngd_df_scs_aalh | -17.89986529 | 4.03336964558241E-66 | -16.64843859 | 1.01602943657097E-57 |
| rsfmri_cor_ngd_df_scs_vtdclh | -5.984398463 | 9.1491075219818E-09 | -4.932401217 | 2.25678175905844E-06 |
| rsfmri_cor_ngd_df_scs_crcxrh | 5.92364654687967 | 1.31084812931926E-08 | 7.46880289658116 | 4.77420156799029E-13 |
| rsfmri_cor_ngd_df_scs_thprh | 9.16781373907557 | 6.11767514101355E-19 | 5.3048663906384 | 3.42334374236353E-07 |
| rsfmri_cor_ngd_df_scs_ptrh | 8.65431835252135 | 4.89320109938804E-17 | 6.7040379051679 | 9.25297481101836E-11 |
| rsfmri_cor_ngd_df_scs_hprh | -2.800062801 | 0.009414234729138 | -3.600799132 | 0.000657311692784 |
| rsfmri_cor_ngd_df_scs_agrh | -16.55571982 | 2.89758243997319E-57 | -14.4620281 | 2.02361148202146E-44 |
| rsfmri_cor_ngd_df_scs_aarh | -16.92449109 | 1.2031967700478E-59 | -14.67247784 | 1.23808573466264E-45 |
| rsfmri_cor_ngd_df_scs_vtdcrh | 6.55440692763278 | 2.71928766342456E-10 | 5.98207195155431 | 7.74465562000392E-09 |
| rsfmri_cor_ngd_dsa_scs_crcxlh | -5.846948363 | 2.01441675317342E-08 | -8.462274132 | 2.15712127407027E-16 |
| rsfmri_cor_ngd_dsa_scs_thplh | -7.851887098 | 3.09860456053128E-14 | -6.289420465 | 1.24391785744257E-09 |
| rsfmri_cor_ngd_dsa_scs_cdelh | 2.95829788818963 | 0.005953150411467 | 3.31096866936979 | 0.001819949753389 |
| rsfmri_cor_ngd_dsa_scs_ptlh | 5.07418116588715 | 1.25256381256429E-06 | 6.42529535528531 | 5.36603525163391E-10 |
| rsfmri_cor_ngd_dsa_scs_bs | -8.018170411 | 8.6028192286329E-15 | -10.18983999 | 4.60019056206614E-23 |
| rsfmri_cor_ngd_dsa_scs_hplh | 5.98952050274661 | 8.90809855555012E-09 | 6.069296944 | 4.69362836692816E-09 |
| rsfmri_cor_ngd_dsa_scs_aalh | -4.281736589 | 5.04060286784595E-05 | -3.244821557 | 0.002267127267194 |
| rsfmri_cor_ngd_dsa_scs_vtdclh | -3.873826533 | 0.000258300813654 | -3.820358894 | 0.000291470255333 |
| rsfmri_cor_ngd_dsa_scs_thprh | 4.88015125397293 | 3.25470171972462E-06 | 3.68054864705283 | 0.000493688945271 |
| rsfmri_cor_ngd_dsa_scs_plrh | 5.75490203574678 | 3.34991506918106E-08 | 4.609663532 | 1.0277228119407E-05 |
| rsfmri_cor_ngd_dsa_scs_hprh | 5.22533367215913 | 5.8149011703841E-07 | 5.48384786793762 | 1.32261855554208E-07 |
| rsfmri_cor_ngd_dsa_scs_agrh | 3.51469955519664 | 0.000974220627674 | 3.49586595534494 | 0.000947502857364 |
| rsfmri_cor_ngd_dsa_scs_aarh | -4.923741646 | 2.64853898775797E-06 | -3.585276462 | 0.000692481107816 |
| rsfmri_cor_ngd_dsa_scs_vtdcrh | -9.118713118 | 9.42124430736041E-19 | -10.0011842 | 2.81243159299692E-22 |
| rsfmri_cor_ngd_fopa_scs_crcxlh | 4.55426965185825 | 1.513407066927E-05 | 4.24509534968506 | 5.2207162896216E-05 |
| rsfmri_cor_ngd_fopa_scs_cdelh | -6.091293509 | 4.87003486397128E-09 | -4.817615041 | 3.89855842057462E-06 |
| rsfmri_cor_ngd_fopa_scs_ptlh | -5.032971968 | 1.5416940169213E-06 | -6.124823282 | 3.36875601635613E-09 |
| rsfmri_cor_ngd_fopa_scs_pllh | 7.08341511486249 | 7.85997948039546E-12 | 4.66489687166488 | 8.00135157414073E-06 |
| rsfmri_cor_ngd_fopa_scs_bs | 6.24093506464828 | 1.95384944141378E-09 | 5.8179795759025 | 2.02646903617572E-08 |
| rsfmri_cor_ngd_fopa_scs_hplh | -4.39161351 | 3.11821550074911E-05 | -4.728620705 | 5.92657023713149E-06 |
| rsfmri_cor_ngd_fopa_scs_aalh | 6.79873348080866 | 5.50286227923094E-11 | 6.15533267853412 | 2.80967638536845E-09 |
| rsfmri_cor_ngd_fopa_scs_vtdclh | -9.67747036 | 6.10127271451829E-21 | -9.580838495 | 1.35000247118478E-20 |
| rsfmri_cor_ngd_fopa_scs_thprh | 7.82098010884918 | 3.86955411619272E-14 | 6.0463136516217 | 5.37629596031426E-09 |
| rsfmri_cor_ngd_fopa_scs_plrh | 5.55703739993871 | 1.00775013841631E-07 | 3.18226192212771 | 0.002797372651639 |
| rsfmri_cor_ngd_fopa_scs_hprh | -4.744526457 | 6.26899573246612E-06 | -5.062322671 | 1.18450030505587E-06 |
| rsfmri_cor_ngd_fopa_scs_aarh | -14.81572028 | 1.55619084870404E-46 | -11.80668649 | 2.2234806141755E-30 |
| rsfmri_cor_ngd_fopa_scs_vtdcrh | -7.362415775 | 1.13422750515006E-12 | -6.802887129 | 4.8047140656743E-11 |
| rsfmri_cor_ngd_none_scs_thplh | -5.25626512 | 4.99922233516334E-07 | -7.342196785 | 1.20614790735843E-12 |
| rsfmri_cor_ngd_none_scs_ptlh | -22.40259133 | 1.24208061016899E-99 | -21.02573954 | 1.67759560366807E-88 |
| rsfmri_cor_ngd_none_scs_bs | 9.85663141205709 | 1.13170190216444E-21 | 10.1730426471385 | 5.3850387446675E-23 |
| rsfmri_cor_ngd_none_scs_hplh | 5.71197017096694 | 4.26615947633183E-08 | 6.52056113520612 | 2.94676435773322E-10 |
| rsfmri_cor_ngd_none_scs_aalh | 13.0306993298546 | 1.34746996243089E-36 | 13.1237966276305 | 4.94309509366583E-37 |
| rsfmri_cor_ngd_none_scs_crcxrh | -3.081599924 | 0.00412572677541 | -5.160009667 | 7.30859523960535E-07 |
| rsfmri_cor_ngd_none_scs_thprh | -26.81967665 | 4.30462760755668E-137 | -23.9218569 | 1.86048273857689E-111 |
| rsfmri_cor_ngd_none_scs_cderh | -21.48919689 | 2.1419217100313E-92 | -19.35223839 | 3.97121505048112E-76 |
| rsfmri_cor_ngd_none_scs_ptrh | 8.18826353701568 | 2.21834403220044E-15 | 10.6072942604576 | 7.30277647147393E-25 |
| rsfmri_cor_ngd_none_scs_plrh | -9.081445733 | 1.29432833623548E-18 | -9.784447857 | 2.14778282365388E-21 |
| rsfmri_cor_ngd_none_scs_hprh | -6.803383379 | 5.35744851082113E-11 | -9.255376692 | 2.54834660681281E-19 |
| rsfmri_cor_ngd_none_scs_agrh | -16.94263973 | 9.2869679147784E-60 | -16.6440279 | 1.06906743164419E-57 |
| rsfmri_cor_ngd_none_scs_vtdcrh | 7.49347214230194 | 4.47649037063486E-13 | 6.65639891050405 | 1.25665897874333E-10 |
| rsfmri_cor_ngd_rst_scs_crcxlh | 11.020848009174 | 9.81033579528929E-27 | 11.1146261310236 | 4.06407575700771E-27 |
| rsfmri_cor_ngd_rst_scs_cdelh | 13.4676785194888 | 6.48492355032882E-39 | 13.4401233389838 | 1.0336835876126E-38 |
| rsfmri_cor_ngd_rst_scs_bs | -23.15102025 | 1.24576429235105E-105 | -22.0430072 | 2.3830958803191E-96 |
| rsfmri_cor_ngd_rst_scs_hplh | -17.49001993 | 2.28300366546237E-63 | -16.94398743 | 1.24404273730082E-59 |
| rsfmri_cor_ngd_rst_scs_aglh | 9.64353691026001 | 8.32858193215248E-21 | 9.7512054369237 | 2.88131119585182E-21 |
| rsfmri_cor_ngd_rst_scs_vtdclh | -9.110947459 | 1.00253758511186E-18 | -10.24390887 | 2.74090458460658E-23 |
| rsfmri_cor_ngd_rst_scs_crcxrh | -30.66755546 | 2.02777262909905E-172 | -29.24238738 | 5.62813545958378E-158 |
| rsfmri_cor_ngd_rst_scs_thprh | -15.25755809 | 3.54292609768547E-49 | -14.32786737 | 1.18459086521843E-43 |
| rsfmri_cor_ngd_rst_scs_cderh | 13.8314609067491 | 6.61912976707855E-41 | 12.6774861519907 | 1.0271011830231E-34 |
| rsfmri_cor_ngd_rst_scs_ptrh | 14.1284314080213 | 1.43147872935264E-42 | 12.3462072784679 | 4.94263173907364E-33 |
| rsfmri_cor_ngd_rst_scs_plrh | -3.766793589 | 0.00038588089337 | -5.897084086 | 1.27664639934148E-08 |
| rsfmri_cor_ngd_rst_scs_hprh | 16.8760007720256 | 2.42939350825604E-59 | 13.7514158095247 | 2.06471979654678E-40 |
| rsfmri_cor_ngd_rst_scs_aarh | -4.11893858 | 0.000100745972575 | -4.490617929 | 1.77517731542874E-05 |
| rsfmri_cor_ngd_rst_scs_vtdcrh | -28.44703112 | 7.75230994872208E-152 | -26.62349647 | 1.72846042635597E-134 |
| rsfmri_cor_ngd_smh_scs_crcxlh | -27.37611509 | 4.36997055282703E-142 | -26.17864016 | 1.32828701945428E-130 |
| rsfmri_cor_ngd_smh_scs_thplh | 10.1317145391009 | 8.06311923545546E-23 | 9.65612730860782 | 6.90919019587866E-21 |
| rsfmri_cor_ngd_smh_scs_cdelh | -10.88469227 | 4.03957979624413E-26 | -11.08909757 | 5.19376628641494E-27 |
| rsfmri_cor_ngd_smh_scs_ptlh | -12.76869174 | 3.17113622256082E-35 | -10.89944099 | 3.63837117249173E-26 |
| rsfmri_cor_ngd_smh_scs_pllh | -30.47406124 | 1.2337545627152E-170 | -33.3420934 | 1.16347100046415E-196 |
| rsfmri_cor_ngd_smh_scs_bs | -11.13826514 | 2.86571904883405E-27 | -14.68126393 | 1.1132965184478E-45 |
| rsfmri_cor_ngd_smh_scs_hplh | 15.7090414097633 | 6.61984745620407E-52 | 13.0294412042536 | 1.53567513019563E-36 |
| rsfmri_cor_ngd_smh_scs_aglh | 7.17523004277123 | 4.26165845410782E-12 | 6.82636448354236 | 4.16626779903636E-11 |
| rsfmri_cor_ngd_smh_scs_vtdclh | 12.2496178001877 | 1.35051389984228E-32 | 10.1418192845792 | 7.26000327456067E-23 |
| rsfmri_cor_ngd_smh_scs_cderh | -36.10585617 | 5.92470381194082E-226 | -36.47106798 | 6.8756192497421E-227 |
| rsfmri_cor_ngd_smh_scs_ptrh | -24.16972242 | 4.28386747279825E-114 | -25.68129957 | 2.54990227541647E-126 |
| rsfmri_cor_ngd_smh_scs_plrh | 5.89711014938792 | 1.50731613348653E-08 | 4.98115487868512 | 1.76623626186392E-06 |
| rsfmri_cor_ngd_smh_scs_hprh | -14.77742755 | 2.5872533877856E-46 | -16.29506548 | 1.77426342410019E-55 |
| rsfmri_cor_ngd_smh_scs_agrh | -5.865806407 | 1.80870449627827E-08 | -5.70647242 | 3.8200479478419E-08 |
| rsfmri_cor_ngd_smh_scs_aarh | -22.74649276 | 2.26401668473872E-102 | -25.9499025 | 1.24801858278455E-128 |
| rsfmri_cor_ngd_smh_scs_vtdcrh | -9.621604075 | 1.00753907298225E-20 | -10.58243792 | 9.33282636409113E-25 |
| rsfmri_cor_ngd_smm_scs_crcxlh | 8.33378899858015 | 6.86257575757306E-16 | 7.8291104222226 | 3.28643884841751E-14 |
| rsfmri_cor_ngd_smm_scs_ptlh | 6.67551314327644 | 1.25833131999932E-10 | 3.67403604584712 | 0.000505142513915 |
| rsfmri_cor_ngd_smm_scs_hplh | -21.11368214 | 1.70524254412089E-89 | -20.70271267 | 4.75077677083923E-86 |
| rsfmri_cor_ngd_smm_scs_aglh | -17.41570013 | 7.11323724946634E-63 | -17.94662578 | 2.63745684618029E-66 |
| rsfmri_cor_ngd_smm_scs_vtdclh | -10.96740804 | 1.70984022610828E-26 | -11.89287146 | 8.51658737680851E-31 |
| rsfmri_cor_ngd_smm_scs_thprh | -19.23555621 | 1.77623188298917E-75 | -18.80873608 | 2.76639340573273E-72 |
| rsfmri_cor_ngd_smm_scs_cderh | -8.501629126 | 1.75080230402886E-16 | -7.963961339 | 1.17754514668532E-14 |
| rsfmri_cor_ngd_smm_scs_ptrh | 5.99785570739638 | 8.50602962004034E-09 | 7.49468633588113 | 3.95849955246684E-13 |
| rsfmri_cor_ngd_smm_scs_plrh | 6.44711580414115 | 5.40492600313056E-10 | 6.49455653525136 | 3.47841889733892E-10 |
| rsfmri_cor_ngd_smm_scs_agrh | 7.89856186981598 | 2.1793115580271E-14 | 6.85322538802304 | 3.49786247306679E-11 |
| rsfmri_cor_ngd_sa_scs_crcxlh | -17.63121437 | 2.56624601555486E-64 | -16.46397743 | 1.50019630109683E-56 |
| rsfmri_cor_ngd_sa_scs_thplh | -16.91662595 | 1.33380739341558E-59 | -17.09468906 | 1.30824252537256E-60 |
| rsfmri_cor_ngd_sa_scs_cdelh | 3.81081258207194 | 0.000327080965334 | 6.01020394763169 | 6.62558560813425E-09 |
| rsfmri_cor_ngd_sa_scs_ptlh | -7.365052267 | 1.11856679090735E-12 | -7.305078478 | 1.54296477458062E-12 |
| rsfmri_cor_ngd_sa_scs_pllh | -3.470623471 | 0.001134228750005 | -4.145793302 | 7.9142680074829E-05 |
| rsfmri_cor_ngd_sa_scs_bs | 3.73025295898151 | 0.000442452273013 | 4.17685715421175 | 6.95157668948085E-05 |
| rsfmri_cor_ngd_sa_scs_hplh | 5.33295573880744 | 3.37634968342136E-07 | 6.06911572575791 | 4.69362836692816E-09 |
| rsfmri_cor_ngd_sa_scs_aalh | -8.891284142 | 6.57602818934824E-18 | -11.00053233 | 1.29144391476011E-26 |
| rsfmri_cor_ngd_sa_scs_vtdclh | 6.30066075852969 | 1.34563265552982E-09 | 4.00186809699372 | 0.00014206872288 |
| rsfmri_cor_ngd_sa_scs_thprh | -4.714687127 | 7.20359744354016E-06 | -3.521642195 | 0.00086848257681 |
| rsfmri_cor_ngd_sa_scs_cderh | -4.246142882 | 5.85043842235652E-05 | -4.212786277 | 5.95571324155273E-05 |
| rsfmri_cor_ngd_sa_scs_plrh | 5.32215271762851 | 3.55397682976625E-07 | 6.00672864016611 | 6.74242047701255E-09 |
| rsfmri_cor_ngd_sa_scs_aarh | 5.64527772434934 | 6.21770959621128E-08 | 5.83411120348304 | 1.84895340125023E-08 |
| rsfmri_cor_ngd_sa_scs_vtdcrh | 8.301986241 | 8.85185006991929E-16 | 8.80757810460684 | 1.23940096338459E-17 |
| rsfmri_cor_ngd_vta_scs_crcxlh | 3.44431881068035 | 0.001245680887556 | 4.68330490751221 | 7.34242243663598E-06 |
| rsfmri_cor_ngd_vta_scs_thplh | -5.77757694 | 2.95765754871008E-08 | -3.927539427 | 0.000191128340803 |
| rsfmri_cor_ngd_vta_scs_cdelh | -11.85019772 | 1.25462491484083E-30 | -11.65890022 | 1.14379871160334E-29 |
| rsfmri_cor_ngd_vta_scs_ptlh | 4.93103938685896 | 2.56123057922953E-06 | 5.02946197256401 | 1.39528433426618E-06 |
| rsfmri_cor_ngd_vta_scs_pllh | -4.56593774 | 1.44159465493269E-05 | -3.595357562 | 0.00066952794905 |
| rsfmri_cor_ngd_vta_scs_bs | -7.074516975 | 8.32829557561698E-12 | -5.950256059 | 9.34952281081881E-09 |
| rsfmri_cor_ngd_vta_scs_hplh | -5.304622699 | 3.89410764887232E-07 | -5.363707607 | 2.5180794150558E-07 |
| rsfmri_cor_ngd_vta_scs_aglh | 11.482858948361 | 7.1911299074497E-29 | 9.15616066682011 | 6.02104105024232E-19 |
| rsfmri_cor_ngd_vta_scs_aalh | 9.8283734939042 | 1.47106366301344E-21 | 9.39573760576484 | 7.30449124715791E-20 |
| rsfmri_cor_ngd_vta_scs_vtdclh | -6.592956515 | 2.11832815816664E-10 | -6.306817301 | 1.1235165247275E-09 |
| rsfmri_cor_ngd_vta_scs_thprh | 9.06125422540527 | 1.50621467514511E-18 | 7.32748074391743 | 1.32984990503585E-12 |
| rsfmri_cor_ngd_vta_scs_cderh | -9.640655517 | 8.49193757358849E-21 | -7.730189051 | 6.89613815804508E-14 |
| rsfmri_cor_ngd_vta_scs_plrh | 6.43246108851549 | 5.88851375956758E-10 | 5.15053861296373 | 7.6584744017838E-07 |
| rsfmri_cor_ngd_vta_scs_aarh | 6.09733015575055 | 4.71316262096257E-09 | 2.71089487976336 | 0.011311852698103 |
| rsfmri_cor_ngd_vta_scs_vtdcrh | -4.473046765 | 2.19166650969042E-05 | -4.624224831 | 9.66975044887742E-06 |
| rsfmri_cor_ngd_vs_scs_thplh | -13.01609522 | 1.59412848177442E-36 | -8.765227663 | 1.76367187511763E-17 |
| rsfmri_cor_ngd_vs_scs_cdelh | -7.331234098 | 1.41710650892623E-12 | -5.100812602 | 9.8474589524216E-07 |
| rsfmri_cor_ngd_vs_scs_pllh | -5.778753562 | 2.94932191659055E-08 | -6.133496447 | 3.20478540985903E-09 |
| rsfmri_cor_ngd_vs_scs_hplh | -22.94659395 | 5.4691902154118E-104 | -22.52886742 | 3.43679624030242E-100 |
| rsfmri_cor_ngd_vs_scs_aglh | -5.28540104 | 4.30477337935824E-07 | -3.008952471 | 0.004732510062603 |
| rsfmri_cor_ngd_vs_scs_aalh | 10.5231599252537 | 1.63504621926667E-24 | 8.94480947896326 | 3.8306663037584E-18 |
| rsfmri_cor_ngd_vs_scs_vtdclh | 7.1939638811208 | 3.74598703928676E-12 | 6.55302856316333 | 2.40081883492638E-10 |
| rsfmri_cor_ngd_vs_scs_thprh | 14.0167973021006 | 6.07883987183806E-42 | 13.2096696126634 | 1.75507534806314E-37 |
| rsfmri_cor_ngd_vs_scs_ptrh | -3.829023145 | 0.000304764823738 | -5.472585465 | 1.39893206587115E-07 |
| rsfmri_cor_ngd_vs_scs_plrh | -25.75453273 | 1.1563833211163E-127 | -24.21734671 | 6.63306485755307E-114 |
| rsfmri_cor_ngd_vs_scs_hprh | -21.4141906 | 8.02932903638174E-92 | -19.70237373 | 1.14170323206429E-78 |
| rsfmri_cor_ngd_vs_scs_agrh | 9.18055219314644 | 5.49661966834705E-19 | 11.5087292389914 | 5.93166688071793E-29 |
| rsfmri_cor_ngd_vs_scs_aarh | -8.808626102 | 1.32953860133497E-17 | -9.164575663 | 5.61905154279706E-19 |
| rsfmri_cor_ngd_vs_scs_vtdcrh | -9.468394015 | 4.06305025702163E-20 | -9.404961974 | 6.78100712209964E-20 |

**Table S2. Different patterns of rs fMRI features for small cluster compared to large cluster from two independent samples.**

| region | t_values_sub1_baseline | p_fdr_ sub1_baseline | t_values_ sub2_baseline | p_fdr_ sub2_baseline | t_values_ sub1_development | p_fdr_sub1_development | t_values_sub2_development | p_fdr_sub2_development | var_label |
| --- | --- | --- | --- | --- | --- | --- | --- | --- | --- |
| Baseline > 0, Development > 0 | | | | | | | | | |
| rsfmri_var_cortgordon_gp227rh | 2.78154395739095 | 0.009902780754062 | 10.0483197057072 | 1.79408762525605e-22 | 5.25299723110815 | 1.44152819269858e-06 | 4.26287991058521 | 0.000145140206473 | Temporal variance in right hemisphere cortical Gordon parcel 227 (temporal _sup_R) |
| Baseline = 0, Development > 0 | | | | | | | | | |
| rsfmri_var_cdk_cdaclatelh | 0 | 0 | 0 | 0 | 3.06021755586571 | 0.007227351621221 | 3.48532697470065 | 0.002605591705143 | Temporal variance in APARC ROI left caudalanteriorcingulate |
| rsfmri_var_cdk_tvtlh | 0 | 0 | 0 | 0 | 5.56535911612216 | 2.87302496563885e-07 | 3.66348813477774 | 0.001432478809747 | Temporal variance in APARC ROI left transverseTemporal |
| rsfmri_var_cdk_tvtrh | 0 | 0 | 0 | 0 | 5.9845525873245 | 2.61804160602977e-08 | 3.42788721143631 | 0.00307737458011 | Temporal variance in APARC ROI right transverseTemporal |
| rsfmri_var_cortgordon_gp81lh | 0 | 0 | 0 | 0 | 4.95838570111184 | 6.21537926313991e-06 | 3.28834053190588 | 0.004804689669073 | Temporal variance in left hemisphere cortical Gordon parcel 81 (insula_L) |
| rsfmri_var_cortgordon_gp270rh | 0 | 0 | 0 | 0 | 3.53982246087373 | 0.001667340470722 | 3.00854336704921 | 0.010844700874475 | Temporal variance in right hemisphere cortical Gordon parcel 270 (supramarginal_R) |
| Baseline > 0, Development = 0 | | | | | | | | | |
| rsfmri_c_ngd_cgc_ngd_n | 4.0228910334032 | 0.000145592927963 | 4.82549047922714 | 3.76674012026353e-06 | 0 | 0 | 0 | 0 | Average correlation between cingulo opercular network and none network |
| rsfmri_c_ngd_cgc_ngd_rspltp | 7.50421718858897 | 4.15531595047249e-13 | 8.81506232069689 | 1.16877113749882e-17 | 0 | 0 | 0 | 0 | Average correlation between cingulo opercular network and retrosplenial temporal network |
| rsfmri_c_ngd_ca_ngd_dt | 3.82924600119915 | 0.000304764823738 | 3.77872742366172 | 0.000341846435866 | 0 | 0 | 0 | 0 | Average correlation between cingulo parietal network and default network |
| rsfmri_c_ngd_dt_ngd_ca | 3.82924600119915 | 0.000304764823738 | 3.77872742366172 | 0.000341846435866 | 0 | 0 | 0 | 0 | Average correlation between default network and cingulo parietal network |
| rsfmri_c_ngd_dt_ngd_dla | 6.59781766184634 | 2.0609587163481402e-10 | 7.71433024294348 | 7.70710389149546e-14 | 0 | 0 | 0 | 0 | Average correlation between default network and dorsal attention network |
| rsfmri_c_ngd_dt_ngd_fo | 5.24554100879092 | 5.2567911882808e-07 | 4.21388907416603 | 5.94280679954199e-05 | 0 | 0 | 0 | 0 | Average correlation between default network and fronto parietal network |
| rsfmri_c_ngd_dla_ngd_dt | 6.59781766184634 | 2.0609587163481402e-10 | 7.71433024294348 | 7.70710389149546e-14 | 0 | 0 | 0 | 0 | Average correlation between dorsal attention network and default network |
| rsfmri_c_ngd_dla_ngd_n | 6.33046144820029 | 1.1233904615551e-09 | 7.30901960292537 | 1.50656389601698e-12 | 0 | 0 | 0 | 0 | Average correlation between dorsal attention network and none network |
| rsfmri_c_ngd_dla_ngd_vta | 7.84742346471843 | 3.17026764596153e-14 | 7.87050487196811 | 2.39612605168255e-14 | 0 | 0 | 0 | 0 | Average correlation between dorsal attention network and ventral attention network |
| rsfmri_c_ngd_fo_ngd_dt | 5.24554100879092 | 5.2567911882808e-07 | 4.21388907416603 | 5.94280679954199e-05 | 0 | 0 | 0 | 0 | Average correlation between fronto parietal network and default network |
| rsfmri_c_ngd_n_ngd_cgc | 4.0228910334032 | 0.000145592927963 | 4.82549047922714 | 3.76674012026353e-06 | 0 | 0 | 0 | 0 | Average correlation between none network and cingulo opercular network |
| rsfmri_c_ngd_n_ngd_dla | 6.33046144820029 | 1.1233904615551e-09 | 7.30901960292537 | 1.50656389601698e-12 | 0 | 0 | 0 | 0 | Average correlation between none network and dorsal attention network |
| rsfmri_c_ngd_n_ngd_vs | 5.92257101327243 | 1.31084812931926e-08 | 3.08057810683403 | 0.003846443365608 | 0 | 0 | 0 | 0 | Average correlation between none network and visual network |
| rsfmri_c_ngd_rspltp_ngd_cgc | 7.50421718858897 | 4.15531595047249e-13 | 8.81506232069689 | 1.16877113749882e-17 | 0 | 0 | 0 | 0 | Average correlation between retrosplenial temporal network and cingulo opercular network |
| rsfmri_c_ngd_rspltp_ngd_smm | 4.04280326892946 | 0.000135007610716 | 7.01971664368531 | 1.13901717577485e-11 | 0 | 0 | 0 | 0 | Average correlation between retrosplenial temporal network and sensorimotor mouth network |
| rsfmri_c_ngd_rspltp_ngd_vta | 3.14428522334377 | 0.00338649770742 | 3.1625088960319 | 0.002973556350552 | 0 | 0 | 0 | 0 | Average correlation between retrosplenial temporal network and ventral attention network |
| rsfmri_c_ngd_smm_ngd_rspltp | 4.04280326892946 | 0.000135007610716 | 7.01971664368531 | 1.13901717577485e-11 | 0 | 0 | 0 | 0 | Average correlation between sensorimotor mouth network and retrosplenial temporal network |
| rsfmri_c_ngd_vta_ngd_dla | 7.84742346471843 | 3.17026764596153e-14 | 7.87050487196811 | 2.39612605168255e-14 | 0 | 0 | 0 | 0 | Average correlation between ventral attention network and dorsal attention network |
| rsfmri_c_ngd_vta_ngd_rspltp | 3.14428522334377 | 0.00338649770742 | 3.1625088960319 | 0.002973556350552 | 0 | 0 | 0 | 0 | Average correlation between ventral attention network and retrosplenial temporal network |
| rsfmri_c_ngd_vta_ngd_vs | 8.70895144122433 | 3.0910352948947703e-17 | 8.41546486791601 | 3.14599871848051e-16 | 0 | 0 | 0 | 0 | Average correlation between ventral attention network and visual network |
| rsfmri_c_ngd_vs_ngd_n | 5.92257101327243 | 1.31084812931926e-08 | 3.08057810683403 | 0.003846443365608 | 0 | 0 | 0 | 0 | Average correlation between visual network and none network |
| rsfmri_c_ngd_vs_ngd_vta | 8.70895144122433 | 3.0910352948947703e-17 | 8.41546486791601 | 3.14599871848051e-16 | 0 | 0 | 0 | 0 | Average correlation between visual network and ventral attention network |
| rsfmri_var_cdk_cdmdflh | 2.76788938200421 | 0.010303938893806 | 5.71249356490546 | 3.70188054562247e-08 | 0 | 0 | 0 | 0 | Temporal variance in APARC ROI left caudalmiddlefrontal |
| rsfmri_var_cdk_postcentrallh | 14.2806562502533 | 2.0038568705874997e-43 | 18.8282871899419 | 2.04804212072324e-72 | 0 | 0 | 0 | 0 | Temporal variance in APARC ROI left postcentral |
| rsfmri_var_cdk_psclatelh | 3.65889143565275 | 0.000580341788963 | 6.3744301486375 | 7.41414964097745e-10 | 0 | 0 | 0 | 0 | Temporal variance in APARC ROI left posteriorcingulate |
| rsfmri_var_cdk_precentrallh | 7.37336332864847 | 1.05817079532109e-12 | 11.0073561858646 | 1.21188632967548e-26 | 0 | 0 | 0 | 0 | Temporal variance in APARC ROI left precentral |
| rsfmri_var_cdk_cdmdfrh | 3.93484134524525 | 0.000207331380787 | 4.47019427816003 | 1.94093595040639e-05 | 0 | 0 | 0 | 0 | Temporal variance in APARC ROI right caudalmiddlefrontal |
| rsfmri_var_cdk_psclaterh | 3.98897650297465 | 0.000165979408704 | 6.52309577505387 | 2.91000645179193e-10 | 0 | 0 | 0 | 0 | Temporal variance in APARC ROI right posteriorcingulate |
| rsfmri_var_cdk_precentralrh | 9.49378236096976 | 3.23721340100657e-20 | 12.184564022706 | 3.09459871941817e-32 | 0 | 0 | 0 | 0 | Temporal variance in APARC ROI right precentral |
| rsfmri_var_cortgordon_gp12lh | 3.34683838864489 | 0.001737651158826 | 6.46358937718635 | 4.23790817496157e-10 | 0 | 0 | 0 | 0 | Temporal variance in left hemisphere cortical Gordon parcel 12 |
| rsfmri_var_cortgordon_gp21lh | 2.81853449476428 | 0.008968083819658 | 6.00135732361309 | 6.940680884860891e-09 | 0 | 0 | 0 | 0 | Temporal variance in left hemisphere cortical Gordon parcel 21 |
| rsfmri_var_cortgordon_gp32lh | 12.0372061731404 | 1.51566186763799e-31 | 15.3741748517163 | 8.85196121118496e-50 | 0 | 0 | 0 | 0 | Temporal variance in left hemisphere cortical Gordon parcel 32 (paracentral_lobule_L) |
| rsfmri_var_cortgordon_gp37lh | 6.63376236058864 | 1.63892049672814e-10 | 9.58511034547365 | 1.3059565777686e-20 | 0 | 0 | 0 | 0 | Temporal variance in left hemisphere cortical Gordon parcel 37 |
| rsfmri_var_cortgordon_gp46lh | 8.92976105422782 | 4.731147039509969e-18 | 8.57487027691081 | 8.69581538070181e-17 | 0 | 0 | 0 | 0 | Temporal variance in left hemisphere cortical Gordon parcel 46 (precentral_L) |
| rsfmri_var_cortgordon_gp65lh | 3.92664746572518 | 0.000213257611016 | 10.839865330179 | 6.714630563421589e-26 | 0 | 0 | 0 | 0 | Temporal variance in left hemisphere cortical Gordon parcel 65 (Rolandic Operculum L) |
| rsfmri_var_cortgordon_gp68lh | 5.76668241443657 | 3.13969961074119e-08 | 4.75437669932715 | 5.24066861241021e-06 | 0 | 0 | 0 | 0 | Temporal variance in left hemisphere cortical Gordon parcel 68 (Temporal_ sup_L) |
| rsfmri_var_cortgordon_gp77lh | 3.07920507519981 | 0.004149233333565 | 6.59950729024036 | 1.7813376331877301e-10 | 0 | 0 | 0 | 0 | Temporal variance in left hemisphere cortical Gordon parcel 77 (insula _L) |
| rsfmri_var_cortgordon_gp171rh | 6.23920915403407 | 1.9663610835418402e-09 | 6.23071483924901 | 1.7818239420332502e-09 | 0 | 0 | 0 | 0 | Temporal variance in right hemisphere cortical Gordon parcel 171 (insula_R) |
| rsfmri_var_cortgordon_gp173rh | 4.21714069234015 | 6.60957282011203e-05 | 5.7591331115353 | 2.84195291679773e-08 | 0 | 0 | 0 | 0 | Temporal variance in right hemisphere cortical Gordon parcel 173 () |
| rsfmri_var_cortgordon_gp180rh | 4.63019698454538 | 1.07238051107311e-05 | 8.1180925624764 | 3.4836099336087103e-15 | 0 | 0 | 0 | 0 | Temporal variance in right hemisphere cortical Gordon parcel 180 () |
| rsfmri_var_cortgordon_gp182rh | 3.48960168707759 | 0.001059716093591 | 3.66216617839599 | 0.000527711797766 | 0 | 0 | 0 | 0 | Temporal variance in right hemisphere cortical Gordon parcel 182 (supplementary motor area _R) |
| rsfmri_var_cortgordon_gp194rh | 9.06684441598659 | 1.45563781334035e-18 | 7.50204330686872 | 3.7848381151585e-13 | 0 | 0 | 0 | 0 | Temporal variance in right hemisphere cortical Gordon parcel 194 (supplementary motor area _R) |
| rsfmri_var_cortgordon_gp195rh | 5.70324308081464 | 4.46957167578623e-08 | 7.75707779624792 | 5.6400534470268205e-14 | 0 | 0 | 0 | 0 | Temporal variance in right hemisphere cortical Gordon parcel 195 () |
| rsfmri_var_cortgordon_gp202rh | 12.3545535672871 | 4.05597628210699e-33 | 13.3491634641962 | 3.18290221900643e-38 | 0 | 0 | 0 | 0 | Temporal variance in right hemisphere cortical Gordon parcel 202 (precentral R) |
| rsfmri_var_cortgordon_gp204rh | 6.99979658822542 | 1.3942113345373399e-11 | 11.0879782189045 | 5.21162293417331e-27 | 0 | 0 | 0 | 0 | Temporal variance in right hemisphere cortical Gordon parcel 204 (precentral R)) |
| rsfmri_var_cortgordon_gp206rh | 3.72085804111834 | 0.00045792657337 | 4.612660438 | 1.01603240335486e-05 | 0 | 0 | 0 | 0 | Temporal variance in right hemisphere cortical Gordon parcel 206 (precentral R) |
| rsfmri_var_cortgordon_gp207rh | 3.18373656109631 | 0.003015924793346 | 3.53003406559102 | 0.000843512051333 | 0 | 0 | 0 | 0 | Temporal variance in right hemisphere cortical Gordon parcel 207 (frontal sup R) |
| rsfmri_var_cortgordon_gp214rh | 17.2811553260285 | 5.49938816055458e-62 | 17.5775700605632 | 7.9598241095264e-64 | 0 | 0 | 0 | 0 | Temporal variance in right hemisphere cortical Gordon parcel 214 (postcentral R) |
| rsfmri_var_cortgordon_gp218rh | 4.27188539318379 | 5.25064537564265e-05 | 7.66262884577748 | 1.13694946825742e-13 | 0 | 0 | 0 | 0 | Temporal variance in right hemisphere cortical Gordon parcel 218  (precentral R) |
| rsfmri_var_cortgordon_gp224rh | 7.0375803734098 | 1.0750365986491399e-11 | 14.9416961565715 | 3.41382741747023e-47 | 0 | 0 | 0 | 0 | Temporal variance in right hemisphere cortical Gordon parcel 224 (rolandic R) |
| rsfmri_var_cortgordon_gp267rh | 2.6934466880014 | 0.012742718936212 | 9.70030339999801 | 4.59382229890805e-21 | 0 | 0 | 0 | 0 | Temporal variance in right hemisphere cortical Gordon parcel 267  (temporal mid R) |
| rsfmri_var_cortgordon_gp311rh | 3.64638742790752 | 0.000607542264995 | 9.27470655800638 | 2.15371734391849e-19 | 0 | 0 | 0 | 0 | Temporal variance in right hemisphere cortical Gordon parcel 311 (cuneus R) |
| rsfmri_var_scs_tplh | 12.5923098037913 | 2.53406134699505e-34 | 12.5390864558055 | 5.25016835892404e-34 | 0 | 0 | 0 | 0 | Temporal variance in ASEG ROI left thalamus proper |
| rsfmri_var_scs_caudatelh | 5.3251112089729 | 3.5103781124097e-07 | 6.90030693532444 | 2.5765603931705798e-11 | 0 | 0 | 0 | 0 | Temporal variance in ASEG ROI left caudate |
| rsfmri_var_scs_csf | 8.41678913876424 | 3.50363469553613e-16 | 5.6296689812831 | 5.8818785468398e-08 | 0 | 0 | 0 | 0 | Temporal variance in ASEG ROI csf |
| rsfmri_var_scs_tprh | 12.2011797433471 | 2.32236911247522e-32 | 14.7511953982951 | 4.38429715313013e-46 | 0 | 0 | 0 | 0 | Temporal variance in ASEG ROI right thalamus proper |
| rsfmri_var_scs_caudaterh | 5.15163198525855 | 8.47688648255117e-07 | 7.24950696800951 | 2.28566162729019e-12 | 0 | 0 | 0 | 0 | Temporal variance in ASEG ROI right caudate |
| rsfmri_cor_ngd_copa_scs_ptlh | 9.04532004318698 | 1.72284372827118e-18 | 7.49821365296111 | 3.87502937647434e-13 | 0 | 0 | 0 | 0 | Average correlation between cingulo parietal network and ASEG ROI left putamen |
| rsfmri_cor_ngd_copa_scs_agrh | 8.54321100194489 | 1.24343927023498e-16 | 6.60849230582536 | 1.68534319516055e-10 | 0 | 0 | 0 | 0 | Average correlation between cingulo parietal network and ASEG ROI right amygdala |
| rsfmri_cor_ngd_df_scs_bs | 3.58368579970299 | 0.000764835485397 | 5.32899201195339 | 3.01310339086267e-07 | 0 | 0 | 0 | 0 | Average correlation between default network and ASEG ROI brain stem |
| rsfmri_cor_ngd_df_scs_crcxrh | 5.92364654687967 | 1.31084812931926e-08 | 7.46880289658116 | 4.77420156799029e-13 | 0 | 0 | 0 | 0 | Average correlation between default network and ASEG ROI right cerebellum cortex |
| rsfmri_cor_ngd_df_scs_vtdcrh | 6.55440692763278 | 2.71928766342456e-10 | 5.98207195155431 | 7.74465562000392e-09 | 0 | 0 | 0 | 0 | Average correlation between default network and ASEG ROI right ventraldc |
| rsfmri_cor_ngd_dsa_scs_ptlh | 5.07418116588715 | 1.25256381256429e-06 | 6.42529535528531 | 5.36603525163391e-10 | 0 | 0 | 0 | 0 | Average correlation between dorsal attention network and ASEG ROI left putamen |
| rsfmri_cor_ngd_dsa_scs_hplh | 5.98952050274661 | 8.90809855555012e-09 | 6.069296944 | 4.693628366928161e-09 | 0 | 0 | 0 | 0 | Average correlation between dorsal attention network and ASEG ROI left hippocampus |
| rsfmri_cor_ngd_dsa_scs_plrh | 5.75490203574678 | 3.34991506918106e-08 | 4.609663532 | 1.0277228119407e-05 | 0 | 0 | 0 | 0 | Average correlation between dorsal attention network and ASEG ROI right pallidum |
| rsfmri_cor_ngd_dsa_scs_agrh | 3.51469955519664 | 0.000974220627674 | 3.49586595534494 | 0.000947502857364 | 0 | 0 | 0 | 0 | Average correlation between dorsal attention network and ASEG ROI right amygdala |
| rsfmri_cor_ngd_fopa_scs_crcxlh | 4.55426965185825 | 1.513407066927e-05 | 4.24509534968506 | 5.2207162896216e-05 | 0 | 0 | 0 | 0 | Average correlation between fronto parietal network and ASEG ROI left cerebellum cortex |
| rsfmri_cor_ngd_fopa_scs_aalh | 6.79873348080866 | 5.5028622792309394e-11 | 6.15533267853412 | 2.8096763853684503e-09 | 0 | 0 | 0 | 0 | Average correlation between fronto parietal network and ASEG ROI left accumbens area |
| rsfmri_cor_ngd_smm_scs_crcxlh | 8.33378899858015 | 6.86257575757306e-16 | 7.8291104222226 | 3.2864388484175103e-14 | 0 | 0 | 0 | 0 | Average correlation between sensorimotor mouth network and ASEG ROI left cerebellum cortex |
| rsfmri_cor_ngd_smm_scs_ptlh | 6.67551314327644 | 1.25833131999932e-10 | 3.67403604584712 | 0.000505142513915 | 0 | 0 | 0 | 0 | Average correlation between sensorimotor mouth network and ASEG ROI left putamen |
| rsfmri_cor_ngd_smm_scs_ptrh | 5.99785570739638 | 8.506029620040341e-09 | 7.49468633588113 | 3.95849955246684e-13 | 0 | 0 | 0 | 0 | Average correlation between sensorimotor mouth network and ASEG ROI right putamen |
| rsfmri_cor_ngd_smm_scs_plrh | 6.44711580414115 | 5.40492600313056e-10 | 6.49455653525136 | 3.47841889733892e-10 | 0 | 0 | 0 | 0 | Average correlation between sensorimotor mouth network and ASEG ROI right pallidum |
| rsfmri_cor_ngd_sa_scs_cdelh | 3.81081258207194 | 0.000327080965334 | 6.01020394763169 | 6.6255856081342504e-09 | 0 | 0 | 0 | 0 | Average correlation between salience network and ASEG ROI left caudate |
| rsfmri_cor_ngd_sa_scs_hplh | 5.33295573880744 | 3.37634968342136e-07 | 6.06911572575791 | 4.693628366928161e-09 | 0 | 0 | 0 | 0 | Average correlation between salience network and ASEG ROI left hippocampus |
| rsfmri_cor_ngd_sa_scs_vtdclh | 6.30066075852969 | 1.3456326555298201e-09 | 4.00186809699372 | 0.00014206872288 | 0 | 0 | 0 | 0 | Average correlation between salience network and ASEG ROI left ventraldc |
| rsfmri_cor_ngd_vta_scs_crcxlh | 3.44431881068035 | 0.001245680887556 | 4.68330490751221 | 7.34242243663598e-06 | 0 | 0 | 0 | 0 | Average correlation between ventral attention network and ASEG ROI left cerebellum cortex |
| rsfmri_cor_ngd_vta_scs_ptlh | 4.93103938685896 | 2.56123057922953e-06 | 5.02946197256401 | 1.39528433426618e-06 | 0 | 0 | 0 | 0 | Average correlation between ventral attention network and ASEG ROI left putamen |
| Baseline < 0, Development = 0 | | | | | | | | | |
| rsfmri_c_ngd_ad_ngd_n | -3.513491252 | 0.000974220627674 | -4.46809623 | 1.94903998423803e-05 | 0 | 0 | 0 | 0 | Average correlation between auditory network and none network |
| rsfmri_c_ngd_cgc_ngd_cgc | -9.56712052 | 1.66330367878324e-20 | -9.207622346 | 3.8442736430284503e-19 | 0 | 0 | 0 | 0 | Average correlation between cingulo opercular network and cingulo opercular network |
| rsfmri_c_ngd_ca_ngd_vta | -4.780194379 | 5.31493955470563e-06 | -6.666008596 | 1.18357367949167e-10 | 0 | 0 | 0 | 0 | Average correlation between cingulo parietal network and ventral attention network |
| rsfmri_c_ngd_ca_ngd_vs | -4.963515664 | 2.17895800909771e-06 | -4.118762879 | 8.82417628055702e-05 | 0 | 0 | 0 | 0 | Average correlation between cingulo parietal network and visual network |
| rsfmri_c_ngd_dt_ngd_n | -5.542865531 | 1.08328988474855e-07 | -6.61659628 | 1.60377695001897e-10 | 0 | 0 | 0 | 0 | Average correlation between default network and none network |
| rsfmri_c_ngd_dt_ngd_rspltp | -2.943603246 | 0.006214259040589 | -4.348659397 | 3.32098667701225e-05 | 0 | 0 | 0 | 0 | Average correlation between default network and retrosplenial temporal network |
| rsfmri_c_ngd_dla_ngd_dla | -5.791816024 | 2.74292677075795e-08 | -6.331132531 | 9.7008965824899e-10 | 0 | 0 | 0 | 0 | Average correlation between dorsal attention network and dorsal attention network |
| rsfmri_c_ngd_n_ngd_ad | -3.513491252 | 0.000974220627674 | -4.46809623 | 1.94903998423803e-05 | 0 | 0 | 0 | 0 | Average correlation between none network and auditory network |
| rsfmri_c_ngd_n_ngd_dt | -5.542865531 | 1.08328988474855e-07 | -6.61659628 | 1.60377695001897e-10 | 0 | 0 | 0 | 0 | Average correlation between none network and default network |
| rsfmri_c_ngd_n_ngd_smh | -7.299801293 | 1.76281371701774e-12 | -9.249370168 | 2.65679213433015e-19 | 0 | 0 | 0 | 0 | Average correlation between none network and sensorimotor hand network |
| rsfmri_c_ngd_n_ngd_vta | -3.901245234 | 0.000235435651993 | -4.526239163 | 1.50731748682194e-05 | 0 | 0 | 0 | 0 | Average correlation between none network and ventral attention network |
| rsfmri_c_ngd_rspltp_ngd_dt | -2.943603246 | 0.006214259040589 | -4.348659397 | 3.32098667701225e-05 | 0 | 0 | 0 | 0 | Average correlation between retrosplenial temporal network and default network |
| rsfmri_c_ngd_rspltp_ngd_rspltp | -5.500685349 | 1.36059059178488e-07 | -2.897303764 | 0.006604876123871 | 0 | 0 | 0 | 0 | Average correlation between retrosplenial temporal network and retrosplenial temporal network |
| rsfmri_c_ngd_smh_ngd_n | -7.299801293 | 1.76281371701774e-12 | -9.249370168 | 2.65679213433015e-19 | 0 | 0 | 0 | 0 | Average correlation between sensorimotor hand network and none network |
| rsfmri_c_ngd_sa_ngd_vs | -4.021524032 | 0.000145593305977 | -3.648383237 | 0.000552640198485 | 0 | 0 | 0 | 0 | Average correlation between salience network and visual network |
| rsfmri_c_ngd_vta_ngd_ca | -4.780194379 | 5.31493955470563e-06 | -6.666008596 | 1.18357367949167e-10 | 0 | 0 | 0 | 0 | Average correlation between ventral attention network and cingulo parietal network |
| rsfmri_c_ngd_vta_ngd_n | -3.901245234 | 0.000235435651993 | -4.526239163 | 1.50731748682194e-05 | 0 | 0 | 0 | 0 | Average correlation between ventral attention network and none network |
| rsfmri_c_ngd_vta_ngd_vta | -5.231307558 | 5.65258742238588e-07 | -2.957011565 | 0.005534307668613 | 0 | 0 | 0 | 0 | Average correlation between ventral attention network and ventral attention network |
| rsfmri_c_ngd_vs_ngd_ca | -4.963515664 | 2.17895800909771e-06 | -4.118762879 | 8.82417628055702e-05 | 0 | 0 | 0 | 0 | Average correlation between visual network and cingulo parietal network |
| rsfmri_c_ngd_vs_ngd_sa | -4.021524032 | 0.000145593305977 | -3.648383237 | 0.000552640198485 | 0 | 0 | 0 | 0 | Average correlation between visual network and salience network |
| rsfmri_c_ngd_vs_ngd_vs | -6.022377049 | 7.360448503239151e-09 | -6.025091998 | 6.07428057212335e-09 | 0 | 0 | 0 | 0 | Average correlation between visual network and visual network |
| rsfmri_var_cdk_loboflh | -3.885954666 | 0.000247287977588 | -3.113459532 | 0.003474297351945 | 0 | 0 | 0 | 0 | Temporal variance in APARC ROI left lateralorbitofrontal |
| rsfmri_var_cdk_moboflh | -6.085163584 | 5.03494135728486e-09 | -4.913844194 | 2.46385454744171e-06 | 0 | 0 | 0 | 0 | Temporal variance in APARC ROI left medialorbitofrontal |
| rsfmri_var_cdk_mdtlh | -3.83190872 | 0.000303708987886 | -3.788883569 | 0.000329896347503 | 0 | 0 | 0 | 0 | Temporal variance in APARC ROI left middleTemporal |
| rsfmri_var_cdk_parsobalislh | -2.84656998 | 0.008271409204472 | -2.806376716 | 0.008624199159893 | 0 | 0 | 0 | 0 | Temporal variance in APARC ROI left parsorbitalis |
| rsfmri_var_cdk_rlaclatelh | -4.076711995 | 0.000117906658314 | -3.849296728 | 0.000260601306348 | 0 | 0 | 0 | 0 | Temporal variance in APARC ROI left rostralanteriorcingulate |
| rsfmri_var_cdk_tpolelh | -3.067391437 | 0.00430610911974 | -3.932221642 | 0.00018794554204 | 0 | 0 | 0 | 0 | Temporal variance in APARC ROI left Temporalpole |
| rsfmri_var_cdk_mobofrh | -5.818551697 | 2.37272123220314e-08 | -4.817364344 | 3.89855842057462e-06 | 0 | 0 | 0 | 0 | Temporal variance in APARC ROI right medialorbitofrontal |
| rsfmri_var_cdk_tpolerh | -3.230325671 | 0.00258547281611 | -4.717169513 | 6.24838322716938e-06 | 0 | 0 | 0 | 0 | Temporal variance in APARC ROI right Temporalpole |
| rsfmri_var_cortgordon_gp11lh | -2.803815757 | 0.009325768852142 | -2.75905777 | 0.009893751683401 | 0 | 0 | 0 | 0 | Temporal variance in left hemisphere cortical Gordon parcel 11 |
| rsfmri_var_cortgordon_gp117lh | -5.126594169 | 9.63647976198887e-07 | -3.704495592 | 0.000450662729707 | 0 | 0 | 0 | 0 | Temporal variance in left hemisphere cortical Gordon parcel 117 (middle frontal orbital L) |
| rsfmri_var_cortgordon_gp123lh | -4.405117269 | 2.95004914468402e-05 | -3.968583512 | 0.000162868124027 | 0 | 0 | 0 | 0 | Temporal variance in left hemisphere cortical Gordon parcel 123 (frontal sup orbital L) |
| rsfmri_var_cortgordon_gp124lh | -3.768386975 | 0.000384471735262 | -3.278013038 | 0.00203599006415 | 0 | 0 | 0 | 0 | Temporal variance in left hemisphere cortical Gordon parcel 124 (recutus L) |
| rsfmri_var_cortgordon_gp125lh | -2.997915339 | 0.005281181372809 | -4.118902289 | 8.82417628055702e-05 | 0 | 0 | 0 | 0 | Temporal variance in left hemisphere cortical Gordon parcel 125 () |
| rsfmri_var_cortgordon_gp184rh | -3.933099791 | 0.000208236886431 | -2.63147052 | 0.014017489919381 | 0 | 0 | 0 | 0 | Temporal variance in right hemisphere cortical Gordon parcel 184 () |
| rsfmri_var_cortgordon_gp242rh | -2.996167659 | 0.005287504925266 | -3.208237294 | 0.002569039747759 | 0 | 0 | 0 | 0 | Temporal variance in right hemisphere cortical Gordon parcel 242 (frontal inf orb R) |
| rsfmri_var_cortgordon_gp279rh | -3.43642734 | 0.00127068833528 | -2.672504014 | 0.012538941605751 | 0 | 0 | 0 | 0 | Temporal variance in right hemisphere cortical Gordon parcel 279 (frontal mid orb R) |
| rsfmri_var_cortgordon_gp287rh | -4.517675567 | 1.78580612584367e-05 | -4.808360953 | 4.05244616585763e-06 | 0 | 0 | 0 | 0 | Temporal variance in right hemisphere cortical Gordon parcel 287 (rectus _R) |
| rsfmri_var_cortgordon_gp288rh | -2.80910336 | 0.009214182130273 | -5.050372914 | 1.25626550302206e-06 | 0 | 0 | 0 | 0 | Temporal variance in right hemisphere cortical Gordon parcel 288 (rectus _R) |
| rsfmri_var_cortgordon_gp290rh | -2.639002383 | 0.014758242035418 | -3.570353636 | 0.000729463402244 | 0 | 0 | 0 | 0 | Temporal variance in right hemisphere cortical Gordon parcel 290 (temporal_mid_R) |
| rsfmri_var_cortgordon_gp292rh | -3.050987271 | 0.004495356944589 | -4.056437894 | 0.000114001953587 | 0 | 0 | 0 | 0 | Temporal variance in right hemisphere cortical Gordon parcel 292 (temporal pole mid R) |
| rsfmri_var_cortgordon_gp297rh | -2.635017044 | 0.014901220899826 | -2.751591225 | 0.010101249137489 | 0 | 0 | 0 | 0 | Temporal variance in right hemisphere cortical Gordon parcel 297 (fusiform_R) |
| rsfmri_var_cortgordon_gp301rh | -2.621564542 | 0.015436656790206 | -2.665457996 | 0.012779046717233 | 0 | 0 | 0 | 0 | Temporal variance in right hemisphere cortical Gordon parcel 301 (temporal inf R) |
| rsfmri_var_cortgordon_gp302rh | -3.128613416 | 0.003563197813246 | -3.646130532 | 0.000556136286276 | 0 | 0 | 0 | 0 | Temporal variance in right hemisphere cortical Gordon parcel 302 (temporal inf R) |
| rsfmri_var_scs_crbcortexlh | -2.729155535 | 0.011493833356904 | -3.03233901 | 0.004476182921739 | 0 | 0 | 0 | 0 | Temporal variance in ASEG ROI left cerebellum cortex |
| rsfmri_var_scs_brainstem | -2.787668745 | 0.009738914752249 | -3.050607204 | 0.004222749011574 | 0 | 0 | 0 | 0 | Temporal variance in ASEG ROI brain stem |
| rsfmri_var_scs_hpuslh | -3.398848123 | 0.001450586211285 | -3.063895663 | 0.004049070837812 | 0 | 0 | 0 | 0 | Temporal variance in ASEG ROI left hippocampus |
| rsfmri_var_scs_cbwmrh | -5.074622768 | 1.25256381256429e-06 | -3.107614452 | 0.003535724260745 | 0 | 0 | 0 | 0 | Temporal variance in ASEG ROI right cerebral white matter |
| rsfmri_var_scs_cbcortexrh | -5.222478264 | 5.88297254943902e-07 | -4.272596791 | 4.63345865876736e-05 | 0 | 0 | 0 | 0 | Temporal variance in ASEG ROI right cerebral cortex |
| rsfmri_var_scs_crbwmrh | -3.204346882 | 0.002815753688261 | -2.628894716 | 0.014096615666784 | 0 | 0 | 0 | 0 | Temporal variance in ASEG ROI right cerebellum white matter |
| rsfmri_cor_ngd_au_scs_aglh | -3.165950742 | 0.003167562446496 | -3.201175805 | 0.00262668576065 | 0 | 0 | 0 | 0 | Average correlation between auditory network and ASEG ROI left amygdala |
| rsfmri_cor_ngd_cerc_scs_thplh | -3.541666576 | 0.000887639370411 | -6.322467376 | 1.02111609438011e-09 | 0 | 0 | 0 | 0 | Average correlation between cingulo opercular network and ASEG ROI left thalamus proper |
| rsfmri_cor_ngd_df_scs_hprh | -2.800062801 | 0.009414234729138 | -3.600799132 | 0.000657311692784 | 0 | 0 | 0 | 0 | Average correlation between default network and ASEG ROI right hippocampus |
| rsfmri_cor_ngd_dsa_scs_crcxlh | -5.846948363 | 2.01441675317342e-08 | -8.462274132 | 2.15712127407027e-16 | 0 | 0 | 0 | 0 | Average correlation between dorsal attention network and ASEG ROI left cerebellum cortex |
| rsfmri_cor_ngd_dsa_scs_bs | -8.018170411 | 8.6028192286329e-15 | -10.18983999 | 4.60019056206614e-23 | 0 | 0 | 0 | 0 | Average correlation between dorsal attention network and ASEG ROI brain stem |
| rsfmri_cor_ngd_dsa_scs_aalh | -4.281736589 | 5.04060286784595e-05 | -3.244821557 | 0.002267127267194 | 0 | 0 | 0 | 0 | Average correlation between dorsal attention network and ASEG ROI left accumbens area |
| rsfmri_cor_ngd_dsa_scs_vtdclh | -3.873826533 | 0.000258300813654 | -3.820358894 | 0.000291470255333 | 0 | 0 | 0 | 0 | Average correlation between dorsal attention network and ASEG ROI left ventraldc |
| rsfmri_cor_ngd_dsa_scs_aarh | -4.923741646 | 2.64853898775797e-06 | -3.585276462 | 0.000692481107816 | 0 | 0 | 0 | 0 | Average correlation between dorsal attention network and ASEG ROI right accumbens area |
| rsfmri_cor_ngd_fopa_scs_cdelh | -6.091293509 | 4.87003486397128e-09 | -4.817615041 | 3.89855842057462e-06 | 0 | 0 | 0 | 0 | Average correlation between fronto parietal network and ASEG ROI left caudate |
| rsfmri_cor_ngd_fopa_scs_ptlh | -5.032971968 | 1.5416940169213e-06 | -6.124823282 | 3.3687560163561303e-09 | 0 | 0 | 0 | 0 | Average correlation between fronto parietal network and ASEG ROI left putamen |
| rsfmri_cor_ngd_fopa_scs_hprh | -4.744526457 | 6.26899573246612e-06 | -5.062322671 | 1.18450030505587e-06 | 0 | 0 | 0 | 0 | Average correlation between fronto parietal network and ASEG ROI right hippocampus |
| rsfmri_cor_ngd_none_scs_thplh | -5.25626512 | 4.99922233516334e-07 | -7.342196785 | 1.20614790735843e-12 | 0 | 0 | 0 | 0 | Average correlation between none network and ASEG ROI left thalamus proper |
| rsfmri_cor_ngd_none_scs_crcxrh | -3.081599924 | 0.00412572677541 | -5.160009667 | 7.30859523960535e-07 | 0 | 0 | 0 | 0 | Average correlation between none network and ASEG ROI right cerebellum cortex |
| rsfmri_cor_ngd_rst_scs_plrh | -3.766793589 | 0.00038588089337 | -5.897084086 | 1.27664639934148e-08 | 0 | 0 | 0 | 0 | Average correlation between retrosplenial temporal network and ASEG ROI right pallidum |
| rsfmri_cor_ngd_rst_scs_aarh | -4.11893858 | 0.000100745972575 | -4.490617929 | 1.77517731542874e-05 | 0 | 0 | 0 | 0 | Average correlation between retrosplenial temporal network and ASEG ROI right accumbens area |
| rsfmri_cor_ngd_sa_scs_ptlh | -7.365052267 | 1.11856679090735e-12 | -7.305078478 | 1.5429647745806199e-12 | 0 | 0 | 0 | 0 | Average correlation between salience network and ASEG ROI left putamen |
| rsfmri_cor_ngd_sa_scs_pllh | -3.470623471 | 0.001134228750005 | -4.145793302 | 7.9142680074829e-05 | 0 | 0 | 0 | 0 | Average correlation between salience network and ASEG ROI left pallidum |
| rsfmri_cor_ngd_sa_scs_thprh | -4.714687127 | 7.20359744354016e-06 | -3.521642195 | 0.00086848257681 | 0 | 0 | 0 | 0 | Average correlation between salience network and ASEG ROI right thalamus proper |
| rsfmri_cor_ngd_sa_scs_cderh | -4.246142882 | 5.85043842235652e-05 | -4.212786277 | 5.95571324155273e-05 | 0 | 0 | 0 | 0 | Average correlation between salience network and ASEG ROI right caudate |
| rsfmri_cor_ngd_vta_scs_thplh | -5.77757694 | 2.95765754871008e-08 | -3.927539427 | 0.000191128340803 | 0 | 0 | 0 | 0 | Average correlation between ventral attention network and ASEG ROI left thalamus proper |
| rsfmri_cor_ngd_vta_scs_hplh | -5.304622699 | 3.89410764887232e-07 | -5.363707607 | 2.5180794150558e-07 | 0 | 0 | 0 | 0 | Average correlation between ventral attention network and ASEG ROI left hippocampus |
| rsfmri_cor_ngd_vta_scs_vtdcrh | -4.473046765 | 2.19166650969042e-05 | -4.624224831 | 9.66975044887742e-06 | 0 | 0 | 0 | 0 | Average correlation between ventral attention network and ASEG ROI right ventraldc |
| rsfmri_cor_ngd_vs_scs_cdelh | -7.331234098 | 1.41710650892623e-12 | -5.100812602 | 9.8474589524216e-07 | 0 | 0 | 0 | 0 | Average correlation between visual network and ASEG ROI left caudate |
| rsfmri_cor_ngd_vs_scs_pllh | -5.778753562 | 2.94932191659055e-08 | -6.133496447 | 3.2047854098590303e-09 | 0 | 0 | 0 | 0 | Average correlation between visual network and ASEG ROI left pallidum |
| rsfmri_cor_ngd_vs_scs_ptrh | -3.829023145 | 0.000304764823738 | -5.472585465 | 1.39893206587115e-07 | 0 | 0 | 0 | 0 | Average correlation between visual network and ASEG ROI right putamen |
| Baseline > 0, Development < 0 | | | | | | | | | |
| rsfmri_c_ngd_ad_ngd_ad | 8.81067547563401 | 1.3148518361661201e-17 | 12.2412892739484 | 1.6232309219346002e-32 | -6.194655123 | 7.76531246488126e-09 | -6.390598308 | 2.6524846769681503e-09 | Average correlation between auditory network and auditory network |
| rsfmri_c_ngd_ad_ngd_rspltp | 7.1410522356492 | 5.32799253154352e-12 | 9.77499128356893 | 2.3164120010175e-21 | -3.376276198 | 0.00277062626951 | -4.423272246 | 7.39474325087174e-05 | Average correlation between auditory network and retrosplenial temporal network |
| rsfmri_c_ngd_ad_ngd_smh | 29.4598454180347 | 3.2479790582759104e-161 | 31.2971603269768 | 3.23946532531658e-177 | -14.30224811 | 5.7972397622492e-42 | -12.85872715 | 1.9686467224641798e-34 | Average correlation between auditory network and sensorimotor hand network |
| rsfmri_c_ngd_ad_ngd_smm | 17.2649503523891 | 6.82061820122399e-62 | 19.1456438974515 | 1.17710133069424e-74 | -8.216787392 | 7.55378440091806e-15 | -8.277400025 | 4.8573117434650405e-15 | Average correlation between auditory network and sensorimotor mouth network |
| rsfmri_c_ngd_ad_ngd_vs | 18.1548133906315 | 7.0988501260972404e-68 | 19.0830909055897 | 3.1808902771207303e-74 | -8.261571556 | 5.540806446943311e-15 | -7.239708676 | 1.00007539533531e-11 | Average correlation between auditory network and visual network |
| rsfmri_c_ngd_ca_ngd_sa | 9.06587556851785 | 1.45563781334035e-18 | 6.28833008936878 | 1.24391785744257e-09 | -4.357496111 | 8.44416720516749e-05 | -3.130657844 | 0.007699033363614 | Average correlation between cingulo parietal network and salience network |
| rsfmri_c_ngd_fo_ngd_fo | 5.07372007057702 | 1.25256381256429e-06 | 5.58234071577097 | 7.64956663257355e-08 | -3.378640775 | 0.00277062626951 | -3.667271501 | 0.001420990281383 | Average correlation between fronto parietal network and fronto parietal network |
| rsfmri_c_ngd_fo_ngd_sa | 6.64750795261643 | 1.50268396513163e-10 | 7.16436832396338 | 4.1330661328599e-12 | -4.512437642 | 4.53170872645243e-05 | -4.303934835 | 0.000123874687799 | Average correlation between fronto parietal network and salience network |
| rsfmri_c_ngd_rspltp_ngd_ad | 7.1410522356492 | 5.32799253154352e-12 | 9.77499128356893 | 2.3164120010175e-21 | -3.376276198 | 0.00277062626951 | -4.423272246 | 7.39474325087174e-05 | Average correlation between retrosplenial temporal network and auditory network |
| rsfmri_c_ngd_smh_ngd_ad | 29.4598454180347 | 3.2479790582759104e-161 | 31.2971603269768 | 3.23946532531658e-177 | -14.30224811 | 5.7972397622492e-42 | -12.85872715 | 1.9686467224641798e-34 | Average correlation between sensorimotor hand network and auditory network |
| rsfmri_c_ngd_smh_ngd_smh | 32.9966987340964 | 5.3221948201104e-195 | 31.8021866178374 | 7.326763253196591e-182 | -11.6988352 | 7.39421161806064e-29 | -11.19654939 | 1.39378806259772e-26 | Average correlation between sensorimotor hand network and sensorimotor hand network |
| rsfmri_c_ngd_smh_ngd_smm | 37.0972585637301 | 6.703975430707451e-236 | 36.2770100997169 | 2.00219004984975e-225 | -13.71927456 | 3.81686869493338e-39 | -11.55396975 | 4.04324750268609e-28 | Average correlation between sensorimotor hand network and sensorimotor mouth network |
| rsfmri_c_ngd_smh_ngd_vta | 5.39052445358394 | 2.48272852336853e-07 | 5.09723818153208 | 9.9699166455301e-07 | -3.901551636 | 0.000488155464822 | -4.161487068 | 0.000219468909342 | Average correlation between sensorimotor hand network and ventral attention network |
| rsfmri_c_ngd_smh_ngd_vs | 20.1464943139387 | 3.627419731637e-82 | 21.0991500651214 | 4.70843346566054e-89 | -7.555488722 | 9.63789916800356e-13 | -7.660856653 | 4.90362155621289e-13 | Average correlation between sensorimotor hand network and visual network |
| rsfmri_c_ngd_smm_ngd_ad | 17.2649503523891 | 6.82061820122399e-62 | 19.1456438974515 | 1.17710133069424e-74 | -8.216787392 | 7.55378440091806e-15 | -8.277400025 | 4.8573117434650405e-15 | Average correlation between sensorimotor mouth network and auditory network |
| rsfmri_c_ngd_smm_ngd_smh | 37.0972585637301 | 6.703975430707451e-236 | 36.2770100997169 | 2.00219004984975e-225 | -13.71927456 | 3.81686869493338e-39 | -11.55396975 | 4.04324750268609e-28 | Average correlation between sensorimotor mouth network and sensorimotor hand network |
| rsfmri_c_ngd_smm_ngd_smm | 7.55257156055928 | 2.92721189143326e-13 | 6.95862314940543 | 1.7285825922843798e-11 | -4.102785466 | 0.000228369663923 | -4.43181114 | 7.29476693544941e-05 | Average correlation between sensorimotor mouth network and sensorimotor mouth network |
| rsfmri_c_ngd_smm_ngd_vs | 5.80223436293765 | 2.5906062895755e-08 | 6.82572165603517 | 4.1662677990363594e-11 | -3.709371873 | 0.000950600456229 | -3.309770794 | 0.004477909203559 | Average correlation between sensorimotor mouth network and visual network |
| rsfmri_c_ngd_sa_ngd_ca | 9.06587556851785 | 1.45563781334035e-18 | 6.28833008936878 | 1.24391785744257e-09 | -4.357496111 | 8.44416720516749e-05 | -3.130657844 | 0.007699033363614 | Average correlation between salience network and cingulo parietal network |
| rsfmri_c_ngd_sa_ngd_fo | 6.64750795261643 | 1.50268396513163e-10 | 7.16436832396338 | 4.1330661328599e-12 | -4.512437642 | 4.53170872645243e-05 | -4.303934835 | 0.000123874687799 | Average correlation between salience network and fronto parietal network |
| rsfmri_c_ngd_vta_ngd_smh | 5.39052445358394 | 2.48272852336853e-07 | 5.09723818153208 | 9.9699166455301e-07 | -3.901551636 | 0.000488155464822 | -4.161487068 | 0.000219468909342 | Average correlation between ventral attention network and sensorimotor hand network |
| rsfmri_c_ngd_vs_ngd_ad | 18.1548133906315 | 7.0988501260972404e-68 | 19.0830909055897 | 3.1808902771207303e-74 | -8.261571556 | 5.540806446943311e-15 | -7.239708676 | 1.00007539533531e-11 | Average correlation between visual network and auditory network |
| rsfmri_c_ngd_vs_ngd_smh | 20.1464943139387 | 3.627419731637e-82 | 21.0991500651214 | 4.70843346566054e-89 | -7.555488722 | 9.63789916800356e-13 | -7.660856653 | 4.90362155621289e-13 | Average correlation between visual network and sensorimotor hand network |
| rsfmri_c_ngd_vs_ngd_smm | 5.80223436293765 | 2.5906062895755e-08 | 6.82572165603517 | 4.1662677990363594e-11 | -3.709371873 | 0.000950600456229 | -3.309770794 | 0.004477909203559 | Average correlation between visual network and sensorimotor mouth network |
| rsfmri_cor_ngd_au_scs_cdelh | 7.67635849800128 | 1.16840592377761e-13 | 8.79276121925799 | 1.39998043396627e-17 | -2.915338137 | 0.01077049762628 | -4.124744891 | 0.000254753321946 | Average correlation between auditory network and ASEG ROI left caudate |
| rsfmri_cor_ngd_au_scs_ptlh | 13.6603396000751 | 5.785175535035329e-40 | 16.0726213481778 | 4.4118462355141404e-54 | -5.235892487 | 1.5636953616242e-06 | -6.596267268 | 7.27975540050482e-10 | Average correlation between auditory network and ASEG ROI left putamen |
| rsfmri_cor_ngd_au_scs_bs | 13.5480946936761 | 2.37779553925769e-39 | 13.5912332253497 | 1.56174198771178e-39 | -8.362976466 | 2.80335399586459e-15 | -8.526838988 | 6.54458600853892e-16 | Average correlation between auditory network and ASEG ROI brain stem |
| rsfmri_cor_ngd_au_scs_crcxrh | 9.88692607900618 | 8.53234618276167e-22 | 10.9979335826589 | 1.31639230888235e-26 | -4.050277691 | 0.000280392200283 | -5.883411729 | 5.36941647459552e-08 | Average correlation between auditory network and ASEG ROI right cerebellum cortex |
| rsfmri_cor_ngd_au_scs_hprh | 13.1719700159703 | 2.44969216380743e-37 | 12.4544737838529 | 1.407441743309e-33 | -3.097442567 | 0.00655614237925 | -3.462889737 | 0.002737408315765 | Average correlation between auditory network and ASEG ROI right hippocampus |
| rsfmri_cor_ngd_au_scs_agrh | 11.58548421 | 2.38725849724736e-29 | 9.39013876151084 | 7.63887217919105e-20 | -6.221332771 | 6.6810384647081905e-09 | -4.457607689 | 6.53628211120869e-05 | Average correlation between auditory network and ASEG ROI right amygdala |
| rsfmri_cor_ngd_au_scs_vtdcrh | 15.8829859817203 | 5.522541543198301e-53 | 16.8738895222269 | 3.52528898718696e-59 | -5.012270818 | 4.76282276107492e-06 | -5.600514559 | 2.64313309082119e-07 | Average correlation between auditory network and ASEG ROI right ventraldc |
| rsfmri_cor_ngd_cerc_scs_pllh | 8.47079788205823 | 2.25056007779274e-16 | 9.63964272042871 | 8.005526609679491e-21 | -3.558689622 | 0.001590874451335 | -3.035471935 | 0.010119802160454 | Average correlation between cingulo opercular network and ASEG ROI left pallidum |
| rsfmri_cor_ngd_cerc_scs_crcxrh | 6.47891724722752 | 4.43695987846297e-10 | 4.91956340004594 | 2.40095600781613e-06 | -4.219831124 | 0.000144653003956 | -3.815929445 | 0.000822149176723 | Average correlation between cingulo opercular network and ASEG ROI right cerebellum cortex |
| rsfmri_cor_ngd_cerc_scs_cderh | 13.11232037 | 5.05578543981807e-37 | 9.8338039491622 | 1.37256030038442e-21 | -6.988792998 | 5.3358818491610195e-11 | -4.211272323 | 0.000179384241866 | Average correlation between cingulo opercular network and ASEG ROI right caudate |
| rsfmri_cor_ngd_cerc_scs_aarh | 6.86027231794059 | 3.6484879267114597e-11 | 4.10987445322675 | 9.14411512666923e-05 | -5.501219626 | 4.02932555073446e-07 | -3.011178352 | 0.010803342505766 | Average correlation between cingulo opercular network and ASEG ROI right accumbens area |
| rsfmri_cor_ngd_df_scs_thprh | 9.16781373907557 | 6.11767514101355e-19 | 5.3048663906384 | 3.42334374236353e-07 | -5.344333987 | 8.9798840810266e-07 | -3.473670771 | 0.002677847754475 | Average correlation between default network and ASEG ROI right thalamus proper |
| rsfmri_cor_ngd_df_scs_ptrh | 8.65431835252135 | 4.89320109938804e-17 | 6.7040379051679 | 9.25297481101836e-11 | -4.28122348 | 0.000114024273165 | -3.787695686 | 0.000908129157007 | Average correlation between default network and ASEG ROI right putamen |
| rsfmri_cor_ngd_fopa_scs_pllh | 7.08341511486249 | 7.85997948039546e-12 | 4.66489687166488 | 8.00135157414073e-06 | -4.722130094 | 1.90732208399544e-05 | -2.955166947 | 0.012839598284449 | Average correlation between fronto parietal network and ASEG ROI left pallidum |
| rsfmri_cor_ngd_none_scs_hplh | 5.71197017096694 | 4.26615947633183e-08 | 6.52056113520612 | 2.94676435773322e-10 | -4.484001705 | 5.13354004874394e-05 | -5.400949962 | 7.49346641796584e-07 | Average correlation between none network and ASEG ROI left hippocampus |
| rsfmri_cor_ngd_none_scs_aalh | 13.0306993298546 | 1.3474699624308899e-36 | 13.1237966276305 | 4.94309509366583e-37 | -5.461873832 | 4.80104411988978e-07 | -5.548184569 | 3.47381196911762e-07 | Average correlation between none network and ASEG ROI left accumbens area |
| rsfmri_cor_ngd_none_scs_ptrh | 8.18826353701568 | 2.21834403220044e-15 | 10.6072942604576 | 7.302776471473931e-25 | -3.432981717 | 0.002356316547317 | -4.002491417 | 0.000398395743422 | Average correlation between none network and ASEG ROI right putamen |
| rsfmri_cor_ngd_rst_scs_crcxlh | 11.020848009174 | 9.810335795289289e-27 | 11.1146261310236 | 4.06407575700771e-27 | -5.478813261 | 4.46562367155505e-07 | -5.245450044 | 1.6637239528126e-06 | Average correlation between retrosplenial temporal network and ASEG ROI left cerebellum cortex |
| rsfmri_cor_ngd_rst_scs_cdelh | 13.4676785194888 | 6.48492355032882e-39 | 13.4401233389838 | 1.0336835876126e-38 | -7.154240197 | 1.6887653848068398e-11 | -7.32969854 | 5.52785577890593e-12 | Average correlation between retrosplenial temporal network and ASEG ROI left caudate |
| rsfmri_cor_ngd_rst_scs_aglh | 9.64353691026001 | 8.32858193215248e-21 | 9.7512054369237 | 2.88131119585182e-21 | -3.795006577 | 0.000698901556717 | -4.10631099 | 0.000270050543195 | Average correlation between retrosplenial temporal network and ASEG ROI left amygdala |
| rsfmri_cor_ngd_rst_scs_cderh | 13.8314609067491 | 6.61912976707855e-41 | 12.6774861519907 | 1.0271011830231e-34 | -4.1337475 | 0.000202432204904 | -5.108870024 | 3.24286473528311e-06 | Average correlation between retrosplenial temporal network and ASEG ROI right caudate |
| rsfmri_cor_ngd_rst_scs_ptrh | 14.1284314080213 | 1.4314787293526398e-42 | 12.3462072784679 | 4.94263173907364e-33 | -6.270495426 | 5.0224473062295805e-09 | -5.035728831 | 4.54515466967825e-06 | Average correlation between retrosplenial temporal network and ASEG ROI right putamen |
| rsfmri_cor_ngd_rst_scs_hprh | 16.8760007720256 | 2.42939350825604e-59 | 13.7514158095247 | 2.06471979654678e-40 | -6.465261258 | 1.46159917842734e-09 | -4.214872218 | 0.00017799253525 | Average correlation between retrosplenial temporal network and ASEG ROI right hippocampus |
| rsfmri_cor_ngd_smh_scs_thplh | 10.1317145391009 | 8.06311923545546e-23 | 9.65612730860782 | 6.90919019587866e-21 | -4.320593596 | 9.69383264226117e-05 | -3.621240875 | 0.001653583698476 | Average correlation between sensorimotor hand network and ASEG ROI left thalamus proper |
| rsfmri_cor_ngd_smh_scs_hplh | 15.7090414097633 | 6.61984745620407e-52 | 13.0294412042536 | 1.53567513019563e-36 | -4.387839171 | 7.5758027830146e-05 | -3.576048995 | 0.001927848547574 | Average correlation between sensorimotor hand network and ASEG ROI left hippocampus |
| rsfmri_cor_ngd_smh_scs_aglh | 7.17523004277123 | 4.2616584541078195e-12 | 6.82636448354236 | 4.1662677990363594e-11 | -4.409703827 | 7.06632522254936e-05 | -5.093968903 | 3.46773923473698e-06 | Average correlation between sensorimotor hand network and ASEG ROI left amygdala |
| rsfmri_cor_ngd_smh_scs_vtdclh | 12.2496178001877 | 1.35051389984228e-32 | 10.1418192845792 | 7.26000327456067e-23 | -7.606937336 | 6.80453711211041e-13 | -4.971929622 | 6.17783525961852e-06 | Average correlation between sensorimotor hand network and ASEG ROI left ventraldc |
| rsfmri_cor_ngd_smh_scs_plrh | 5.89711014938792 | 1.50731613348653e-08 | 4.98115487868512 | 1.76623626186392e-06 | -4.582503466 | 3.44974068339419e-05 | -3.536654821 | 0.00218210657192 | Average correlation between sensorimotor hand network and ASEG ROI right pallidum |
| rsfmri_cor_ngd_sa_scs_vtdcrh | 8.301986241 | 8.85185006991929e-16 | 8.80757810460684 | 1.23940096338459e-17 | -4.254131942 | 0.00012773797081 | -4.084441721 | 0.000291401520678 | Average correlation between salience network and ASEG ROI right ventraldc |
| rsfmri_cor_ngd_vta_scs_aglh | 11.482858948361 | 7.1911299074497e-29 | 9.15616066682011 | 6.0210410502423205e-19 | -5.910032825 | 4.00088373123869e-08 | -4.934579698 | 7.24848480258241e-06 | Average correlation between ventral attention network and ASEG ROI left amygdala |
| rsfmri_cor_ngd_vta_scs_aalh | 9.8283734939042 | 1.47106366301344e-21 | 9.39573760576484 | 7.30449124715791e-20 | -5.186930398 | 2.00933308355073e-06 | -4.068329869 | 0.000306008485383 | Average correlation between ventral attention network and ASEG ROI left accumbens area |
| rsfmri_cor_ngd_vs_scs_vtdclh | 7.1939638811208 | 3.74598703928676e-12 | 6.55302856316333 | 2.40081883492638e-10 | -4.407571323 | 7.08119854987313e-05 | -4.278101283 | 0.000137880014228 | Average correlation between visual network and ASEG ROI left ventraldc |
| rsfmri_cor_ngd_vs_scs_thprh | 14.0167973021006 | 6.07883987183806e-42 | 13.2096696126634 | 1.75507534806314e-37 | -7.278683359 | 7.28922161247643e-12 | -4.797558812 | 1.41111328265272e-05 | Average correlation between visual network and ASEG ROI right thalamus proper |
| rsfmri_cor_ngd_vs_scs_agrh | 9.18055219314644 | 5.49661966834705e-19 | 11.5087292389914 | 5.93166688071793e-29 | -3.627123655 | 0.001255975641101 | -4.989029727 | 5.71909697871828e-06 | Average correlation between visual network and ASEG ROI right amygdala |
| Baseline < 0, Development > 0 | | | | | | | | | |
| rsfmri_c_ngd_ad_ngd_cgc | -7.435935259 | 6.710335103634379e-13 | -6.82098227 | 4.2658093996924894e-11 | 3.31064855787927 | 0.003460723268899 | 2.91837186165777 | 0.014245915345756 | Average correlation between auditory network and cingulo opercular network |
| rsfmri_c_ngd_ad_ngd_ca | -10.62016712 | 6.1338852766779705e-25 | -9.638245954 | 8.005526609679491e-21 | 6.56338245371935 | 8.02879110184806e-10 | 5.80162818192871 | 8.36695830588515e-08 | Average correlation between auditory network and cingulo parietal network |
| rsfmri_c_ngd_ad_ngd_dt | -12.75501592 | 3.66161220919042e-35 | -12.32589549 | 6.1426844863655194e-33 | 6.59950633328696 | 6.71914599518976e-10 | 6.00872385867298 | 2.59882887900285e-08 | Average correlation between auditory network and default network |
| rsfmri_c_ngd_ad_ngd_fo | -17.65153209 | 1.90251472295727e-64 | -22.85015535 | 9.18610028296767e-103 | 8.60817517183085 | 4.00610766308965e-16 | 10.3214614580373 | 7.80215681023764e-23 | Average correlation between auditory network and fronto parietal network |
| rsfmri_c_ngd_ad_ngd_sa | -18.23430058 | 2.07028402935046e-68 | -18.51338986 | 3.22467098532403e-70 | 9.24450427387371 | 1.79028092730105e-18 | 11.2686520678899 | 7.50331225052487e-27 | Average correlation between auditory network and salience network |
| rsfmri_c_ngd_cgc_ngd_ad | -7.435935259 | 6.710335103634379e-13 | -6.82098227 | 4.2658093996924894e-11 | 3.31064855787927 | 0.003460723268899 | 2.91837186165777 | 0.014245915345756 | Average correlation between cingulo opercular network and auditory network |
| rsfmri_c_ngd_ca_ngd_ad | -10.62016712 | 6.1338852766779705e-25 | -9.638245954 | 8.005526609679491e-21 | 6.56338245371935 | 8.02879110184806e-10 | 5.80162818192871 | 8.36695830588515e-08 | Average correlation between cingulo parietal network and auditory network |
| rsfmri_c_ngd_ca_ngd_smh | -12.47403313 | 1.00735931218551e-33 | -9.109110125 | 9.047817842892471e-19 | 6.1600092471437 | 9.14893358968892e-09 | 6.03668287752718 | 2.2558776446726e-08 | Average correlation between cingulo parietal network and sensorimotor hand network |
| rsfmri_c_ngd_ca_ngd_smm | -11.49071887 | 6.65651584373882e-29 | -8.661460869 | 4.22950571540982e-17 | 6.53386306996946 | 9.4707217616409e-10 | 4.94129034790158 | 7.07597793385108e-06 | Average correlation between cingulo parietal network and sensorimotor mouth network |
| rsfmri_c_ngd_dt_ngd_ad | -12.75501592 | 3.66161220919042e-35 | -12.32589549 | 6.1426844863655194e-33 | 6.59950633328696 | 6.71914599518976e-10 | 6.00872385867298 | 2.59882887900285e-08 | Average correlation between default network and auditory network |
| rsfmri_c_ngd_dt_ngd_smh | -14.14532275 | 1.1615477891012399e-42 | -14.81136727 | 1.96429544481277e-46 | 8.18185157105957 | 9.28812853049441e-15 | 8.06204894736226 | 2.36948926980136e-14 | Average correlation between default network and sensorimotor hand network |
| rsfmri_c_ngd_dt_ngd_smm | -7.558476992 | 2.81640271420573e-13 | -8.215152631 | 1.6033432524499102e-15 | 3.95988018587501 | 0.000395142422184 | 4.71543188613952 | 2.0509362780225e-05 | Average correlation between default network and sensorimotor mouth network |
| rsfmri_c_ngd_dt_ngd_vta | -7.474361257 | 5.10306559199606e-13 | -8.533059636 | 1.2146932939460199e-16 | 3.66839716368463 | 0.001092663479956 | 3.63544047798244 | 0.001575839258636 | Average correlation between default network and ventral attention network |
| rsfmri_c_ngd_fo_ngd_ad | -17.65153209 | 1.90251472295727e-64 | -22.85015535 | 9.18610028296767e-103 | 8.60817517183085 | 4.00610766308965e-16 | 10.3214614580373 | 7.80215681023764e-23 | Average correlation between fronto parietal network and auditory network |
| rsfmri_c_ngd_fo_ngd_smh | -22.61770566 | 2.34147288669735e-101 | -23.72521703 | 7.05089976219023e-110 | 10.3822879005092 | 4.4193225364129204e-23 | 9.87002860764716 | 4.9645860551839804e-21 | Average correlation between fronto parietal network and sensorimotor hand network |
| rsfmri_c_ngd_fo_ngd_smm | -13.3762499 | 1.97461139556729e-38 | -15.87374972 | 7.55857983529618e-53 | 6.56714717926877 | 8.02879110184806e-10 | 8.27104497216203 | 4.8573117434650405e-15 | Average correlation between fronto parietal network and sensorimotor mouth network |
| rsfmri_c_ngd_fo_ngd_vta | -4.62513171 | 1.09152839972059e-05 | -5.348608757 | 2.71664987573368e-07 | 2.73802944368877 | 0.016436303628062 | 3.24059761049526 | 0.005470585126754 | Average correlation between fronto parietal network and ventral attention network |
| rsfmri_c_ngd_fo_ngd_vs | -5.582617565 | 8.78619014914633e-08 | -5.114841113 | 9.17976552888048e-07 | 4.64903250962989 | 2.57609968759135e-05 | 3.42009418683537 | 0.003129825888455 | Average correlation between fronto parietal network and visual network |
| rsfmri_c_ngd_smh_ngd_ca | -12.47403313 | 1.00735931218551e-33 | -9.109110125 | 9.047817842892471e-19 | 6.1600092471437 | 9.14893358968892e-09 | 6.03668287752718 | 2.2558776446726e-08 | Average correlation between sensorimotor hand network and cingulo parietal network |
| rsfmri_c_ngd_smh_ngd_dt | -14.14532275 | 1.1615477891012399e-42 | -14.81136727 | 1.96429544481277e-46 | 8.18185157105957 | 9.28812853049441e-15 | 8.06204894736226 | 2.36948926980136e-14 | Average correlation between sensorimotor hand network and default network |
| rsfmri_c_ngd_smh_ngd_fo | -22.61770566 | 2.34147288669735e-101 | -23.72521703 | 7.05089976219023e-110 | 10.3822879005092 | 4.4193225364129204e-23 | 9.87002860764716 | 4.9645860551839804e-21 | Average correlation between sensorimotor hand network and fronto parietal network |
| rsfmri_c_ngd_smh_ngd_sa | -18.8951899 | 4.81565233623175e-73 | -17.86095263 | 9.66350810914392e-66 | 8.59802616726085 | 4.07407466064157e-16 | 9.65565300123059 | 3.40058907466096e-20 | Average correlation between sensorimotor hand network and salience network |
| rsfmri_c_ngd_smm_ngd_ca | -11.49071887 | 6.65651584373882e-29 | -8.661460869 | 4.22950571540982e-17 | 6.53386306996946 | 9.4707217616409e-10 | 4.94129034790158 | 7.07597793385108e-06 | Average correlation between sensorimotor mouth network and cingulo parietal network |
| rsfmri_c_ngd_smm_ngd_dt | -7.558476992 | 2.81640271420573e-13 | -8.215152631 | 1.6033432524499102e-15 | 3.95988018587501 | 0.000395142422184 | 4.71543188613952 | 2.0509362780225e-05 | Average correlation between sensorimotor mouth network and default network |
| rsfmri_c_ngd_smm_ngd_fo | -13.3762499 | 1.97461139556729e-38 | -15.87374972 | 7.55857983529618e-53 | 6.56714717926877 | 8.02879110184806e-10 | 8.27104497216203 | 4.8573117434650405e-15 | Average correlation between sensorimotor mouth network and fronto parietal network |
| rsfmri_c_ngd_smm_ngd_sa | -15.27306949 | 2.89157948825647e-49 | -12.85451594 | 1.24272241952234e-35 | 6.65065533974237 | 4.936527596495281e-10 | 7.02545131024108 | 4.1124918561254e-11 | Average correlation between sensorimotor mouth network and salience network |
| rsfmri_c_ngd_sa_ngd_ad | -18.23430058 | 2.07028402935046e-68 | -18.51338986 | 3.22467098532403e-70 | 9.24450427387371 | 1.79028092730105e-18 | 11.2686520678899 | 7.50331225052487e-27 | Average correlation between salience network and auditory network |
| rsfmri_c_ngd_sa_ngd_smh | -18.8951899 | 4.81565233623175e-73 | -17.86095263 | 9.66350810914392e-66 | 8.59802616726085 | 4.07407466064157e-16 | 9.65565300123059 | 3.40058907466096e-20 | Average correlation between salience network and sensorimotor hand network |
| rsfmri_c_ngd_sa_ngd_smm | -15.27306949 | 2.89157948825647e-49 | -12.85451594 | 1.24272241952234e-35 | 6.65065533974237 | 4.936527596495281e-10 | 7.02545131024108 | 4.1124918561254e-11 | Average correlation between salience network and sensorimotor mouth network |
| rsfmri_c_ngd_sa_ngd_vta | -11.34504415 | 3.14921891355913e-28 | -11.1044231 | 4.45190239578017e-27 | 3.54132870250238 | 0.001665908847132 | 4.1216461580532 | 0.000254753321946 | Average correlation between salience network and ventral attention network |
| rsfmri_c_ngd_vta_ngd_dt | -7.474361257 | 5.10306559199606e-13 | -8.533059636 | 1.2146932939460199e-16 | 3.66839716368463 | 0.001092663479956 | 3.63544047798244 | 0.001575839258636 | Average correlation between ventral attention network and default network |
| rsfmri_c_ngd_vta_ngd_fo | -4.62513171 | 1.09152839972059e-05 | -5.348608757 | 2.71664987573368e-07 | 2.73802944368877 | 0.016436303628062 | 3.24059761049526 | 0.005470585126754 | Average correlation between ventral attention network and fronto parietal network |
| rsfmri_c_ngd_vta_ngd_sa | -11.34504415 | 3.14921891355913e-28 | -11.1044231 | 4.45190239578017e-27 | 3.54132870250238 | 0.001665908847132 | 4.1216461580532 | 0.000254753321946 | Average correlation between ventral attention network and salience network |
| rsfmri_c_ngd_vs_ngd_fo | -5.582617565 | 8.78619014914633e-08 | -5.114841113 | 9.17976552888048e-07 | 4.64903250962989 | 2.57609968759135e-05 | 3.42009418683537 | 0.003129825888455 | Average correlation between visual network and fronto parietal network |
| rsfmri_var_cortgordon_gp243rh | -4.08876939 | 0.000112499918524 | -2.737625963 | 0.01047634056003 | 3.47110561758267 | 0.002056664750741 | 3.86580524439931 | 0.000681312883858 | Temporal variance in right hemisphere cortical Gordon parcel 243 (insula _R) |
| rsfmri_var_scs_cbcortexlh | -6.448560873 | 5.37942518191137e-10 | -5.755593376 | 2.89120755337902e-08 | 3.65943352366461 | 0.001125576438426 | 3.26243461232171 | 0.005206361057026 | Temporal variance in ASEG ROI left cerebral cortex |
| rsfmri_cor_ngd_au_scs_crcxlh | -18.46944704 | 4.9760285456213095e-70 | -21.16894657 | 1.44589275149695e-89 | 8.18448191335616 | 9.28812853049441e-15 | 8.23523110062437 | 6.33610796664753e-15 | Average correlation between auditory network and ASEG ROI left cerebellum cortex |
| rsfmri_cor_ngd_au_scs_aalh | -24.42313046 | 3.18551777527115e-116 | -24.60667443 | 3.66563689189692e-117 | 9.13969047961023 | 4.40621204482127e-18 | 8.9442219592708 | 1.8912269445389202e-17 | Average correlation between auditory network and ASEG ROI left accumbens area |
| rsfmri_cor_ngd_au_scs_vtdclh | -19.37425251 | 1.78328860261667e-76 | -20.68693785 | 6.10105149511471e-86 | 7.65113868914491 | 5.07185060378853e-13 | 7.27288243062146 | 8.18486540066232e-12 | Average correlation between auditory network and ASEG ROI left ventraldc |
| rsfmri_cor_ngd_au_scs_cderh | -11.10199197 | 4.18273659612256e-27 | -13.08872072 | 7.51938765844797e-37 | 3.25434054743842 | 0.004132859580981 | 5.52178264821096 | 3.97052245722701e-07 | Average correlation between auditory network and ASEG ROI right caudate |
| rsfmri_cor_ngd_au_scs_ptrh | -30.00436431 | 3.20021074970269e-166 | -28.96989924 | 1.6719639522236002e-155 | 13.7522868988699 | 3.75195885971459e-39 | 13.7451698861938 | 9.83857471142979e-39 | Average correlation between auditory network and ASEG ROI right putamen |
| rsfmri_cor_ngd_au_scs_plrh | -13.46238945 | 6.85170476859574e-39 | -13.33914579 | 3.56636214762185e-38 | 6.84537657099448 | 1.3903617428632401e-10 | 6.21383478928173 | 7.892856767504941e-09 | Average correlation between auditory network and ASEG ROI right pallidum |
| rsfmri_cor_ngd_cerc_scs_cdelh | -29.46266137 | 3.2479790582759104e-161 | -28.10947823 | 1.1279661900394799e-147 | 10.2562799808701 | 1.4047782364737398e-22 | 11.257101445958 | 7.842004667502259e-27 | Average correlation between cingulo opercular network and ASEG ROI left caudate |
| rsfmri_cor_ngd_cerc_scs_ptlh | -27.7808029 | 9.653163557757459e-146 | -27.04751253 | 3.42753615779881e-138 | 10.4119544099525 | 3.6670029551827e-23 | 10.6879631528835 | 2.23948359635509e-24 | Average correlation between cingulo opercular network and ASEG ROI left putamen |
| rsfmri_cor_ngd_cerc_scs_bs | -13.09884423 | 5.90318698999406e-37 | -14.66872129 | 1.28682648151934e-45 | 3.70509641778052 | 0.000959180588137 | 5.45537834194145 | 5.61344109509197e-07 | Average correlation between cingulo opercular network and ASEG ROI brain stem |
| rsfmri_cor_ngd_cerc_scs_hplh | -9.512833459 | 2.73386907623109e-20 | -9.710217304 | 4.21321141201548e-21 | 3.57372075735855 | 0.001510135852115 | 3.74991376658559 | 0.001048337235491 | Average correlation between cingulo opercular network and ASEG ROI left hippocampus |
| rsfmri_cor_ngd_cerc_scs_aglh | -28.9889053 | 7.723205851451011e-157 | -31.32767019 | 2.1339806276892496e-177 | 11.6215548443734 | 1.58691606900594e-28 | 13.7521520124457 | 9.83857471142979e-39 | Average correlation between cingulo opercular network and ASEG ROI left amygdala |
| rsfmri_cor_ngd_cerc_scs_aalh | -9.99282059 | 3.09803195298528e-22 | -13.97074113 | 1.24703884913881e-41 | 3.14867809038035 | 0.005600075268431 | 7.2168684852541 | 1.1350881982737699e-11 | Average correlation between cingulo opercular network and ASEG ROI left accumbens area |
| rsfmri_cor_ngd_cerc_scs_hprh | -35.04227009 | 2.60725777606862e-215 | -34.71399185 | 5.08604608964313e-210 | 12.3999196112313 | 2.70236520522956e-32 | 12.777813468629 | 4.4658636230573395e-34 | Average correlation between cingulo opercular network and ASEG ROI right hippocampus |
| rsfmri_cor_ngd_cerc_scs_agrh | -23.91478204 | 5.76441165888523e-112 | -23.58968093 | 8.95206406841524e-109 | 8.26407001477049 | 5.540806446943311e-15 | 9.77779971881817 | 1.15194608840746e-20 | Average correlation between cingulo opercular network and ASEG ROI right amygdala |
| rsfmri_cor_ngd_cerc_scs_vtdcrh | -15.30036923 | 2.02923938266333e-49 | -17.01327561 | 4.4303948281405404e-60 | 2.9550117862342 | 0.009652679019559 | 5.04899909323176 | 4.28846875051833e-06 | Average correlation between cingulo opercular network and ASEG ROI right ventraldc |
| rsfmri_cor_ngd_copa_scs_thplh | -23.09253791 | 3.61541690129034e-105 | -26.81068987 | 4.06658223133665e-136 | 7.68952012910929 | 3.95184042496616e-13 | 11.0206757384624 | 7.89840862636061e-26 | Average correlation between cingulo parietal network and ASEG ROI left thalamus proper |
| rsfmri_cor_ngd_copa_scs_vtdclh | -22.18171269 | 7.15615246477809e-98 | -21.85068669 | 7.59054908874389e-95 | 6.1619544772583 | 9.14893358968892e-09 | 7.06605711331185 | 3.2017250023689495e-11 | Average correlation between cingulo parietal network and ASEG ROI left ventraldc |
| rsfmri_cor_ngd_copa_scs_crcxrh | -18.24532045 | 1.81316139389258e-68 | -18.4047459 | 1.82276604011314e-69 | 4.68358012439969 | 2.25879385292157e-05 | 6.55053251569111 | 9.68343150763591e-10 | Average correlation between cingulo parietal network and ASEG ROI right cerebellum cortex |
| rsfmri_cor_ngd_copa_scs_plrh | -20.63208732 | 8.36251437532855e-86 | -21.72244408 | 7.4771265190201e-94 | 10.3093622767548 | 8.702337768212771e-23 | 11.0893765035693 | 4.07369356925579e-26 | Average correlation between cingulo parietal network and ASEG ROI right pallidum |
| rsfmri_cor_ngd_copa_scs_hprh | -7.862896686 | 2.86125416973118e-14 | -10.20967283 | 3.81322046427162e-23 | 3.37932740886212 | 0.00277062626951 | 5.50689224190669 | 4.26318359837728e-07 | Average correlation between cingulo parietal network and ASEG ROI right hippocampus |
| rsfmri_cor_ngd_df_scs_ptlh | -20.35239115 | 1.0799566313728399e-83 | -20.63134605 | 1.49660539603853e-85 | 7.86327150234142 | 1.08281146429944e-13 | 9.17648280139819 | 2.49499573219979e-18 | Average correlation between default network and ASEG ROI left putamen |
| rsfmri_cor_ngd_df_scs_pllh | -17.81461798 | 1.5216672019534901e-65 | -17.94096483 | 2.83489304615464e-66 | 7.23142190935113 | 1.0068191934888099e-11 | 8.13117640728589 | 1.43073807188332e-14 | Average correlation between default network and ASEG ROI left pallidum |
| rsfmri_cor_ngd_df_scs_aalh | -17.89986529 | 4.03336964558241e-66 | -16.64843859 | 1.01602943657097e-57 | 8.31254135401736 | 3.985629204475221e-15 | 5.88650636560751 | 5.34457467335348e-08 | Average correlation between default network and ASEG ROI left accumbens area |
| rsfmri_cor_ngd_df_scs_agrh | -16.55571982 | 2.89758243997319e-57 | -14.4620281 | 2.0236114820214601e-44 | 5.54742198648277 | 3.14310402304995e-07 | 5.27444437247145 | 1.47456662130622e-06 | Average correlation between default network and ASEG ROI right amygdala |
| rsfmri_cor_ngd_df_scs_aarh | -16.92449109 | 1.2031967700478001e-59 | -14.67247784 | 1.23808573466264e-45 | 5.91576645729489 | 3.91303739778341e-08 | 4.49566587047554 | 5.57672068412674e-05 | Average correlation between default network and ASEG ROI right accumbens area |
| rsfmri_cor_ngd_dsa_scs_vtdcrh | -9.118713118 | 9.421244307360411e-19 | -10.0011842 | 2.8124315929969197e-22 | 3.1703609733758 | 0.005280239353199 | 3.46990871758413 | 0.002694250764928 | Average correlation between dorsal attention network and ASEG ROI right ventraldc |
| rsfmri_cor_ngd_fopa_scs_hplh | -4.39161351 | 3.11821550074911e-05 | -4.728620705 | 5.92657023713149e-06 | 4.21088284314999 | 0.000148467233976 | 4.7292453484263 | 1.95436456717519e-05 | Average correlation between fronto parietal network and ASEG ROI left hippocampus |
| rsfmri_cor_ngd_fopa_scs_vtdclh | -9.67747036 | 6.10127271451829e-21 | -9.580838495 | 1.35000247118478e-20 | 3.05427194923505 | 0.007315940617399 | 4.59322951414081 | 3.58878888836721e-05 | Average correlation between fronto parietal network and ASEG ROI left ventraldc |
| rsfmri_cor_ngd_fopa_scs_aarh | -14.81572028 | 1.5561908487040401e-46 | -11.80668649 | 2.2234806141755e-30 | 6.18151722437533 | 8.3181081885382e-09 | 4.42998809211996 | 7.29476693544941e-05 | Average correlation between fronto parietal network and ASEG ROI right accumbens area |
| rsfmri_cor_ngd_none_scs_ptlh | -22.40259133 | 1.24208061016899e-99 | -21.02573954 | 1.67759560366807e-88 | 10.4731156190506 | 2.10342809605882e-23 | 9.3311308046592 | 6.37656487135654e-19 | Average correlation between none network and ASEG ROI left putamen |
| rsfmri_cor_ngd_none_scs_thprh | -26.81967665 | 4.30462760755668e-137 | -23.9218569 | 1.86048273857689e-111 | 10.7753216248038 | 1.17420514225624e-24 | 9.45868760145454 | 2.05008007087413e-19 | Average correlation between none network and ASEG ROI right thalamus proper |
| rsfmri_cor_ngd_none_scs_cderh | -21.48919689 | 2.1419217100313002e-92 | -19.35223839 | 3.97121505048112e-76 | 7.776048213 | 2.08078396016892e-13 | 7.10469795727743 | 2.4810549841051898e-11 | Average correlation between none network and ASEG ROI right caudate |
| rsfmri_cor_ngd_none_scs_agrh | -16.94263973 | 9.2869679147784e-60 | -16.6440279 | 1.0690674316441899e-57 | 7.61939116003004 | 6.31968246217463e-13 | 5.23855317754295 | 1.6875389854543e-06 | Average correlation between none network and ASEG ROI right amygdala |
| rsfmri_cor_ngd_rst_scs_bs | -23.15102025 | 1.24576429235105e-105 | -22.0430072 | 2.3830958803191e-96 | 7.67919370853992 | 4.18451959772915e-13 | 6.7140372376511 | 3.37416428411894e-10 | Average correlation between retrosplenial temporal network and ASEG ROI brain stem |
| rsfmri_cor_ngd_rst_scs_hplh | -17.49001993 | 2.28300366546237e-63 | -16.94398743 | 1.2440427373008201e-59 | 6.87495062498827 | 1.1544382706464101e-10 | 5.117147825 | 3.13944484145841e-06 | Average correlation between retrosplenial temporal network and ASEG ROI left hippocampus |
| rsfmri_cor_ngd_rst_scs_vtdclh | -9.110947459 | 1.00253758511186e-18 | -10.24390887 | 2.7409045846065804e-23 | 4.02369017624295 | 0.000311835938903 | 5.24160632221873 | 1.67904133656204e-06 | Average correlation between retrosplenial temporal network and ASEG ROI left ventraldc |
| rsfmri_cor_ngd_rst_scs_crcxrh | -30.66755546 | 2.02777262909905e-172 | -29.24238738 | 5.628135459583779e-158 | 13.8468267731444 | 1.48429920406694e-39 | 13.4554022979303 | 1.92062390667195e-37 | Average correlation between retrosplenial temporal network and ASEG ROI right cerebellum cortex |
| rsfmri_cor_ngd_rst_scs_thprh | -15.25755809 | 3.54292609768547e-49 | -14.32786737 | 1.18459086521843e-43 | 6.72651288207286 | 3.06693131342096e-10 | 6.24777416847498 | 6.4765668647947605e-09 | Average correlation between retrosplenial temporal network and ASEG ROI right thalamus proper |
| rsfmri_cor_ngd_rst_scs_vtdcrh | -28.44703112 | 7.75230994872208e-152 | -26.62349647 | 1.72846042635597e-134 | 9.35560797518437 | 7.123198047107531e-19 | 10.7771767083558 | 9.457154369160751e-25 | Average correlation between retrosplenial temporal network and ASEG ROI right ventraldc |
| rsfmri_cor_ngd_smh_scs_crcxlh | -27.37611509 | 4.36997055282703e-142 | -26.17864016 | 1.32828701945428e-130 | 10.5242701390481 | 1.33649418210582e-23 | 10.3486281714149 | 6.57855652174715e-23 | Average correlation between sensorimotor hand network and ASEG ROI left cerebellum cortex |
| rsfmri_cor_ngd_smh_scs_cdelh | -10.88469227 | 4.03957979624413e-26 | -11.08909757 | 5.19376628641494e-27 | 4.22799958904652 | 0.000140481608515 | 5.04914135257995 | 4.28846875051833e-06 | Average correlation between sensorimotor hand network and ASEG ROI left caudate |
| rsfmri_cor_ngd_smh_scs_ptlh | -12.76869174 | 3.1711362225608203e-35 | -10.89944099 | 3.63837117249173e-26 | 4.42974051348757 | 6.54618203231157e-05 | 3.05219732214752 | 0.009621912417368 | Average correlation between sensorimotor hand network and ASEG ROI left putamen |
| rsfmri_cor_ngd_smh_scs_pllh | -30.47406124 | 1.2337545627152001e-170 | -33.3420934 | 1.16347100046415e-196 | 12.4418451565796 | 1.85794325712847e-32 | 13.586986148066 | 4.88865136924516e-38 | Average correlation between sensorimotor hand network and ASEG ROI left pallidum |
| rsfmri_cor_ngd_smh_scs_bs | -11.13826514 | 2.86571904883405e-27 | -14.68126393 | 1.1132965184478001e-45 | 4.38575191811214 | 7.59158165436322e-05 | 6.41726125619873 | 2.2696062389335903e-09 | Average correlation between sensorimotor hand network and ASEG ROI brain stem |
| rsfmri_cor_ngd_smh_scs_cderh | -36.10585617 | 5.9247038119408196e-226 | -36.47106798 | 6.8756192497421e-227 | 12.6095245969583 | 2.9203884833298e-33 | 12.1631454266061 | 5.345335693250221e-31 | Average correlation between sensorimotor hand network and ASEG ROI right caudate |
| rsfmri_cor_ngd_smh_scs_ptrh | -24.16972242 | 4.28386747279825e-114 | -25.68129957 | 2.54990227541647e-126 | 7.99869969931581 | 3.9191405005154e-14 | 10.2095631753201 | 2.15762897164706e-22 | Average correlation between sensorimotor hand network and ASEG ROI right putamen |
| rsfmri_cor_ngd_smh_scs_hprh | -14.77742755 | 2.5872533877856002e-46 | -16.29506548 | 1.77426342410019e-55 | 3.19628637832884 | 0.004912468848183 | 5.27148313354344 | 1.48064279136941e-06 | Average correlation between sensorimotor hand network and ASEG ROI right hippocampus |
| rsfmri_cor_ngd_smh_scs_aarh | -22.74649276 | 2.26401668473872e-102 | -25.9499025 | 1.24801858278455e-128 | 8.31605461569608 | 3.985629204475221e-15 | 10.1443801685841 | 3.9235615107720002e-22 | Average correlation between sensorimotor hand network and ASEG ROI right accumbens area |
| rsfmri_cor_ngd_smm_scs_hplh | -21.11368214 | 1.70524254412089e-89 | -20.70271267 | 4.75077677083923e-86 | 5.70412077776082 | 1.31314683973708e-07 | 7.21810477355904 | 1.1350881982737699e-11 | Average correlation between sensorimotor mouth network and ASEG ROI left hippocampus |
| rsfmri_cor_ngd_smm_scs_aglh | -17.41570013 | 7.11323724946634e-63 | -17.94662578 | 2.63745684618029e-66 | 4.92202555783426 | 7.26586421709451e-06 | 6.93780743207129 | 7.447257430244219e-11 | Average correlation between sensorimotor mouth network and ASEG ROI left amygdala |
| rsfmri_cor_ngd_smm_scs_thprh | -19.23555621 | 1.77623188298917e-75 | -18.80873608 | 2.76639340573273e-72 | 8.85282651468096 | 5.3007354722557305e-17 | 7.87820288318356 | 9.62663868589175e-14 | Average correlation between sensorimotor mouth network and ASEG ROI right thalamus proper |
| rsfmri_cor_ngd_smm_scs_cderh | -8.501629126 | 1.75080230402886e-16 | -7.963961339 | 1.17754514668532e-14 | 2.99710018793202 | 0.008573294927493 | 3.8231978471053 | 0.000804011615651 | Average correlation between sensorimotor mouth network and ASEG ROI right caudate |
| rsfmri_cor_ngd_sa_scs_crcxlh | -17.63121437 | 2.56624601555486e-64 | -16.46397743 | 1.50019630109683e-56 | 4.55716974264777 | 3.82464447658683e-05 | 6.20380092814777 | 8.278795348424741e-09 | Average correlation between salience network and ASEG ROI left cerebellum cortex |
| rsfmri_cor_ngd_sa_scs_thplh | -16.91662595 | 1.33380739341558e-59 | -17.09468906 | 1.30824252537256e-60 | 5.43357635443958 | 5.55737555529494e-07 | 7.60218384178101 | 7.48412107035167e-13 | Average correlation between salience network and ASEG ROI left thalamus proper |
| rsfmri_cor_ngd_sa_scs_aalh | -8.891284142 | 6.57602818934824e-18 | -11.00053233 | 1.2914439147601099e-26 | 2.88126009993953 | 0.01157754471431 | 3.25393531098091 | 0.005305453890598 | Average correlation between salience network and ASEG ROI left accumbens area |
| rsfmri_cor_ngd_vta_scs_cdelh | -11.85019772 | 1.2546249148408298e-30 | -11.65890022 | 1.14379871160334e-29 | 5.49202875274326 | 4.19438187497619e-07 | 3.91386254542298 | 0.000571725226254 | Average correlation between ventral attention network and ASEG ROI left caudate |
| rsfmri_cor_ngd_vta_scs_vtdclh | -6.592956515 | 2.11832815816664e-10 | -6.306817301 | 1.1235165247275e-09 | 5.70566513314325 | 1.31314683973708e-07 | 5.82338742171502 | 7.55972757975049e-08 | Average correlation between ventral attention network and ASEG ROI left ventraldc |
| rsfmri_cor_ngd_vs_scs_hplh | -22.94659395 | 5.4691902154118e-104 | -22.52886742 | 3.43679624030242e-100 | 11.2578588086706 | 7.74739268488439e-27 | 10.2336288595856 | 1.77898385084899e-22 | Average correlation between visual network and ASEG ROI left hippocampus |
| rsfmri_cor_ngd_vs_scs_plrh | -25.75453273 | 1.1563833211163e-127 | -24.21734671 | 6.63306485755307e-114 | 10.5751856075345 | 8.53137162854589e-24 | 10.0271671044439 | 1.1808001678629002e-21 | Average correlation between visual network and ASEG ROI right pallidum |
| rsfmri_cor_ngd_vs_scs_hprh | -21.4141906 | 8.029329036381739e-92 | -19.70237373 | 1.14170323206429e-78 | 7.98649452418233 | 4.21566684604673e-14 | 8.00201387415452 | 3.7272587658590204e-14 | Average correlation between visual network and ASEG ROI right hippocampus |

**Table S2. Significant clinical features and directions to label from two independent samples.**

| Feature | Direction | Source |
| --- | --- | --- |
| demo_race_a_p___10 | Negative for label 1 | abcd-general |
| demo_race_a_p___11 | Positive for label 1 | abcd-general |
| demo_ethn_v2 | Negative for label 1 | abcd-general |
| demo_prnt_age_v2 | Negative for label 1 | abcd-general |
| demo_prnt_race_a_v2___10 | Negative for label 1 | abcd-general |
| demo_prnt_race_a_v2___11 | Positive for label 1 | abcd-general |
| demo_prnt_ethn_v2 | Negative for label 1 | abcd-general |
| demo_prnt_marital_v2 | Positive for label 1 | abcd-general |
| demo_prnt_ed_v2 | Negative for label 1 | abcd-general |
| demo_prnt_prtnr_v2 | Positive for label 1 | abcd-general |
| demo_comb_income_v2 | Negative for label 1 | abcd-general |
| demo_fam_exp2_v2 | Positive for label 1 | abcd-general |
| demo_fam_exp3_v2 | Positive for label 1 | abcd-general |
| fam_roster_2c_v2 | Positive for label 1 | abcd-general |
| demo_prnt_age_v2_l | Negative for label 1 | abcd-general |
| race_ethnicity | Positive for label 1 | abcd-general |
| scrn_hr_smoke | Positive for label 1 | abcd-general |
| nihtbx_picvocab_uncorrected | Negative for label 1 | neurocognition |
| nihtbx_picvocab_agecorrected | Negative for label 1 | neurocognition |
| nihtbx_cardsort_uncorrected | Negative for label 1 | neurocognition |
| nihtbx_reading_uncorrected | Negative for label 1 | neurocognition |
| nihtbx_fluidcomp_uncorrected | Negative for label 1 | neurocognition |
| nihtbx_cryst_uncorrected | Negative for label 1 | neurocognition |
| nihtbx_cryst_agecorrected | Negative for label 1 | neurocognition |
| nihtbx_totalcomp_uncorrected | Negative for label 1 | neurocognition |
| nihtbx_totalcomp_agecorrected | Negative for label 1 | neurocognition |
| nihtbx_picvocab_theta | Negative for label 1 | neurocognition |
| nihtbx_cardsort_cs | Negative for label 1 | neurocognition |
| nihtbx_reading_theta | Negative for label 1 | neurocognition |
| pea_ravlt_sd_trial_ii_tc | Negative for label 1 | neurocognition |
| snellen_va_y | Negative for label 1 | neurocognition |
| pea_wiscv_tss | Negative for label 1 | neurocognition |
| devhx_3_p | Negative for label 1 | physical-health |
| devhx_6_p | Negative for label 1 | physical-health |
| devhx_8_tobacco | Positive for label 1 | physical-health |
| devhx_8_marijuana | Positive for label 1 | physical-health |
| devhx_9_tobacco | Positive for label 1 | physical-health |
| devhx_ss_8_marijuana_amt_p | Positive for label 1 | physical-health |
| caff_24 | Positive for label 1 | physical-health |
| medhx_ss_4b_p | Positive for label 1 | physical-health |
| pds_1_p | Positive for label 1 | physical-health |
| pds_2_p | Positive for label 1 | physical-health |
| sai_p_activities___15 | Negative for label 1 | physical-health |
| sai_p_activities___23 | Negative for label 1 | physical-health |
| sai_p_activities___29 | Positive for label 1 | physical-health |
| sai_ss_soc_nyr_p | Negative for label 1 | physical-health |
| sai_ss_soc_tspent_p | Negative for label 1 | physical-health |
| sai_ss_music_nyr_p | Negative for label 1 | physical-health |
| sai_ss_music_nmonth_p | Negative for label 1 | physical-health |
| sleepdisturb1_p | Positive for label 1 | physical-health |
| anthroweight1lb | Positive for label 1 | physical-health |
| anthroweight2lb | Positive for label 1 | physical-health |
| anthroweightcalc | Positive for label 1 | physical-health |
| anthro_waist_cm | Positive for label 1 | physical-health |
| fam_enviro9r_p | Negative for label 1 | culture-environment |
| mex_american1_p | Positive for label 1 | culture-environment |
| mex_american2_p | Positive for label 1 | culture-environment |
| mex_american3_p | Positive for label 1 | culture-environment |
| mex_american4_p | Positive for label 1 | culture-environment |
| mex_american6_p | Positive for label 1 | culture-environment |
| mex_american10_p | Positive for label 1 | culture-environment |
| mex_american11_p | Positive for label 1 | culture-environment |
| mex_american12_p | Positive for label 1 | culture-environment |
| mex_american15_p | Positive for label 1 | culture-environment |
| mex_american18_p | Positive for label 1 | culture-environment |
| mex_american20_p | Positive for label 1 | culture-environment |
| mex_american22_p | Positive for label 1 | culture-environment |
| mex_american25_p | Positive for label 1 | culture-environment |
| mex_american27_p | Positive for label 1 | culture-environment |
| mex_american28_p | Positive for label 1 | culture-environment |
| macv_p_ss_fs | Positive for label 1 | culture-environment |
| macv_p_ss_fo | Positive for label 1 | culture-environment |
| macv_p_ss_fr | Positive for label 1 | culture-environment |
| macv_p_ss_r | Positive for label 1 | culture-environment |
| meim_4_p | Negative for label 1 | culture-environment |
| meim_5_p | Negative for label 1 | culture-environment |
| meim_6_p | Negative for label 1 | culture-environment |
| meim_ethnic_id_p | Positive for label 1 | culture-environment |
| meim_4f_p | Positive for label 1 | culture-environment |
| meim_5f_p | Positive for label 1 | culture-environment |
| meim_6f_p | Positive for label 1 | culture-environment |
| neighborhood1r_p | Negative for label 1 | culture-environment |
| neighborhood2r_p | Negative for label 1 | culture-environment |
| neighborhood3r_p | Negative for label 1 | culture-environment |
| nsc_p_ss_mean_3_items | Negative for label 1 | culture-environment |
| crpbi_caregiver2_y | Positive for label 1 | culture-environment |
| neighborhood_crime_y | Negative for label 1 | culture-environment |
| su_risk_p_1 | Negative for label 1 | substance-use |
| parent_rules_q7 | Negative for label 1 | substance-use |
| su_y_hair_status_y | Positive for label 1 | substance-use |
| pls1_y_caf | Positive for label 1 | substance-use |
| screentime1_p_hours | Positive for label 1 | novel-technologies |
| screentime2_p_hours | Positive for label 1 | novel-technologies |
| screen2_wkdy_y | Positive for label 1 | novel-technologies |
| screen4_wkdy_y | Positive for label 1 | novel-technologies |
| screen5_wkdy_y | Positive for label 1 | novel-technologies |
| screen10_wknd_y | Positive for label 1 | novel-technologies |
| screen11_wknd_y | Positive for label 1 | novel-technologies |
| screen13_y | Positive for label 1 | novel-technologies |
| screen14_y | Positive for label 1 | novel-technologies |
| stq_y_ss_weekday | Positive for label 1 | novel-technologies |
| stq_y_ss_weekend | Positive for label 1 | novel-technologies |
| asr_q29_p | Positive for label 1 | mental-health |
| fhx_3ha_p | Positive for label 1 | mental-health |
| fhx_3hb_p | Positive for label 1 | mental-health |
| famhx_ss_momdad_trb_p | Positive for label 1 | mental-health |
| bisbas14_y | Positive for label 1 | mental-health |

**Table S3. The selected MRI measures**

| Modalities | Number | Measures |
| --- | --- | --- |
| sMRI | 258 | Volume of subcortical ROIs |
|  |  | Cortical thickness and cortical area of cortical ROIs |
| rsfMRI | 856 | Correlation within and between cortical networks |
|  |  | Correlation between cortical networks and subcortical ROIs |
|  |  | Temporal variance in subcortical ROIs, cortical ROIs and in Gordon parcels |
| dMRI | 84 | Fractional anisotropy (FA) within DTI atlas tract |
|  |  | Fiber tract volume within DTI atlas tract |

**Table S4. Number of demographic features included**

| Domains | Number Examples |
| --- | --- |
| General information | 134 |
| Neurocognition | 162 |
| Physical-health | 420 |
| Gender-identity-sexual-health | 13 |
| Culture-environment | 180 |
| Substance-use | 84 |
| Novel-technologies | 25 |
| Mental-health | 1305 |

**Table S5. Demographic comparation between included individuals and excluded individuals**

| **variable** | **type** | **included_n** | **excluded_n** | **included_mean** | **included_sd** | **excluded_mean** | **excluded_sd** | **effect_size** | **effect_size_type** | **p_fdr** |
| --- | --- | --- | --- | --- | --- | --- | --- | --- | --- | --- |
| **race_ethnicity** | categorical | 5333 | 3634 |  |  |  |  | 0.10284969174884316 | Cramers_V | 4.530233735232611e-18 |
| **demo_comb_income_v2** | categorical | 4943 | 3285 |  |  |  |  | 0.08931385270248934 | Cramers_V | 2.008520591604277e-09 |
| **demo_prnt_marital_v2** | categorical | 5304 | 3596 |  |  |  |  | 0.06980269424622064 | Cramers_V | 3.844176578019213e-07 |
| **demo_prnt_ed_v2** | continuous | 5324 | 3631 | 16.827385424492864 | 2.6172439052222587 | 16.5042687964748 | 2.953248378858894 | 0.11579961233016414 | Cohen_d | 9.87897130395815e-07 |
| **demo_sex_v2** | categorical | 5333 | 3635 |  |  |  |  | 0.04824564407537264 | Cramers_V | 3.6291967539779966e-05 |
| **demo_yrs_1** | categorical | 5250 | 3528 |  |  |  |  | 0.055498622150642454 | Cramers_V | 0.00012048796457501859 |
| **demo_gender_id_v2** | categorical | 5330 | 3632 |  |  |  |  | 0.050547179896417174 | Cramers_V | 0.0007014350567250117 |
| **fam_roster_2c_v2** | continuous | 5122 | 3422 | 2.21183131589223 | 2.6829891137146005 | 2.425189947399182 | 2.846986880190368 | -0.07713048683146864 | Cohen_d | 0.0021283072746262083 |
| **demo_prnt_prtnr_v2** | categorical | 5290 | 3586 |  |  |  |  | 0.03712122028646055 | Cramers_V | 0.0021283072746262083 |
| **fam_roster_3c_v2** | continuous | 4940 | 3291 | 3.4327935222672066 | 1.7411266636859943 | 3.579459130963233 | 2.0345026529247354 | -0.07745720301379563 | Cohen_d | 0.0025986133549755755 |
| **demo_ethn_v2** | categorical | 5272 | 3583 |  |  |  |  | 0.03558144615422856 | Cramers_V | 0.0027354306532459682 |
| **demo_fam_exp6_v2** | categorical | 5321 | 3621 |  |  |  |  | 0.03433776290589054 | Cramers_V | 0.0035953768678657198 |
| **demo_ed_v2** | categorical | 5333 | 3635 |  |  |  |  | 0.05274393722132513 | Cramers_V | 0.004514671773897042 |
| **demo_fam_exp2_v2** | categorical | 5316 | 3619 |  |  |  |  | 0.03257371624707411 | Cramers_V | 0.005488486760441504 |
| **demo_prnt_ethn_v2** | categorical | 5313 | 3611 |  |  |  |  | 0.029915203551750036 | Cramers_V | 0.011626303017065721 |
| **demo_roster_v2** | continuous | 5247 | 3510 | 4.773203735467886 | 1.799844978138692 | 4.685470085470086 | 1.5824233136433425 | 0.051771729737254586 | Cohen_d | 0.0352498244064192 |
| **demoi_p_select_language___1** | categorical | 5333 | 3635 |  |  |  |  | 0.025450866620086034 | Cramers_V | 0.0352498244064192 |
| **demo_origin_v2** | continuous | 5326 | 3630 | 186.74239579421706 | 16.378381302499893 | 185.91267217630855 | 19.935147796925968 | 0.04548013375247451 | Cohen_d | 0.0781020117692935 |
| **demo_prnt_16** | categorical | 5333 | 3635 |  |  |  |  | 0.02116270588283535 | Cramers_V | 0.08774753146356412 |
| **demo_prim** | categorical | 5333 | 3635 |  |  |  |  | 0.030339604284781396 | Cramers_V | 0.1529439909526628 |
| **demo_fam_exp4_v2** | categorical | 5322 | 3621 |  |  |  |  | 0.018088565207906197 | Cramers_V | 0.1535622755386761 |
| **demo_relig_v2** | continuous | 5122 | 3437 | 8.924834049199532 | 6.135902474908951 | 9.112307244690136 | 6.095253057753456 | -0.03065485671552125 | Cohen_d | 0.2761785607076003 |
| **demo_fam_exp3_v2** | categorical | 5312 | 3613 |  |  |  |  | 0.014202819573824315 | Cramers_V | 0.28903430927815055 |
| **demo_fam_exp1_v2** | categorical | 5303 | 3613 |  |  |  |  | 0.012862749715604218 | Cramers_V | 0.3461567160623354 |
| **demo_prnt_empl_v2** | continuous | 5313 | 3613 | 2.343873517786561 | 2.298209388554009 | 2.402435649045115 | 2.3675815869690124 | -0.025099992526235817 | Cohen_d | 0.3637797393171133 |
| **demo_fam_exp7_v2** | categorical | 5315 | 3620 |  |  |  |  | 0.01090183187038704 | Cramers_V | 0.4308745775552082 |
| **demo_prnt_income_v2** | categorical | 4902 | 3268 |  |  |  |  | 0.03155823327246903 | Cramers_V | 0.71318563767358 |
| **demo_fam_exp5_v2** | categorical | 5317 | 3619 |  |  |  |  | 0.006460447951613435 | Cramers_V | 0.7154113163417407 |
| **demo_prnt_gender_id_v2** | categorical | 5330 | 3633 |  |  |  |  | 0.018325181059167493 | Cramers_V | 0.8623866993136498 |
| **demo_prnt_gender_id_v2_l** | categorical | 5329 | 3633 |  |  |  |  | 0.018310978593189833 | Cramers_V | 0.8623866993136498 |
| **demo_child_time_v2** | categorical | 5304 | 3606 |  |  |  |  | 0.0034786118056934485 | Cramers_V | 0.8863798607745126 |
| **demo_brthdat_v2** | categorical | 5327 | 3633 |  |  |  |  | 0.019575432182453466 | Cramers_V | 0.9485200159944296 |
| **demo_yrs_2** | categorical | 5175 | 3493 |  |  |  |  | 0.008375519500126147 | Cramers_V | 0.9485200159944296 |
| **demo_race_a_p___0** | categorical | 5324 | 3627 |  |  |  |  | 0.0020410212567079145 | Cramers_V | 0.9485200159944296 |
| **demo_prnt_age_v2_l** | continuous | 5297 | 3597 | 40.114404379837644 | 6.523352643412216 | 40.12393661384487 | 6.839777841202479 | -0.0014262470623188448 | Cohen_d | 0.9485200159944296 |
| **demo_prnt_age_v2** | continuous | 5298 | 3597 | 40.11457153642884 | 6.523095506090046 | 40.12393661384487 | 6.839777841202479 | -0.0014012628032598988 | Cohen_d | 0.9485200159944296 |
| **acs_raked_propensity_score** | continuous | 5333 | 3635 | 680.2887822301182 | 342.79577597947434 | 680.9472593029932 | 345.82707351961807 | -0.0019124276624801217 | Cohen_d | 0.9485200159944296 |

**Table S6. Sensitivity analyses of subset1 examining associations between cluster assignment and sociodemographic variables after adjustment for race/ethnicity.**

| **Feature** | **Beta_cluster_adjusted_for_race** | **P_Value** | **Direction** | **P_FDR** |
| --- | --- | --- | --- | --- |
| **demo_ethn_v2** | 1.672273430841642e-15 | 3.090511406901508e-09 | Positive for label 1 | 1.0816789924155279e-07 |
| **fam_roster_2c_v2** | -0.6754093965278328 | 0.0005496715624694935 | Negative for label 1 | 0.009619252343216136 |
| **demo_prnt_marital_v2** | -0.3340045623130054 | 0.001979633643131313 | Negative for label 1 | 0.023095725836531987 |
| **demo_prnt_ethn_v2** | 0.035735527251285544 | 0.012367907193779976 | Positive for label 1 | 0.1082191879455748 |
| **demo_fam_exp4_v2** | 0.01844504598234753 | 0.036338090220581765 | Positive for label 1 | 0.25436663154407235 |
| **demo_origin_v2** | -2.3402548560258136 | 0.04841952462955053 | Negative for label 1 | 0.2824472270057114 |
| **fam_roster_3c_v2** | -0.25357749712363314 | 0.06918184335718833 | Negative for label 1 | 0.3459092167859416 |
| **demo_prnt_prtnr_v2** | -0.044960176520575657 | 0.0818357528466018 | Negative for label 1 | 0.3580314187038829 |
| **demo_fam_exp5_v2** | 0.02113762505709116 | 0.17853017355743045 | Positive for label 1 | 0.49958552623481844 |
| **demo_fam_exp3_v2** | 0.028440814378071923 | 0.1779485970817607 | Positive for label 1 | 0.49958552623481844 |
| **demo_fam_exp1_v2** | -0.02348471194476777 | 0.18801717411207583 | Negative for label 1 | 0.49958552623481844 |
| **demo_comb_income_v2** | 0.18422456653468564 | 0.20367785217788126 | Positive for label 1 | 0.49958552623481844 |
| **demo_prnt_gender_id_v2_l** | -0.032958861120437395 | 0.2283819548502027 | Negative for label 1 | 0.49958552623481844 |
| **demo_prnt_ed_v2** | 0.21605708830848844 | 0.20570328506797 | Positive for label 1 | 0.49958552623481844 |
| **acs_raked_propensity_score** | -36.90404848336624 | 0.14601976589972554 | Negative for label 1 | 0.49958552623481844 |
| **demo_prnt_gender_id_v2** | -0.032958861120437395 | 0.2283819548502027 | Negative for label 1 | 0.49958552623481844 |
| **demo_prnt_age_v2_l** | 0.4201613802338313 | 0.3866059074726865 | Positive for label 1 | 0.7517337089746683 |
| **demo_prnt_age_v2** | 0.4201613802338313 | 0.3866059074726865 | Positive for label 1 | 0.7517337089746683 |
| **demo_prnt_empl_v2** | -0.0973464939400699 | 0.5830111638409938 | Negative for label 1 | 0.8502246139347827 |
| **demo_brthdat_v2** | -0.023637952004449903 | 0.5607430471323076 | Negative for label 1 | 0.8502246139347827 |
| **demo_fam_exp2_v2** | 0.010244011445832316 | 0.49487161058885787 | Positive for label 1 | 0.8502246139347827 |
| **demo_gender_id_v2** | -0.023543826109493862 | 0.5614415022448924 | Negative for label 1 | 0.8502246139347827 |
| **demo_sex_v2** | -0.025118026418739663 | 0.5331180042452359 | Negative for label 1 | 0.8502246139347827 |
| **demo_ed_v2** | -0.04105962322209221 | 0.5057189831246147 | Negative for label 1 | 0.8502246139347827 |
| **demo_fam_exp6_v2** | -0.007323911095187981 | 0.6778196746246412 | Negative for label 1 | 0.9489475444744977 |
| **demo_fam_exp7_v2** | 0.0025640241274657947 | 0.9059907144814312 | Positive for label 1 | 0.9742140973022677 |
| **demo_roster_v2** | -0.01146316869025961 | 0.9463794088079172 | Negative for label 1 | 0.9742140973022677 |
| **demo_prim** | 0.012023653255097775 | 0.7997368735898688 | Positive for label 1 | 0.9742140973022677 |
| **demo_child_time_v2** | 0.0016067104556464276 | 0.9406728302469589 | Positive for label 1 | 0.9742140973022677 |
| **demo_yrs_1** | 0.00910709186682205 | 0.9321545228078265 | Positive for label 1 | 0.9742140973022677 |
| **demo_yrs_2** | 0.030482747767093067 | 0.7253818306596753 | Positive for label 1 | 0.9742140973022677 |
| **demo_prnt_income_v2** | 0.02786118729902794 | 0.8981682740163458 | Positive for label 1 | 0.9742140973022677 |
| **demo_prnt_16** | -0.004342966678679051 | 0.8930310522628322 | Negative for label 1 | 0.9742140973022677 |
| **demoi_p_select_language___1** | 0.0030890646996956633 | 0.8017585362967574 | Positive for label 1 | 0.9742140973022677 |
| **demo_relig_v2** | -0.005574479090828394 | 0.9909096560260692 | Negative for label 1 | 0.9909096560260692 |

**Table S7. Sensitivity analyses of subset2 examining associations between cluster assignment and sociodemographic variables after adjustment for race/ethnicity.**

| **Feature** | **Beta_cluster_adjusted_for_race** | **P_Value** | **Direction** | **P_FDR** |
| --- | --- | --- | --- | --- |
| **demo_ethn_v2** | 7.494005416219807e-16 | 7.549047575000448e-05 | Positive for label 1 | 0.0026421666512501567 |
| **demo_fam_exp2_v2** | -0.030618782551553182 | 0.005377332700996934 | Negative for label 1 | 0.09410332226744636 |
| **demo_prnt_ethn_v2** | 0.024475016751783568 | 0.03066777696084343 | Positive for label 1 | 0.35779073120984 |
| **acs_raked_propensity_score** | -36.08834964096172 | 0.06860564046072891 | Negative for label 1 | 0.40019956935425194 |
| **demo_prim** | 0.07275147539577774 | 0.05426827942430521 | Positive for label 1 | 0.40019956935425194 |
| **demo_comb_income_v2** | 0.21823908493528185 | 0.05873847757027531 | Positive for label 1 | 0.40019956935425194 |
| **demo_prnt_age_v2_l** | 0.5610935237990684 | 0.1482927999719959 | Positive for label 1 | 0.43252066658498806 |
| **demo_roster_v2** | -0.12917057526048492 | 0.14240248209772488 | Negative for label 1 | 0.43252066658498806 |
| **demo_fam_exp1_v2** | -0.02214145156483186 | 0.09132955758148796 | Negative for label 1 | 0.43252066658498806 |
| **demo_prnt_marital_v2** | -0.12345668411019387 | 0.1481832265872631 | Negative for label 1 | 0.43252066658498806 |
| **demo_prnt_prtnr_v2** | -0.03143159412363378 | 0.11161865405980223 | Negative for label 1 | 0.43252066658498806 |
| **demo_prnt_age_v2** | 0.5610935237990684 | 0.1482927999719959 | Positive for label 1 | 0.43252066658498806 |
| **demo_yrs_2** | -0.08572093805437679 | 0.23231013139008908 | Negative for label 1 | 0.6254503537425475 |
| **demo_prnt_16** | 0.027874400344403548 | 0.2804058955749831 | Positive for label 1 | 0.6962597509282864 |
| **demo_prnt_ed_v2** | 0.13602538569645817 | 0.3178065674861512 | Positive for label 1 | 0.6962597509282864 |
| **demo_yrs_1** | -0.08642480565390788 | 0.3182901718529309 | Negative for label 1 | 0.6962597509282864 |
| **demo_fam_exp3_v2** | -0.013969149653713958 | 0.37134727721226624 | Negative for label 1 | 0.7645385119076069 |
| **demo_prnt_empl_v2** | 0.10393208600627556 | 0.4628742810008897 | Positive for label 1 | 0.8105487023023831 |
| **demo_brthdat_v2** | -0.026217238344262393 | 0.421522507954345 | Negative for label 1 | 0.8105487023023831 |
| **demo_fam_exp6_v2** | -0.008881741575453938 | 0.46317068702993314 | Negative for label 1 | 0.8105487023023831 |
| **demo_relig_v2** | -0.27212335166501056 | 0.49579780071061363 | Negative for label 1 | 0.8263296678510228 |
| **demo_fam_exp5_v2** | -0.006645785616488124 | 0.56703455260507 | Negative for label 1 | 0.861605979932187 |
| **demo_fam_exp4_v2** | -0.0023537382708790563 | 0.5809940542195093 | Negative for label 1 | 0.861605979932187 |
| **demo_sex_v2** | -0.0173457953753125 | 0.5908155290963568 | Negative for label 1 | 0.861605979932187 |
| **demo_gender_id_v2** | -0.01278194286163975 | 0.6990490158102772 | Negative for label 1 | 0.8862052310515698 |
| **demo_fam_exp7_v2** | -0.005337787167963903 | 0.7397150452808923 | Negative for label 1 | 0.8862052310515698 |
| **fam_roster_3c_v2** | -0.03052932623532769 | 0.7596044837584883 | Negative for label 1 | 0.8862052310515698 |
| **demo_origin_v2** | 0.31583786620926907 | 0.7067445924734812 | Positive for label 1 | 0.8862052310515698 |
| **demo_prnt_income_v2** | 0.07979795084812566 | 0.6478309324000271 | Positive for label 1 | 0.8862052310515698 |
| **demoi_p_select_language___1** | 0.003807688144712093 | 0.7119328620426186 | Positive for label 1 | 0.8862052310515698 |
| **demo_ed_v2** | 0.008997583036563762 | 0.8536215858843071 | Positive for label 1 | 0.9250258669612609 |
| **fam_roster_2c_v2** | -0.0274982026892832 | 0.860471770135345 | Negative for label 1 | 0.9250258669612609 |
| **demo_child_time_v2** | -0.002885573458917123 | 0.8815227768317535 | Negative for label 1 | 0.9250258669612609 |
| **demo_prnt_gender_id_v2_l** | 0.0020306010570495985 | 0.9250258669612609 | Positive for label 1 | 0.9250258669612609 |
| **demo_prnt_gender_id_v2** | 0.0020306010570495985 | 0.9250258669612609 | Positive for label 1 | 0.9250258669612609 |

**Table S8. Associations between race/ethnicity and all sociodemographic variables included in the post-hoc analyses in subset 1.** Continuous variables were tested using one-way ANOVA and categorical variables using chi-square tests. Effect sizes are reported as eta-squared for continuous variables and Cramér’s V for categorical variables. P values were corrected for multiple comparisons using false discovery rate (FDR) correction.

| **Variable** | **Variable_type** | **Test** | **Statistic** | **P_value** | **Effect_size** | **Effect_size_type** | **N** | **P_FDR** |
| --- | --- | --- | --- | --- | --- | --- | --- | --- |
| **demo_prnt_ethn_v2** | categorical | Chi-square | 1491.1045253003372 | 0.0 | 0.8576837261484102 | Cramers_V | 2027 | 0.0 |
| **demo_ethn_v2** | categorical | Chi-square | 2027.0000000000005 | 0.0 | 1.0 | Cramers_V | 2027 | 0.0 |
| **demo_prnt_16** | categorical | Chi-square | 471.0021727721376 | 1.250290238117019e-100 | 0.4820416684165963 | Cramers_V | 2027 | 1.4586719444698553e-99 |
| **demo_prnt_marital_v2** | categorical | Chi-square | 471.29161781124566 | 2.9358684199936384e-87 | 0.2410948801197084 | Cramers_V | 2027 | 2.5688848674944337e-86 |
| **demo_comb_income_v2** | categorical | Chi-square | 519.1706541510933 | 6.08849570579993e-87 | 0.2530452550513107 | Cramers_V | 2027 | 4.2619469940599514e-86 |
| **demoi_p_select_language___1** | categorical | Chi-square | 301.59926626672814 | 4.895783215927822e-64 | 0.3857343065345053 | Cramers_V | 2027 | 2.8558735426245626e-63 |
| **demo_prnt_ed_v2** | continuous | ANOVA | 77.14793542106088 | 5.838669731078554e-61 | 0.13240918010858135 | eta_squared | 2027 | 2.919334865539277e-60 |
| **acs_raked_propensity_score** | continuous | ANOVA | 66.6275079898528 | 5.1454973016826205e-53 | 0.1164556974789519 | eta_squared | 2027 | 2.2511550694861464e-52 |
| **demo_prnt_prtnr_v2** | categorical | Chi-square | 186.681846288283 | 2.736857996258318e-39 | 0.30347587296240297 | Cramers_V | 2027 | 1.0643336652115683e-38 |
| **fam_roster_2c_v2** | continuous | ANOVA | 38.65325630860102 | 3.237042537574168e-31 | 0.07103376826376927 | eta_squared | 2027 | 1.1329648881509589e-30 |
| **demo_prnt_age_v2** | continuous | ANOVA | 29.58563614931865 | 6.043003286743904e-24 | 0.05529140412407232 | eta_squared | 2027 | 1.7625426253003055e-23 |
| **demo_prnt_age_v2_l** | continuous | ANOVA | 29.58563614931865 | 6.043003286743904e-24 | 0.05529140412407232 | eta_squared | 2027 | 1.7625426253003055e-23 |
| **demo_fam_exp3_v2** | categorical | Chi-square | 106.4057100006796 | 4.249284551196766e-22 | 0.22911609180234863 | Cramers_V | 2027 | 1.144038148399129e-21 |
| **demo_prnt_income_v2** | categorical | Chi-square | 168.40055384656532 | 5.104033366425341e-19 | 0.14411689203602743 | Cramers_V | 2027 | 1.2760083416063353e-18 |
| **demo_fam_exp2_v2** | categorical | Chi-square | 86.84108697958698 | 6.1699469371373716e-18 | 0.2069835117561737 | Cramers_V | 2027 | 1.4396542853320535e-17 |
| **demo_fam_exp7_v2** | categorical | Chi-square | 64.02776239497969 | 4.1232971680071255e-13 | 0.17772858693580548 | Cramers_V | 2027 | 9.019712555015587e-13 |
| **demo_prim** | categorical | Chi-square | 84.86334967239141 | 2.1856921626426646e-11 | 0.10230649709501792 | Cramers_V | 2027 | 4.499954452499604e-11 |
| **demo_yrs_2** | categorical | Chi-square | 74.11782675976916 | 5.392287012619795e-11 | 0.1104012108507175 | Cramers_V | 2027 | 1.0485002524538491e-10 |
| **demo_fam_exp5_v2** | categorical | Chi-square | 49.431655197696905 | 4.745182976634413e-10 | 0.15616212212198669 | Cramers_V | 2027 | 8.741126535905498e-10 |
| **demo_fam_exp4_v2** | categorical | Chi-square | 43.64408540661144 | 7.606095671089678e-09 | 0.14673571214551429 | Cramers_V | 2027 | 1.3310667424406937e-08 |
| **demo_origin_v2** | continuous | ANOVA | 9.299575789725406 | 1.9081831344588167e-07 | 0.01806445892162905 | eta_squared | 2027 | 3.180305224098028e-07 |
| **demo_yrs_1** | categorical | Chi-square | 56.67029701549646 | 1.8850927446456205e-06 | 0.08360280990677818 | Cramers_V | 2027 | 2.999011184663487e-06 |
| **demo_prnt_gender_id_v2** | categorical | Chi-square | 47.818495400875996 | 3.3613650406179532e-06 | 0.08867688965138015 | Cramers_V | 2027 | 4.901990684234515e-06 |
| **demo_prnt_gender_id_v2_l** | categorical | Chi-square | 47.818495400875996 | 3.3613650406179532e-06 | 0.08867688965138015 | Cramers_V | 2027 | 4.901990684234515e-06 |
| **fam_roster_3c_v2** | continuous | ANOVA | 7.58764564967274 | 4.583660496337775e-06 | 0.014788205707165445 | eta_squared | 2027 | 6.417124694872885e-06 |
| **demo_fam_exp1_v2** | categorical | Chi-square | 28.812756572270573 | 8.532768506023959e-06 | 0.11922450573920224 | Cramers_V | 2027 | 1.148641914272456e-05 |
| **demo_fam_exp6_v2** | categorical | Chi-square | 24.247757892247805 | 7.124075120268879e-05 | 0.10937269643440231 | Cramers_V | 2027 | 9.234912192941138e-05 |
| **demo_relig_v2** | continuous | ANOVA | 5.901086900706341 | 0.00010155631277621834 | 0.011539058191036095 | eta_squared | 2027 | 0.00012694539097027292 |
| **demo_child_time_v2** | categorical | Chi-square | 10.879626021158774 | 0.027950686820212147 | 0.07326222584084377 | Cramers_V | 2027 | 0.03373358754163535 |
| **demo_gender_id_v2** | categorical | Chi-square | 14.848960566140876 | 0.062149725891780916 | 0.06052100828263959 | Cramers_V | 2027 | 0.07250801354041107 |
| **demo_prnt_empl_v2** | continuous | ANOVA | 2.167959227295409 | 0.07025229841656908 | 0.004270427526281644 | eta_squared | 2027 | 0.07931711111548122 |
| **demo_ed_v2** | categorical | Chi-square | 22.93273932503124 | 0.11554440871453794 | 0.05318278758714258 | Cramers_V | 2027 | 0.12637669703152588 |
| **demo_roster_v2** | continuous | ANOVA | 1.6421888395056357 | 0.16096562876386822 | 0.003238123105599875 | eta_squared | 2027 | 0.17072112141622386 |
| **demo_sex_v2** | categorical | Chi-square | 5.305036170261877 | 0.2574058468452757 | 0.051158440878739664 | Cramers_V | 2027 | 0.2649766070466073 |
| **demo_brthdat_v2** | categorical | Chi-square | 22.319678356422962 | 0.3234189865436604 | 0.05246710430913793 | Cramers_V | 2027 | 0.3234189865436604 |

**Table S9. Associations between race/ethnicity and all sociodemographic variables included in the post-hoc analyses in subset 2.** Continuous variables were tested using one-way ANOVA and categorical variables using chi-square tests. Effect sizes are reported as eta-squared for continuous variables and Cramér’s V for categorical variables. P values were corrected for multiple comparisons using false discovery rate (FDR) correction.

| **Variable** | **Variable_type** | **Test** | **Statistic** | **P_value** | **Effect_size** | **Effect_size_type** | **N** | **P_FDR** |
| --- | --- | --- | --- | --- | --- | --- | --- | --- |
| **demo_prnt_ethn_v2** | categorical | Chi-square | 1506.7368024585649 | 0.0 | 0.8587851276521601 | Cramers_V | 2043 | 0.0 |
| **demo_ethn_v2** | categorical | Chi-square | 2043.0 | 0.0 | 1.0 | Cramers_V | 2043 | 0.0 |
| **demo_prnt_16** | categorical | Chi-square | 496.86762508992877 | 3.18802279776953e-106 | 0.4931580953847645 | Cramers_V | 2043 | 3.719359930731118e-105 |
| **demo_prnt_marital_v2** | categorical | Chi-square | 469.9934286951039 | 5.481515032626483e-87 | 0.23981796528351335 | Cramers_V | 2043 | 4.7963256535481725e-86 |
| **demo_comb_income_v2** | categorical | Chi-square | 500.0127925648 | 4.6574770604009284e-83 | 0.24735823811703975 | Cramers_V | 2043 | 3.26023394228065e-82 |
| **demo_prnt_ed_v2** | continuous | ANOVA | 93.99156803669341 | 1.9023404248566056e-73 | 0.15574628216011724 | eta_squared | 2043 | 1.1096985811663533e-72 |
| **demoi_p_select_language___1** | categorical | Chi-square | 326.7345046153763 | 1.8463715271449813e-69 | 0.3999109694209809 | Cramers_V | 2043 | 9.231857635724906e-69 |
| **acs_raked_propensity_score** | continuous | ANOVA | 69.7242633344431 | 2.1445266463229428e-55 | 0.1203752462527361 | eta_squared | 2043 | 9.382304077662875e-55 |
| **demo_prnt_prtnr_v2** | categorical | Chi-square | 191.23588370410096 | 2.87549928966505e-40 | 0.305950037113962 | Cramers_V | 2043 | 1.1182497237586307e-39 |
| **fam_roster_2c_v2** | continuous | ANOVA | 34.908936254314305 | 3.1180616204497425e-28 | 0.06412263636687755 | eta_squared | 2043 | 1.0913215671574099e-27 |
| **demo_prnt_age_v2** | continuous | ANOVA | 29.406340635288092 | 8.331533722268212e-24 | 0.05456670003292672 | eta_squared | 2043 | 2.4300306689948952e-23 |
| **demo_prnt_age_v2_l** | continuous | ANOVA | 29.406340635288092 | 8.331533722268212e-24 | 0.05456670003292672 | eta_squared | 2043 | 2.4300306689948952e-23 |
| **demo_prnt_income_v2** | categorical | Chi-square | 169.44695052428565 | 3.355608116411122e-19 | 0.14399675315700994 | Cramers_V | 2043 | 9.034329544183791e-19 |
| **demo_fam_exp2_v2** | categorical | Chi-square | 82.30288993100153 | 5.66196259737685e-17 | 0.2007120095650787 | Cramers_V | 2043 | 1.4154906493442123e-16 |
| **demo_fam_exp3_v2** | categorical | Chi-square | 74.16190597836689 | 2.9967777896168575e-15 | 0.19052688104970666 | Cramers_V | 2043 | 6.992481509106001e-15 |
| **demo_fam_exp1_v2** | categorical | Chi-square | 62.5204166110923 | 8.561132074791708e-13 | 0.17493501571021505 | Cramers_V | 2043 | 1.872747641360686e-12 |
| **demo_fam_exp5_v2** | categorical | Chi-square | 47.025934319152896 | 1.5060659602497105e-09 | 0.15171710013185538 | Cramers_V | 2043 | 3.1007240358082273e-09 |
| **demo_prim** | categorical | Chi-square | 73.18935038487223 | 2.739239618701847e-09 | 0.09463673907050167 | Cramers_V | 2043 | 5.326299258586925e-09 |
| **demo_origin_v2** | continuous | ANOVA | 10.86510299829942 | 1.0227170084461033e-08 | 0.02087976871564879 | eta_squared | 2043 | 1.883952383979664e-08 |
| **demo_fam_exp7_v2** | categorical | Chi-square | 38.87041665409331 | 7.409268588933507e-08 | 0.13793529709283534 | Cramers_V | 2043 | 1.2966220030633637e-07 |
| **demo_yrs_2** | categorical | Chi-square | 50.013356453619714 | 1.3895844773006913e-06 | 0.09033336535123393 | Cramers_V | 2043 | 2.3159741288344855e-06 |
| **demo_relig_v2** | continuous | ANOVA | 7.987169306015705 | 2.1861898919217076e-06 | 0.01543452626415248 | eta_squared | 2043 | 3.4780293735118075e-06 |
| **demo_yrs_1** | categorical | Chi-square | 44.871015058210276 | 0.00014525509654959092 | 0.07410009556523249 | Cramers_V | 2043 | 0.0002210403643145949 |
| **demo_roster_v2** | categorical | Chi-square | 73.93900196825187 | 0.00019882061422006465 | 0.0951201689722394 | Cramers_V | 2043 | 0.0002899467290709276 |
| **fam_roster_3c_v2** | continuous | ANOVA | 5.086078210473527 | 0.0004450449345846946 | 0.009883823962281739 | eta_squared | 2043 | 0.0006230629084185724 |
| **demo_fam_exp6_v2** | categorical | Chi-square | 15.685140293866857 | 0.003472110794144013 | 0.08762136620090333 | Cramers_V | 2043 | 0.004673995299809248 |
| **demo_fam_exp4_v2** | categorical | Chi-square | 14.936674965623649 | 0.004834375714365552 | 0.08550525016046812 | Cramers_V | 2043 | 0.0062667833334368265 |
| **demo_ed_v2** | categorical | Chi-square | 25.646783974994882 | 0.177796930859271 | 0.056021183295701396 | Cramers_V | 2043 | 0.22224616357408875 |
| **demo_brthdat_v2** | categorical | Chi-square | 24.74150548148277 | 0.2114999313854344 | 0.05502358489724247 | Cramers_V | 2043 | 0.25525853787897257 |
| **demo_gender_id_v2** | categorical | Chi-square | 14.792373090512028 | 0.2529878772831308 | 0.049127437896439946 | Cramers_V | 2043 | 0.2895243118716689 |
| **demo_sex_v2** | categorical | Chi-square | 5.315431373990712 | 0.2564358190863353 | 0.051007621698733345 | Cramers_V | 2043 | 0.2895243118716689 |
| **demo_prnt_gender_id_v2_l** | categorical | Chi-square | 11.35891131152436 | 0.4984359124289336 | 0.04305003844750808 | Cramers_V | 2043 | 0.5286441495458387 |
| **demo_prnt_gender_id_v2** | categorical | Chi-square | 11.35891131152436 | 0.4984359124289336 | 0.04305003844750808 | Cramers_V | 2043 | 0.5286441495458387 |
| **demo_prnt_empl_v2** | continuous | ANOVA | 0.6756079171961633 | 0.6088705998001422 | 0.0013242654229479295 | eta_squared | 2043 | 0.6267785586177935 |
| **demo_child_time_v2** | categorical | Chi-square | 2.5663662070477966 | 0.6327926129695269 | 0.0354425638723584 | Cramers_V | 2043 | 0.6327926129695269 |

Abbreviations

| Abbreviation | Full Name |
| --- | --- |
| STG | Superior Temporal Gyrus |
| cACC | Caudal Anterior Cingulate Cortex |
| Ins | Insula |
| TTG | Transverse Temporal Gyrus |
| SMG | Supramarginal Gyrus |
| cMFG | Caudal Middle Frontal Gyrus |
| PostC | Postcentral Gyrus |
| PreC | Precentral Gyrus |
| Cu | Cuneus |
| MTG | Middle Temporal Gyrus |
| Cau | Caudate |
| Tha | Thalamus Proper |
| VAN | Ventral Attention Network |
| SN | Salience Network |
| SMNm | Sensorimotor Mouth Network |
| RSPN | Retrosplenial Network |
| VN | Visual Network |
| FPN | Frontoparietal Network |
| DAN | Dorsal Attention Network |
| DN | Default Network |
| CON | Cingulo-Opercular Network |
| CPN | Cingulo-Parietal Network |
| Put | Putamen |
| Pal | Pallidum |
| Amyg | Amygdala |
| Hipp | Hippocampus |
| Acc | Accumbens |
| vDC | Ventral Diencephalon |
| BS | Brain Stem |
| Cere | Cerebellum |
| rACC | Rostral Anterior Cingulate Cortex |
| TP | Temporal Pole |
| FG | Fusiform Gyrus |
| ITG | Inferior Temporal Gyrus |
| IOFC | Inferior Orbitofrontal Cortex |
| mOFC | Medial Orbitofrontal Cortex |
| pOrb | Pars Orbitalis |
| LOFC | Lateral Orbitofrontal Cortex |
| LG | Lingual Gyrus |
| pOper | Pars Opercularis |
| PCu | Precuneus |
| LO | Lateral Occipital Cortex |
| IC | Isthmus Cingulate |
| SFG | Superior Frontal Gyrus |
| SPG | Superior Parietal Gyrus |
| NA | Not Assigned |
| lh | Left Hemisphere |
| rh | Right Hemisphere |
